# Correction: Is pedagogical training an essential requirement for inclusive education? The case of faculty members in the area of Social and Legal Sciences in Spain

**DOI:** 10.1371/journal.pone.0306372

**Published:** 2024-06-27

**Authors:** Rosario Lopez-Gavira, Inmaculada Orozco, Ana Doménech

The ORCID iD is missing for the second author. Please see the author’s ORCID iD here:

• Author Inmaculada Orozco’s ORCID iD is https://orcid.org/0000-0001-5170-7805
