# Supplementary material for: Is pedagogical training an essential requirement for inclusive education? The case of faculty members in the area of Social and Legal Sciences in Spain
Source: PLoS One. 2021 Jul 2;16(7):e0254250. doi: 10.1371/journal.pone.0254250 (PMC8253417; doi:10.1371/journal.pone.0254250)
Supplement: S1 File — (ZIP) [file pone.0254250.s001.zip › 1.4. PERCEPCIαN DEL ESTUDIANTE (1).rtf]

Documento:		4. Ciencias Sociales y Jurídicas\P1 CCSS Creencias
Peso:	0
Posición:	28 - 29
Código:	1. Creencias\Rol docente y actitudes\1.4. Percepción del estudiante
E: Y eso, cómo crees tú que te ven tus estudiantes.
P1: Que me preocupo más allá de la asignatura.


Documento:		4. Ciencias Sociales y Jurídicas\P2 CCSS Creencias
Peso:	0
Posición:	32 - 33
Código:	1. Creencias\Rol docente y actitudes\1.4. Percepción del estudiante
E: Cómo crees que te ven tus estudiantes.
P2: A mí como un profesor duro, y nunca ha sido mi intención que me vieran de esa manera, pero como un profesor exigente y duro. Y no es cierto, ni mi índice de suspensos lo dice ni nada por el estilo. Pero creo que me ven así porque yo con ellos sigo cierto protocolo a la hora de trabajar con ellos, como suelo utilizar el correo de la universidad para preservar la protección de datos, esto es una facultad de derecho y en breve tengo alumnos en prácticas externas. A todos les hablo de usted aunque tengan 19 años porque ellos tienen que saber comportarse, pero difícilmente ellos me hablan a mí de usted, por ejemplo, porque no saben, no están acostumbrados. Ellos lo ven así y también creo que me ven como un profesor duro de una manera comparativa porque me ven una serie de compañeros y compañeras que quizá no explican el temario íntegro en su totalidad, que quizá no van siempre a clase y que luego en los exámenes son diferentes porque te dejan las leyes, las cosas, etc.


Documento:		4. Ciencias Sociales y Jurídicas\P3 CSS Creencias
Peso:	0
Posición:	26 - 27
Código:	1. Creencias\Rol docente y actitudes\1.4. Percepción del estudiante
E: Vale. ¿Cómo crees que te ven tus estudiantes? 
P3: Hombre, yo creo que me ven como una persona cercana y que se preocupa de los problemas que pueden tener dentro del ámbito de estudio, dentro de la universidad. Creo que saben que yo siempre les doy su sitio y que los escucho.


Documento:		4. Ciencias Sociales y Jurídicas\P4 CCSS Creencias
Peso:	0
Posición:	30 - 31
Código:	1. Creencias\Rol docente y actitudes\1.4. Percepción del estudiante
E: Sí, sí, nos referimos a eso. Y cómo crees que te ven tus estudiantes.
P4: Yo creo que me ven como una persona que pongo de mi parte, que me tomo mi trabajo en serio e intento hacer las cosas lo mejor posible.


Documento:		4. Ciencias Sociales y Jurídicas\P4 CCSS Creencias
Peso:	0
Posición:	56 - 59
Código:	1. Creencias\Rol docente y actitudes\1.4. Percepción del estudiante
E: ¿Y crees que la universidad, en un momento dado, puede suponer una barrera para que las personas con discapacidad estudien una carrera y la acaben con éxito?
P4: Yo creo que sí.
E: Por qué.
P4: Porque yo creo que el tema de la discapacidad, pues desde épocas muy tempranas supone trabas, desde muy pequeño. En la universidad, pues a medida que va aumentando el nivel de exigencia, ¿no? El nivel de estudio, pues las cosas se complican. Entonces, ya estamos en el nivel más alto. Luego, dentro de la universidad, pues también creo que he escuchado muchas veces a estudiantes decir que estaban estudiando esta carrera porque no habían podido estudiar otra, porque… Entonces, ahí se rompen muchos sueños, ¿no? Porque todo el mundo en principio tiene, digamos, toda la libertad del mundo, si se esfuerza, tiene una nota alta para estudiar la carrera que quiera, pero en el caso de los estudiantes con discapacidad, pues yo recuerdo que los estudiantes con discapacidad visual, en concreto un caso, pues no había podido estudiar informática o matemáticas. Son carreras que requieren mucha pizarra, mucho… Y que por el tema de los problemas de visión no lo habían podido afrontar. Y creo que, en general, todavía se pueden encontrar muchas trabas a la hora de lo que para otros es normal, pues para ellos, llegar a una clase que no te estés enterando de nada porque no puedes ver y no has tenido el material anticipadamente o te lo han pasado, pero resulta que no es accesible… Entonces, ese tipo de cuestiones, son problemas.


Documento:		4. Ciencias Sociales y Jurídicas\P5 CSS Creencias
Peso:	0
Posición:	38 - 39
Código:	1. Creencias\Rol docente y actitudes\1.4. Percepción del estudiante
E: Y cómo crees que te ven tus estudiantes.
P5: Yo creo que me ven enrollado, pero para bien y para mal, ¿no? Yo creo que aquí el listo sabe que conmigo…y, además, yo esto son cosas que digo en clase, ¿no? De “yo soy un tío muy flexible, si tengo un problema, lo hablamos, si hay que cambiar un examen de día yo lo cambio, si hay que un tema… Lo que queráis, ¿no? Pero necesito que el otro se comprometa”, ¿no? Con el que no viene a clase, se porta mal, no se le ocurre de cuando viene me revienta la clase, ¿no? Pues ese tampoco tiene… ¿no? 


Documento:		4. Ciencias Sociales y Jurídicas\P6 CCSS Creencias
Peso:	0
Posición:	20 - 21
Código:	1. Creencias\Rol docente y actitudes\1.4. Percepción del estudiante
E: Y, ¿cómo crees que te ven tus estudiantes?
P6: Pues yo creo que me ven cercana o, por lo menos, es lo que ellos me transmiten, hay una diferencia cuando hablo con ellos o interactúo o incluso cuando mis compañeros me cuentan sus experiencias con los alumnos. Yo creo que la palabra que más puede definir mi relación con ellos es la cercanía. No me gustan las barreras de ningún tipo, tampoco las que te posicionan en una situación de preferencia por así llamarla o superior a su situación actual, no me gustan esas discrepancias y, por tanto, procuro estar al mismo nivel que ellos siempre.


Documento:		4. Ciencias Sociales y Jurídicas\P7 CCSS Creencias
Peso:	0
Posición:	32 - 33
Código:	1. Creencias\Rol docente y actitudes\1.4. Percepción del estudiante
E: ¿Cómo te ven tus alumnos?
P7: ¿Mis alumnos? Pues yo creo que me ven precisamente como una persona humana y, desde que llevo, desde el 2003 que comencé, simplemente doy un seguimiento, sigo teniendo contacto con todos mis antiguos alumnos, les pongo felicitaciones, a los más recientes les mando oportunidades de trabajo y mi percepción, porque siempre tengo mucha inquietud por saber, tanto ahora cuando voy dando clases como después, pues en qué puedo mejorar, pues yo creo que son muy sinceros y que están satisfechos con la asignatura. De hecho, me expresan, sobre todo, me expresan que no solo han aprendido a nivel técnico sino también humano, de cómo orientarse profesionalmente, saber que pueden acudir a mí con dificultades profesionales cuando terminen, que me ven accesible y, sobre todo, yo creo que me ven que, o esa es mi percepción, que les he aportado algo durante el trayecto de su carrera, sobre todo como persona y como pensar, articular el pensamiento, organizarse…


Documento:		4. Ciencias Sociales y Jurídicas\P7 CCSS Creencias
Peso:	0
Posición:	119 - 119
Código:	1. Creencias\Rol docente y actitudes\1.4. Percepción del estudiante
 El ver ese refuerzo, además no es que lo finja, es que es verdad que ves que las opiniones que te están dando son incluso más valiosas que las tuyas. El seguir contando con antiguos alumnos, enviando convocatorias, felicitaciones de navidad… Desde que llevo desde el 2003, pues les felicito siempre las navidades a todos. Pues eso, ese afecto, ese verte, hace también que acudan a ti muchas veces, que tengan una percepción también una percepción de la universidad Autónoma positiva, un feedback continuo y no puntual. El darles un feedback “oye, mira”. En los trabajos no solo darles la nota, sino que en Moodle les pongo un comentario largo a cada uno. Eso lleva mucho tiempo, pero el darles un feedback sobre “deberías mejorar o te animo a que participes más en clase porque tienes mucho que aportar”. Hacer comentarios así, sí que a veces me doy cuenta que, por ser tan exigente, los comentarios eran muy hirientes, porque decían “no, eso no lo has…”. Me he dado cuenta que los comentarios debería de ser más suaves y más en positivo, pero siempre dar un feedback continuo. Luego, tanto a trabajos, exámenes y exposiciones, luego en Moodle mandarles cómo pueden mejorar, qué reiteran o qué podrían hacer dentro del escenario en la clase para exponer mejor…


Documento:		4. Ciencias Sociales y Jurídicas\P8 CCSS Creencias
Peso:	0
Posición:	30 - 34
Código:	1. Creencias\Rol docente y actitudes\1.4. Percepción del estudiante
E: ¿Y cómo percibes tú que te ven ellos?
P8: Pues yo te diría que ahora mismo una de las grandes satisfacciones que tengo son las encuestas de los estudiantes. No tanto las preguntas cerradas, sino las preguntas abiertas, o sea, los calificativos son “lo mejor que nos ha pasado en los cuatro años que llevamos en la casa, no sabemos cómo agradecer todo lo que haces por nosotros, por fin he entendido para qué sirve la economía”, cosas de ese tipo. También hay cosas muy negativas como “habla muy deprisa, quiere que aprendamos mucho, es muy exigente, no nos pasa una”, ¿vale? Pero de media estoy híper y súper contenta. No te lo puedes ni imaginar. Eso me da mucho…vaya.
E: Entonces, no te habrá extrañado que además te haya elegido este alumno con discapacidad, ¿no?
P8: Vamos a ver, me ha extrañado porque no contaba con un estudio de estas características. Para mí fue un reto enfrentarme a algo nuevo, nuevísimo. Tengo que reconocerte que, en algunas sesiones de las individuales, alguna vez pasé un poco de miedo porque él sobre-reaccionaba a la crítica, eso sí lo hacía, ¿vale? Imagínate que le estás contando algo y te decía “no, no, que te estás liando, esto no puede ser”. Se ponía un poco…
E: Nervioso.


Documento:		4. Ciencias Sociales y Jurídicas\P8 CCSS Creencias
Peso:	0
Posición:	66 - 67
Código:	1. Creencias\Rol docente y actitudes\1.4. Percepción del estudiante
E: P8, ¿qué actitudes personales crees tú que ayudaron? Tuyas, personales tuyas.
P8: Sí. Pues yo creo que una actitud de cercanía. Yo creo que eso le ayudó, o sea, me sentía como una persona dispuesta a apoyarle en lo que hiciera falta siempre y cuando tuviese como compensación ver que respondía positivamente. Eso también se lo dije “mira, si yo estoy aquí dejándome la piel para que las cosas te vayan sobre ruedas, que no tengas ningún problema con la asignatura, si empiezas en plan pasota, paso yo más que tú, que yo pasar también sé”. Y entonces, yo creo que se creó esa especie de magia de “tú estás apostando por mí y no voy a defraudarte y aquí estoy al cien por cien y vamos a terminar teniendo una relación más allá casi de profesor-alumno, más casi de amistad”.


Documento:		4. Ciencias Sociales y Jurídicas\P9 CCSS Creencias
Peso:	0
Posición:	32 - 33
Código:	1. Creencias\Rol docente y actitudes\1.4. Percepción del estudiante
E: ¿Así crees que también te ven tus estudiantes? Cercana como has dicho.
P9: Yo creo que sí y te digo que creo que sí porque cuando leo los comentarios que ponen en mis evaluaciones, así lo ponen.


Documento:		4. Ciencias Sociales y Jurídicas\P10 CCSS Creencias
Peso:	0
Posición:	33 - 36
Código:	1. Creencias\Rol docente y actitudes\1.4. Percepción del estudiante
E: Y, tú cómo crees que te ven a ti tus estudiantes.
P10: No lo sé, yo lo único que sé es que, parece ser, que me ven como buen profesor; te lo digo porque, a veces, hay cosas puntuales, que te sorprenden. Hace poco, un estudiante me manda un correo electrónico “no sé si te acordarás de mí, has sido profesor mío y quería hablar contigo y tal si estás por la facultad”, y digo, “uy, no sé, qué querrá”, miré, y digo “uy, este chico ha venido a tutorías, no me acordaba ya de él”, lo había tenido hacía unos años. Y, simplemente, quería decirme que había encontrado trabajo y que venía a decírmelo porque suponía que me alegraría muchísimo, y digo “claro, me alegro muchísimo”. Entonces, esas cosas que dices no sé…y bueno, luego me han pedido que participe en alguna promoción, no en la fiesta oficial, sino en la extraoficial que montan, pues me han pedido que fuera a hablar y cosas de esas. No sé, pues entiendo que me tienen cierto aprecio.
E: Hombre, claro que sí.
P10: Hace ya muchos, muchos años, la promoción, y no era en esta universidad siquiera, cuando acabaron, aprobados y suspendidos, me invitaron a cenar y, no sé, me hicieron unas tarjetitas, una de las chicas y otra de los chicos, así como muy monas y muy simpáticas. Entonces, entiendo que, no sé, que no es lo habitual. Supongo que, pues eso, les caeré en gracia o alguna cosa de estas. No sé, pero bueno, intento tomármelo en serio y cuando la gente no funciona me cabreo y me da mucha rabia, es decir, no suelo ir regalando los aprobados a nadie, absolutamente a nadie, pero, por otro lado, pues procuro currármelo, no sé.


Documento:		4. Ciencias Sociales y Jurídicas\P11 CCSS Creencias
Peso:	0
Posición:	33 - 36
Código:	1. Creencias\Rol docente y actitudes\1.4. Percepción del estudiante
E: De eso trataba también la siguiente pregunta, de cómo te ven tus alumnos, que me has dicho que como una persona exigente, pero que les gusta la cercanía que tienes con ellos, ¿no?
P11: Y el elemento motivador, esa es otra característica que tengo, yo soy un motivante nato. Entonces…
E: Y, ¿crees que ellos te ven así también?
P11: Sí. Yo hago, digamos, a principio de curso y luego al final me lo acaban reconociendo, hago de entrenador exigente: “si tú puedes conseguir un 8, te voy a pedir un 8, no me des un 5 y si no, te voy a perseguir”, y les persigo, pero luego me lo agradecen y no van por el 5, van por el 8 o más. 


Documento:		4. Ciencias Sociales y Jurídicas\P12 CCSS Creencias
Peso:	0
Posición:	34 - 35
Código:	1. Creencias\Rol docente y actitudes\1.4. Percepción del estudiante
E: Vale. ¿Cómo crees que te ven tus estudiantes? 
P12: Digamos que como un profesor muy duro. Ésta es la información que ellos me hacen llegar cuando terminan los estudios, por ejemplo, he terminado una asignatura este año y de manera informal, pues me ha llegado esta valoración de los estudiantes, “pues,  no es tan duro como lo pintaban”. Yo exijo unos contenidos mínimos, unas habilidades mínimas, y no bajo el listón.


Documento:		4. Ciencias Sociales y Jurídicas\P15 CCSS Creencias
Peso:	0
Posición:	36 - 37
Código:	1. Creencias\Rol docente y actitudes\1.4. Percepción del estudiante
E: Vale. Y cómo crees que te ven tus estudiantes.
P15: Yo creo que me ven bien, que soy exigente, pero bien.


Documento:		4. Ciencias Sociales y Jurídicas\P16 CCSS Creencias
Peso:	0
Posición:	24 - 25
Código:	1. Creencias\Rol docente y actitudes\1.4. Percepción del estudiante
E: Bueno, pues cómo crees que te ven los estudiantes.
P16: A mí la docencia me gusta, yo disfruto mucho dando clases. Entonces cuando uno disfruta de lo que hace, pues imagino que la impresión que tiene que el receptor tiene que ser positiva, pero en la docencia como en cualquier otra actividad. Yo el único referente que tengo es las valoraciones que hace el alumno y afortunadamente desde el 2009, todas las valoraciones de los alumnos están por encima de la media, por lo cual, estoy totalmente satisfecho de esa percepción por parte del alumno.


Documento:		4. Ciencias Sociales y Jurídicas\P17 CCSS Creencias
Peso:	0
Posición:	52 - 73
Código:	1. Creencias\Rol docente y actitudes\1.4. Percepción del estudiante
E: Y, ¿cómo crees que te ven tus estudiantes a ti?
P17: Uf, eso sí que es complicado.
E: ¿Cómo crees que te pueden ver?
P17: Eso sí que es complicado, porque yo no tengo ni puñetera idea. No tengo ni idea.
E: Pero, ¿tú qué percibes cuando das clase?
P17: Yo percibo que soy capaz de captar su atención, consigo que atiendan, que es lo que yo voy buscando, les quito los móviles, los ordenadores, las Tablets, y los apuntes se los damos después enteros, no guardo nada. Y lo que consigo es que presten atención y que participen en clase. A lo mejor me ven como un peñazo porque les hago salir a la pizarra, les hago levantarse, les insisto en que respondan… Y me gusta activarlos, muchas veces me ven muy pesado o “ya está aquí el borde de economía”, porque intento tener un lenguaje también medio científico medio llano y porque, muchas veces, cuando noto que se están viniendo un poquito abajo, el otro día les solté, se me ocurrió sobre la marcha, hablando de la economía negativa y positiva, que qué pensaban de las corridas de toros, el “buuuu”, ya estaban todos despiertos, ya estaban todos pendientes, “venga, manos arriba, quién está a favor y quién está en contra, manos arriba”, y ya estaban todos pendientes y mirando a ver qué va a decir este o el otro. Había una alumna que se estaba echando las manos a la cabeza de lo que decían los otros compañeros… Eso me gusta, ¿no? Pero no sé lo que piensan, la verdad es que yo creo que piensan eso “ya está aquí otra vez”, pero que no es tan serio como otros profesores, por ejemplo. 
E: A lo mejor les gusta eso.
P17: No lo sé, no se lo he preguntado nunca. 
E: A lo mejor están deseando que llegues.
P17: A lo mejor este año me animo y les hago una encuesta.
E: Claro, qué es lo que más les gusta y lo que menos, para ver qué dicen.
P17: Porque, además, ellos no participan en las encuestas que les hace la UPO. 
E: Ah, ¿no? Y, ¿por qué?
P17: Yo no lo sé, mira que se les dice, y, además, hay cinco o seis respuestas…
E: Esas encuestas, por lo menos, las que son cuantitativas, ¿no? que es lo que te gusta, que te pongan tres palabras y…
P17: No, no, pero es que ni las cuantitativas las responden, no entran, no entran. 
E: A nosotros nos obligan.
P17: Sí, a nosotros nos obligan, pero a los alumnos no. Te dan cinco respuestas, seis respuestas. Yo no les publicaba las notas hasta que no respondiesen. 
E: ¿Cuántos son?, ¿cuántos estudiantes?
P17: Yo tengo este año 65.
E: Son un montón, pero no participan.
P17: No.


Documento:		4. Ciencias Sociales y Jurídicas\P17 CCSS Creencias
Peso:	0
Posición:	80 - 83
Código:	1. Creencias\Rol docente y actitudes\1.4. Percepción del estudiante
E: Entonces, tú crees que, al ser muy dinámico, lo que me estás comentando, tu experiencia, ¿no? con la propia empresa, pues hace que influya sí o sí en…
P17: Yo creo que sí. De hecho, tengo algún alumno antiguo, de cuando daba clase en la escuela de turismo, que ahora es proveedor mío.
E: Ah, ¿sí?
P17: Sí. En una empresa que tenemos familiar, el proveedor mío es un antiguo alumno de turismo, y él sí que me lo dijo en su día, me dijo “no veas lo que nos acordamos de ti”, cuando él y su pareja se fueron a Londres a vivir, montaron la empresa en Londres y después se vinieron a Sevilla con la empresa, y dice “no veas lo que nos hemos acordado de ti, de lo que nos explicabas en economía y de los conceptos…”, porque después las otras asignaturas que tenían de economía, digamos, en empresariales, eran contabilidad… Y la contabilidad es más mecánica, la contabilidad no les da para la actualidad, no les da para analizar una empresa, no les da para hablar de conceptos empresariales, sino de una mecánica, de un sistema. Entonces, eso me dijeron esos alumnos, que bueno, que gracias a algunas de las cosas que yo les había explicado, se habían dado cuenta de dónde tenían ellos que trabajar.


Documento:		4. Ciencias Sociales y Jurídicas\P17 CCSS Diseños
Peso:	0
Posición:	19 - 24
Código:	1. Creencias\Rol docente y actitudes\1.4. Percepción del estudiante
P17: Para mí, la implicación, el tenerlos constantemente hablando, que ellos hagan parte de la clase, para mí esa es la estrategia fundamental, para mí…una de las principales quejas que me han hecho en la encuesta es que no tienen los apuntes antes de la clase, o sea, a ellos les encantaría tener mis gráficas y mis datos antes de la clase e ir apuntando cosas encima, pero claro, así yo no les puedo preguntar, porque claro, yo tengo preparado un movimiento y antes del movimiento les obligo a pensar y a discutir, y a decir uno una idea y se la arrebato y a decir otro otra idea y “te estás acercando”…
E: Y qué cosas han dicho que les gustan de ti.
P17: Pues han dicho que les doy muchos ejemplos, que los acerco mucho a la vida real y, entonces, lo ven más claramente, que tengo respuestas para todo, les gusta que soy puntual, les gusta que me preparo la clase, por ejemplo, tengo aquí una explicación con muchos ejemplos que es útil para el futuro, la forma de explicar es muy didáctica y constructiva, “en la forma de explicar ejemplos es muy bueno”, “con ejemplos para todo”, este dice que los ejemplos sirven para entender mejor la teoría, “contenidos justos y necesarios”…
E: Y, ¿negativo?
P17: No poner los Power Point antes de empezar, que se repite mucho: “a veces no da tiempo a tomar apuntes”, “con más ejemplos se entendería mejor”, doy muchísimos ejemplos. Negativo: “no dar el temario antes de empezar el tema”, “no tener los apuntes antes” …
E: Sí.


Documento:		4. Ciencias Sociales y Jurídicas\P18 CCSS Creencias
Peso:	0
Posición:	42 - 43
Código:	1. Creencias\Rol docente y actitudes\1.4. Percepción del estudiante
E: Y, en general, cómo crees que te pueden ver tus alumnos, desde tu punto de vista.
P18: Yo creo que me ven como muy teórica, yo soy muy teórica, pero también, las asignaturas que he impartido hasta ahora son muy teóricas, excepto dos o tres que me han permitido ser mucho más práctica, lo que pasa es que, como tenemos divididas la parte teórica y la práctica, sí es cierto que la parte práctica prefiero dejar todas aquellas cuestiones, pero sí, por ejemplo, lo que quiero es que lean mucho, simplemente periódicos o informaciones que estén actualizados y en temas de empleo, por ejemplo, hoy estábamos hablando de cuáles son las características del mercado de trabajo en Andalucía, y entonces, eso lo han tenido que buscar ellos, lo ponemos en común… En fin. Lo que pasa es que mi asignatura este año es muy teórica y tengo que dar unas nociones básicas, que me hacen preparármela, pasar mucho tiempo preparándomela, buscar información… Incentivarlos también un poco a ellos.


Documento:		4. Ciencias Sociales y Jurídicas\P19 CCSS Creencias
Peso:	0
Posición:	28 - 29
Código:	1. Creencias\Rol docente y actitudes\1.4. Percepción del estudiante
E: ¿Cómo crees que te ven tus estudiantes?
P19: Uff, pues…no sé. Por ejemplo, aquí hacemos encuestas, y cuando nos mandan los resultados de las encuestas suelo estar orgulloso porque me ponen en muy buen lugar mis alumnos. Luego, está la asignación del padrino, en la Universidad de Burgos, que es el profesor o profesora que cuando llega el Acto de Graduación, es el que da el discurso este final de Graduación ante los padres y le pone las becas a los alumnos, y he sido elegido varias veces como padrino. Creo, y lo he hablado con el Decano, que la figura del padrino debe cambiarse, invitando a un político, o a algún antiguo alumno que esté trabajando en un puesto importante… También en las orlas suelo estar siempre. Los profesores en las orlas los eligen los alumnos y casi siempre estoy entre los diez profesores que eligen.  Otras ocasiones me han regalado al final de curso una placa de agradecimiento, o botellas de vino, y yo les digo, “pero por qué hacéis esto, si a mí ya me pagan por daros clase”, pero sin duda me hacen sentirme feliz. Creo que deberíais preguntarles a los alumnos cómo me ven, y no a mí.


Documento:		4. Ciencias Sociales y Jurídicas\P20 CCSS Creencias
Peso:	0
Posición:	32 - 33
Código:	1. Creencias\Rol docente y actitudes\1.4. Percepción del estudiante
E: Entonces, cómo crees que te ven tus estudiantes. Me imagino que bien porque si te eligen como madrina y tal…
P20: Sí, yo creo que me ven bien, porque intento ser cercana, ¿sabes? Mostrarles la confianza necesaria para que podamos establecer una buena relación en clase.


Documento:		4. Ciencias Sociales y Jurídicas\P21 CCSS Creencias
Peso:	0
Posición:	48 - 53
Código:	1. Creencias\Rol docente y actitudes\1.4. Percepción del estudiante
E: En estas fechas ya. Y, ¿cómo cree que le ven sus estudiantes?
P21: Uf, ahí hay de todo. Yo, en ese sentido, tengo cierta satisfacción de no tener la impresión de que me vean, o por lo menos, ellos lo que me transmiten, independientemente de…que no me gusta mencionar, porque como soy el primero que reconozco que el sistema de evaluación, en ese sentido, es un desastre, pero a mí me han dado en repetidas ocasiones el premio este que dan a la excelencia docente por las evaluaciones de los alumnos. 
E: Qué bien.
P21: Pero no lo considero que es un mérito especial, porque es un criterio de un sistema de evaluación poco fiable. Pero, en el cara a cara, en el día a día con los alumnos, percibo que me ven de una manera positiva. Si bien, que también percibo que hay alumnos a los que les caigo como el culo y me pondrán verde porque, evidentemente, el tener un alumnado tan heterogéneo da pie a todo. Exactamente es lo que nos pasa a los profesores, yo hay alumnos con los que empatizo, y hay alumnos con los que no, a los que mataría…
E: Por no volverlos a ver.
P21: Por no volverlos a ver, efectivamente, ¿no? Pero, vamos, en líneas generales, estoy contento porque, además, creo que es necesario, la relación con los alumnos es recíproca. Si tú con los alumnos en clase no ves que hay una correspondencia y que los alumnos responden mínimamente a tus propuestas, a tus dinámicas, a…es muy difícil, es que no se puede dar clase, aunque haya…yo he tenido profesores que lo han hecho, y podría decir que conozco compañeros que lo hacen, que ponen una especie de muro invisible y dan la clase como si no hubiera alumnos en el aula, ¿no? Pero independientemente, la relación es recíproca, el feedback, pero sí, yo me retroalimento un poco de, no voy a decir del cariño de los alumnos, pero sí de la respuesta positiva de que los alumnos empatizan contigo y están con tu dinámica. Y, cuando tú eso lo vives, cuando eso realmente se produce, eso recarga muchísimo las pilas, ¿no? Incluso, yo he tenido periodos de dudas, de tener la sensación de que estoy haciendo esto muy mal, cuando es una continua experimentación y con esta metodología, con esta dinámica de trabajo, y cuando he pensado que me estaba equivocando y que los alumnos eso lo estaban valorando y me hacen un simple comentario de que qué bien esto, eso, puf, es lo que más me carga las pilas, ¿no?


Documento:		4. Ciencias Sociales y Jurídicas\P22 CCSS Creencias
Peso:	0
Posición:	30 - 31
Código:	1. Creencias\Rol docente y actitudes\1.4. Percepción del estudiante
E: Y, ahora, ¿cómo cree que la ven sus alumnos?
P22: Yo creo que, de la misma manera, pues que me ven, pues eso, seria, seca, exigente, pero, según va pasando el curso nos vamos relajando todos, tanto ellos como yo, eso lo noto. Y, sobre todo, que empatizas más con unos que con otros.


Documento:		4. Ciencias Sociales y Jurídicas\P23 CCSS Creencias
Peso:	0
Posición:	38 - 39
Código:	1. Creencias\Rol docente y actitudes\1.4. Percepción del estudiante
E: Estupendo. Y, ¿cómo cree que la ven sus estudiantes?
P23: Pues yo creo que cercana también. Pero también creo que el problema, en cierto sentido, es que también soy un poco blandengue porque me mueven el corazoncito a la hora de evaluar, ¿no? Porque pienso que se sienten muy cercanos a mí, muy familiares, me cuentan los problemas que tienen y que no tienen nada que ver con la asignatura, vienen al despacho y me cuentan cosas...


Documento:		4. Ciencias Sociales y Jurídicas\P24 CCSS Creencias
Peso:	0
Posición:	26 - 27
Código:	1. Creencias\Rol docente y actitudes\1.4. Percepción del estudiante
E: Bueno, ya me has hablado de la cercanía que tienen contigo, pero cómo crees que te ven tus estudiantes, aunque se ve que están muy contentos por lo que comentas.
P24: Normalmente sí. A ver, cómo te diría, me he enterado por ellos, no sé si es verdad o no, que hay estudiantes que no cogen la asignatura porque dicen que hay que trabajar mucho, que por esa parte es buena señal, porque aquí se viene a trabajar, no a aprobar. 


Documento:		4. Ciencias Sociales y Jurídicas\P24 CCSS Creencias
Peso:	0
Posición:	27 - 27
Código:	1. Creencias\Rol docente y actitudes\1.4. Percepción del estudiante
s decir, creo que la asignatura les gusta porque participan, porque la construimos entre todos.


Documento:		4. Ciencias Sociales y Jurídicas\P24 CCSS Creencias
Peso:	0
Posición:	35 - 35
Código:	1. Creencias\Rol docente y actitudes\1.4. Percepción del estudiante
Entonces, intento llegar al máximo y a ver, ojalá llegara al 100%, pero hay estudiantes que me han dicho “P. 78, he salido llorando de tus clases”, y digo “por qué, qué ha pasado”, dice “es que me haces tanto reflexionar que me mueves mucho” y digo “muchas gracias, eso es lo que quiero, que mueva ahí y se produzca un cambio”. 


Documento:		4. Ciencias Sociales y Jurídicas\P25 CCSS Creencias
Peso:	0
Posición:	46 - 47
Código:	1. Creencias\Rol docente y actitudes\1.4. Percepción del estudiante
E: Entonces, todo esto es lo que tú crees que te define, ¿no? y ¿cómo crees que te ven tus estudiantes?
P25: Yo creo que ellos me ven…aquí hay imágenes como de estereotipo, ¿no?, aquí como en todas las instituciones y como en todos los grupos, se trasmiten de unos a otros los rumores ¿no? como, por ejemplo, este profesor no sé cuánto, esta profesora no sé qué. Me pasó esto con una compañera que tuve, que era muy buena amiga mía, y todos tenían la imagen de que era terrorífica, y luego cuando la conocían se les caía esa imagen. Yo creo que la imagen que tienen de mí es que soy muy exigente. Yo tengo un alumno que me dice continuamente, tiene una práctica delante muy fácil, y siempre me dice “es que tú buscas las complicaciones”, y yo le digo “me quieres decir dónde está la complicación”, claro y es, porque como ellos creen que siempre busco las complicaciones y ven algo muy fácil, pues se lían más porque buscan las complicaciones. Entonces, la imagen es esa, y yo sé que lo soy, yo sé que soy exigente.
