# Supplementary material for: Is pedagogical training an essential requirement for inclusive education? The case of faculty members in the area of Social and Legal Sciences in Spain
Source: PLoS One. 2021 Jul 2;16(7):e0254250. doi: 10.1371/journal.pone.0254250 (PMC8253417; doi:10.1371/journal.pone.0254250)
Supplement: S1 File — (ZIP) [file pone.0254250.s001.zip › 1.5. ACTITUDES (1).rtf]

Documento:		4. Ciencias Sociales y Jurídicas\P1 CCSS Creencias
Peso:	0
Posición:	32 - 33
Código:	1. Creencias\Rol docente y actitudes\1.5. Actitudes
E: Vale. Y, por último, en este apartado, cómo crees que influye tu rol como docente en el aprendizaje del alumnado.
P1: Sí influye porque un docente, cuando le interesa mucho su asignatura, su campo de conocimiento y se preocupa porque ese conocimiento llegue al alumnado, el alumnado lo asimile y lo trabaje… Yo intento, y hablo de mi experiencia particular, tengan discapacidad o no, eso ya me da un poco igual, que participe activamente en la generación de ese conocimiento. Que ese conocimiento le sirva para algo, que vayamos más allá de los contenidos teóricos, sino que introduzcamos contenidos prácticos, que ellos vean que lo que están aprendiendo aquí les sirve para algo… Y al mismo tiempo, para mí es muy importante trabajar las competencias verticales, es decir, el conocimiento, sino también las transversales, que van a influir en su inserción laboral: comunicación, expresión, dominio de idiomas extranjeros… Vemos vídeos en inglés en clase… Hacemos muchas cosas que, en principio, parece que “uh, esto no sé a qué viene”, pero que a la hora de desarrollar determinadas competencias laborales o profesionales les va a venir bien. Es verdad que en una asignatura tú puedes hacer cosas limitadas, pero cada granito de arena suma. Entonces, para mí eso es muy importante a la hora de diseñar mis clases. Y los alumnos están super contentos, eso se ve en las valoraciones. O sea, yo disfruto en clase, ellos disfrutan, aprendemos, sacan buenas notas, y además aprenden cosas que les van a servir para su futuro profesional, con lo que todos ganamos.


Documento:		4. Ciencias Sociales y Jurídicas\P2 CCSS Creencias
Peso:	0
Posición:	34 - 35
Código:	1. Creencias\Rol docente y actitudes\1.5. Actitudes
E: Vale. Y tú cómo crees que influye la figura del profesor en el aprendizaje del estudiante.
P2: Mucho. Y de hecho, mis mayores satisfacciones me las he llevado siempre cuando los alumnos han dejado de ser ya mis alumnos. Yo he recibido correos de antiguos alumnos que ni me acordaba que me han dicho que los han cogido porque ellos sabían hacer los escritos procesales que…o me he encontrado alumnos en el gimnasio o por la calle que me dicen que lo que han estudiado conmigo no se les olvida. Entonces, mi mayor satisfacción es esa, ¿no?


Documento:		4. Ciencias Sociales y Jurídicas\P3 CSS Creencias
Peso:	0
Posición:	28 - 29
Código:	1. Creencias\Rol docente y actitudes\1.5. Actitudes
E: ¿Cómo crees que esto influye en el aprendizaje de los estudiantes? 
P3: Creo que eso es fundamental para ellos. Cuando ven a un profesor cercano, que puedan motivarlo, con lo complicado que es motivar a los alumnos hoy en día en clase, pues, yo creo que el alumno al final lo agradece, porque por circunstancia todos nos hemos encontrado alumnos y que después de X años se acuerdan de nosotros y eso es un alago. Yo creo que esto es importantísimo y que la actitud del profesor influye en el alumno.


Documento:		4. Ciencias Sociales y Jurídicas\P4 CCSS Creencias
Peso:	0
Posición:	32 - 35
Código:	1. Creencias\Rol docente y actitudes\1.5. Actitudes
E: Y estas características que tienes, ¿crees que influyen de alguna manera en el alumnado?
P4: Yo creo que sí, claro.
E: De qué manera crees que influyen.
P4: Yo creo que influyen, claro, la actitud del profesorado, la preparación del profesorado, la manera de transmitir los contenidos… influyen en su aprendizaje, en que aprendan más o que aprendan menos. Luego ya es por su parte también, que no solo influye lo que haga el profesor, sino que también transmitirles la base del conocimiento que luego eso también tienen que profundizarlo estudiando mucho.


Documento:		4. Ciencias Sociales y Jurídicas\P5 CSS Creencias
Peso:	0
Posición:	40 - 41
Código:	1. Creencias\Rol docente y actitudes\1.5. Actitudes
E: Y cómo crees que influye tu actitud en el aprendizaje del alumnado.
P5: Pues no te lo sé decir. Hombre, yo imagino que todo lo que tú hagas por acercarte a ellos, ¿no? O por tratar de entender cómo viven ellos la asignatura y de qué manera pueden contar contigo para superar lo que tienen que… ¿no? Los exámenes correspondientes, yo creo que tienen que…que eso les puede enganchar, ¿no? No sé, pero la verdad es que no tengo ni idea, no sé…


Documento:		4. Ciencias Sociales y Jurídicas\P6 CCSS Creencias
Peso:	0
Posición:	22 - 23
Código:	1. Creencias\Rol docente y actitudes\1.5. Actitudes
E: Y, cómo crees que influye tu actitud o tus características en el aprendizaje del alumnado. 
P6: Yo creo que ellos se sienten con confianza. Muchas veces, en clase, a mí me ha pasado que, dependiendo del profesor, pues tienes el temor a interrumpirlo o incluso el temor dentro de la clase a que te señalen como “este siempre está preguntando”, o “este nunca entiende las cosas”, o… Y yo intento desde el minuto uno que llego decirles que estamos aquí para aprender, la primera yo, que nos equivocamos todos, que podemos equivocarnos y volvernos a equivocar y que, al final, empezar una carrera y una asignatura es un proceso, que no necesitamos ser los más rápidos aprendiendo ni llegar el primero a la meta y que se sientan totalmente libres y con confianza hacia mí para interrumpir cuando quieran, para decirme que no están de acuerdo con lo que digo, para rebatirme cualquier idea, para volverme a preguntar cien veces, pero que si salen de clase sin haber entendido lo que yo he explicado, que tengan claro que es un día perdido teniéndome a mí allí.


Documento:		4. Ciencias Sociales y Jurídicas\P7 CCSS Creencias
Peso:	0
Posición:	34 - 35
Código:	1. Creencias\Rol docente y actitudes\1.5. Actitudes
E: Entonces, tú crees que nuestra actitud influye a la hora de que el alumno aprenda.
P7: Muchísimo más la actitud que incluso los conocimientos. A lo mejor los conocimientos, siendo un tutor, que decir por donde tienen que ir, e incluso ese aprendizaje mucho mejor, creo que influye mucho más la actitud como persona, el saberles orientar, el ser un referente o ejemplo en tu comportamiento en seriedad, en cómo les tratas como personas. Yo creo que eso les hace mucho más, les marca mucho más, y en una edad en la que están modelando mucho su personalidad, yo creo que esto es lo que más les marca, la actitud del profesor.


Documento:		4. Ciencias Sociales y Jurídicas\P8 CSS Diseños
Peso:	0
Posición:	12 - 13
Código:	1. Creencias\Rol docente y actitudes\1.5. Actitudes
E: Y con respecto al rol docente y las actitudes, cómo crees que influyen las características que tú tienes como docente en el aprendizaje del alumnado.
P8: Yo creo que muy positiva, porque estoy a su total disposición. Soy una profesora a la antigua usanza, donde la docencia es para mí muy importante. No solo la parte investigadora, que es la que se valora de una manera más prioritaria.


Documento:		4. Ciencias Sociales y Jurídicas\P10 CCSS Creencias
Peso:	0
Posición:	37 - 38
Código:	1. Creencias\Rol docente y actitudes\1.5. Actitudes
E: Ya. Y, ¿crees que esa actitud que tú tienes, aunque no sepas bien a qué características se debe, influye en el aprendizaje del alumnado?
P10: Quiero creer que sí, si no, no lo haría claro. No lo hago simplemente porque me sienta estupendo y diga ala, es que esto lo he hecho yo…no sé, lo hago pensando en ellos y en que son lo más importante…muchas veces cambio las presentaciones porque, al revisarlas digo, “esto no se entiende”, no sé, pero cuando ya está hecha, pues está hecha, no sé, y a lo mejor lo modifico porque tengo la impresión de que no se acaba de pillar la idea. Hoy mismo venía un estudiante, porque claro, “es que las transparencias eso está así”, claro, pero es que las transparencias es una cosa dinámica y mis transparencias se mueven. Entonces, no es lo mismo cuando tú lo coges después de dos o tres meses a estudiar y ves una foto y dices…claro, el tema es, que yo lo digo muchas veces en clase, “fijaros qué es lo que hay en la transparencia, que eso, aunque os quedéis con la foto, cuando lo imprimáis es una foto, pero lo mío no es una foto, es una pequeña peliculita, y claro, el tema es que tenéis que fijaros mucho más en lo que digo, y esto es un apoyo a”. Entonces, pues estoy pensando, a lo mejor, eso cómo se podría toquetear de alguna manera para, en algunos casos, si se puede evitar, inducir ese error, pues eso. Entonces, cuando lo vuelvo a revisar para el año que viene digo, “pues esto…”, me acordaré de la duda y diré, “pues esto lo podía poner”, y ya está.


Documento:		4. Ciencias Sociales y Jurídicas\P11 CCSS Creencias
Peso:	0
Posición:	37 - 38
Código:	1. Creencias\Rol docente y actitudes\1.5. Actitudes
E: Y bueno, ya también me estás contestando un poco a esta, cómo crees que influyen tus actitudes en su aprendizaje. Bien porque hace que quieran más, ¿no?
P11: Claro, ahí hay otro elemento que yo creo que valoran mucho y es que yo he tenido otro trabajo fuera de la universidad y a mí me gusta decir que hay dos tipos de profesores, aunque está feo generalizar, pero por lo que yo he visto, ¿eh? Los que han hecho carrera universitaria, investigación y tienen toda la parte dogmática muy bien agarrada y luego están los que han tocado la calle, y claro, eso en una carrera económica, que hayas tenido empleados, que hayas tenido que cuadrar cuentas, que tienes que pagar y no tienes tesorería… Esa experiencia personal, lógicamente, un profesor que no ha tocado esto no lo tiene, pero tú sí. Entonces, los alumnos yo creo que también valoran mucho las anécdotas y esas experiencias de la calle, que para eso somos profesores asociados, para dar ese valor añadido. Entonces, poder explicar todas estas cosas, yo creo que eso los alumnos también lo valoran porque les abre los ojos a una realidad que no está en los libros, sino que es la tuya.


Documento:		4. Ciencias Sociales y Jurídicas\P12 CCSS Creencias
Peso:	0
Posición:	36 - 37
Código:	1. Creencias\Rol docente y actitudes\1.5. Actitudes
E: ¿Cómo crees que influye tu actitud o tus características en el aprendizaje del alumnado?
P12: Bueno yo creo que lo que hago es enfrentarlo a la vida real, y ésta es muy exigente, ya que las segundas oportunidades no existen y aquí en la universidad sí existe esa segunda, tercera y cuarta. Pero vamos, hay que aprovecharla. Entonces, lo que yo trato de fomentar mucho es el aprendizaje por uno mismo. 


Documento:		4. Ciencias Sociales y Jurídicas\P13 CCSS Creencias
Peso:	0
Posición:	33 - 34
Código:	1. Creencias\Rol docente y actitudes\1.5. Actitudes
E: Porque tú cómo crees que influye en el aprendizaje del alumnado.
P13: Es fundamental porque es él el que apaga las expectativas o las acrecienta, las ganas de conocimiento. Tú topas con un mal docente y ahí se cierra todo ya. Condiciona tu forma de ver las cosas. Para el estudiante es amar algo y descubrir sus posibilidades de futuro, o provocar que caiga en el olvido, o tomarlo como algo puramente obligatorio para alcanzar el título. El que guste una disciplina o no creo que guarda mucha relación con las capacidades de comunicación del docente. Y ahí van muchas cosas. No solamente es el lenguaje hablado, sino la expresión, amable, relajada, no estar siempre cabreado, aunque no siempre lo consigues, pero la comunicación es todo, tiene que ser efectiva para que el estudiante se involucre con la asignatura.


Documento:		4. Ciencias Sociales y Jurídicas\P14 CCSS Creencias
Peso:	0
Posición:	30 - 31
Código:	1. Creencias\Rol docente y actitudes\1.5. Actitudes
E: Y esa actitud que tú tienes de cercanía y de accesibilidad, ¿cómo crees que influye en el aprendizaje de los estudiantes?
P14: Pues, si no fuese así, yo creo que desmotivaría a muchos estudiantes, es decir, la asignatura por sí misma, es desmotivante y si no tienes cuidado en cómo la enseñas, pues puede desmotivar, por eso intento ser muy cuidadoso con eso.


Documento:		4. Ciencias Sociales y Jurídicas\P15 CCSS Creencias
Peso:	0
Posición:	38 - 41
Código:	1. Creencias\Rol docente y actitudes\1.5. Actitudes
E: Y cómo crees que influyen las características que tiene el profesor en el aprendizaje de sus estudiantes.
P15: Hombre, yo creo que influye, claro.
E: De qué manera crees que puede influir.
P15: Hombre si las clases pueden ser, digamos, amenas o agradables y motivar más o menos… También influye qué tipo de asignatura sea… En fin.


Documento:		4. Ciencias Sociales y Jurídicas\P16 CCSS Creencias
Peso:	0
Posición:	26 - 27
Código:	1. Creencias\Rol docente y actitudes\1.5. Actitudes
E: ¿Cómo crees que el profesor influye en el aprendizaje de los estudiantes? 
P16: Mucho. Todos los que hemos sido alumnos sabemos que se puede coger fobia  a una materia por el entusiasmo del profesor, o nos ha gustado mucho. Para mí la física cuántica es bastante indigerible, independientemente del docente, pero claro si el docente te entusiasma, pues eso ayuda.


Documento:		4. Ciencias Sociales y Jurídicas\P17 CCSS Creencias
Peso:	0
Posición:	74 - 83
Código:	1. Creencias\Rol docente y actitudes\1.5. Actitudes
E: Y, de tus características, de tu actitud, ¿tú crees que influye en su aprendizaje?, cómo eres tú, ¿crees que puede influir en su aprendizaje?
P17: Sí. Yo creo que sí influye. Yo soy activo. Yo, además, trabajo en Endesa, en un área muy rara que se llama Regulación, y nosotros nos encargamos de batallar con la administración, principalmente y después con algunos clientes que tenemos, que, bueno, no porque sean clientes, sino porque son casos muy específicos de gente que les gusta luchar, mucho. Entonces, realmente, estamos todo el día recibiendo muchísima información, tenemos que gestionar muchísima información y tenemos que ser muy ágiles, y es lo que yo intento que ellos se incorporen a ese concepto, es decir, “señores, que esto está vivo, que esto es ágil, es muy complejo, que la economía es un sistema en el que las teorías del caos son aplicables y que, además, hay mucha psicología y muchos pensamientos", y les insisto mucho en el comportamiento de los directivos, y de los políticos, que vean a las personas como son, que son personas, y la psicología influye mucho en nuestros problemas. Entonces, intento despertarlos, en cierto sentido, los veo todavía muy adormilados, como muy niños, y necesito que reaccionen. 
E: ¿Qué año es?  
P17: Primero. Es que no leen ni periódicos. Ven internet, lo que esté en internet…
E: Y más en una carrera como esta, ¿no?
P17: No leen periódicos. El otro día, cuatro, de 65. Decían que leían periódicos, que esa es otra, vamos, es decir piensa mal y…
E: Entonces, tú crees que, al ser muy dinámico, lo que me estás comentando, tu experiencia, ¿no? con la propia empresa, pues hace que influya sí o sí en…
P17: Yo creo que sí. De hecho, tengo algún alumno antiguo, de cuando daba clase en la escuela de turismo, que ahora es proveedor mío.
E: Ah, ¿sí?
P17: Sí. En una empresa que tenemos familiar, el proveedor mío es un antiguo alumno de turismo, y él sí que me lo dijo en su día, me dijo “no veas lo que nos acordamos de ti”, cuando él y su pareja se fueron a Londres a vivir, montaron la empresa en Londres y después se vinieron a Sevilla con la empresa, y dice “no veas lo que nos hemos acordado de ti, de lo que nos explicabas en economía y de los conceptos…”, porque después las otras asignaturas que tenían de economía, digamos, en empresariales, eran contabilidad… Y la contabilidad es más mecánica, la contabilidad no les da para la actualidad, no les da para analizar una empresa, no les da para hablar de conceptos empresariales, sino de una mecánica, de un sistema. Entonces, eso me dijeron esos alumnos, que bueno, que gracias a algunas de las cosas que yo les había explicado, se habían dado cuenta de dónde tenían ellos que trabajar.


Documento:		4. Ciencias Sociales y Jurídicas\P18 CCSS Creencias
Peso:	0
Posición:	44 - 47
Código:	1. Creencias\Rol docente y actitudes\1.5. Actitudes
E: Y cómo crees que tus características, tanto personales como profesionales, influyen en el aprendizaje de tu alumnado.
P18: Hombre, yo creo que, aunque yo sea una persona seria, también son una persona cercana, y yo creo que ellos no tienen miedo de exponer sus opiniones porque yo, además, no soy de las personas que les digan que es un error, que qué está usted diciendo… No, simplemente, les digo “bueno, esto es así, pero, ¿estás seguro?”, entonces, intento que razonen esa respuesta que me ha dado, entonces, yo creo que soy seria, pero también un poco cercana y esa cercanía les permite a ellos, incluso si dan una respuesta negativa, contraria o que no sea la respuesta que yo espero, al menos, que sí puedan reflexionar un poquito, pero es quizá eso, porque soy…aunque sea una persona, ya te digo, seria, pero también cercana. Ellos pueden venir a las tutorías a la hora que quieran, me mandan un correo “mira P18”, y es una tutoría que no esté, pues intento darte una hora de tutorías o buscarte un horario asequible. Yo creo que eso es lo que me define. Tampoco soy la profesora de “yo estoy aquí y tú estás ahí”, no, es una relación…
E: Un poco horizontal.
P18: Sí.


Documento:		4. Ciencias Sociales y Jurídicas\P19 CCSS Creencias
Peso:	0
Posición:	30 - 31
Código:	1. Creencias\Rol docente y actitudes\1.5. Actitudes
E: ¿Cómo crees que influye tu actitud o características en el aprendizaje del alumnado?
P19: Pues, si un profesor va con una actitud positiva, de querer implicarse en la clase, de que le gusta su trabajo…, esa actitud se refleja en el alumnado, porque es así, el alumnado capta lo que el profesor les da. En cambio, si eres un docente desganado, que no se prepara las clases y va ahí a echar el rato, pues el alumnado va a estar allí de la misma forma que está el profesor. Es así, la actitud del profesorado se refleja en el alumnado, influye así.


Documento:		4. Ciencias Sociales y Jurídicas\P20 CCSS Creencias
Peso:	0
Posición:	34 - 35
Código:	1. Creencias\Rol docente y actitudes\1.5. Actitudes
E: Y cómo crees que influye tu actitud o características en el aprendizaje del alumnado, si crees que puede influir de alguna manera.
P20: Pues yo creo que sí que influye, pero influye a todos, es decir, si tú tienes un profesor que no se para a explicarte determinadas cosas o no se preocupa en exceso de que adquieras determinados conocimientos o habilidades, pues al final terminas por no hacerle caso o no hacer caso a esa asignatura o tal. Entonces, intentar involucrarte a esa asignatura de alguna manera para que el aprendizaje sea más claro. Por ejemplo, poniendo ejemplos que les sirvan o, según han ido pasando los años he ido viendo que los alumnos son más visuales, no son tanto...entonces, bueno, pues ponerles algún corto que tenga que ver con la administración, por ejemplo, pues eso les llama muchísimo la atención, algunas cosas así específicas. O la búsqueda de noticias… Cosas de estas que les puedan llamar la atención. Hay que ir buscando ese aspecto, o yo por lo menos lo creo, que no te tienes que quedar ahí anclado en los apuntes, sino buscar alternativas.


Documento:		4. Ciencias Sociales y Jurídicas\P21 CCSS Creencias
Peso:	0
Posición:	54 - 55
Código:	1. Creencias\Rol docente y actitudes\1.5. Actitudes
E: Qué bien. Y, ¿cómo cree que influye su actitud o sus características en el aprendizaje del alumnado? Esas características que me está diciendo positivas, un poco, de motivación…
P21: Hombre, influir, volvemos a lo mismo, esto es recíproco, lo mismo que yo me retroalimento de la actitud de los alumnos, los alumnos tienen que tener en el proceso una actitud positiva, dinamizadora… Que no quiero decir que esto sea una regla matemática que la aplicas y te da un resultado, porque, además, volvemos a lo de que el alumnado es muy heterogéneo…lo que me quejo muchas veces del alumnado, porque claro, yo hablo, una actitud positiva, yo doy clases en una facultad de periodismo, eso quiere decir que yo tengo muy clara la función del periodista, la función social del periodismo… Y yo me encuentro con alumnos que, serán muy buena gente, pero están totalmente equivocados. Han venido a la facultad con una idea del periodismo, que si no la cambian…estamos hablando de alumnos que estudian periodismo y no ven un solo informativo ni leen un solo periódico en toda la carrera. Entonces, en un momento determinado es imposible conectar con ese tipo de alumnos, y entonces, puede que haya alumnos que lo que para ti es lo motivador, para ellos es “este hombre qué me está hablando”, ¿no? Y, hay veces que es difícil conectar a los alumnos contigo y tú con los alumnos. Pero vamos, creo que sí es fundamental, porque tú eres el dinamizador, con lo cual, el trabajo lo hacen ellos, pero tú eres el que tiene que motivarlos y activarlos. Pero siempre, partiendo de un requisito, que, a veces, yo creo que también, en determinados sectores de alumnos no se da, que es el interés previo. Cuando te encuentras con alumnos que no tienen interés, eso es un…vamos, yo cada vez estoy más convencido de que puedes hacer lo que quieras que no hay nada que hacer, ¿no?


Documento:		4. Ciencias Sociales y Jurídicas\P22 CCSS Creencias
Peso:	0
Posición:	32 - 35
Código:	1. Creencias\Rol docente y actitudes\1.5. Actitudes
E: Siempre. Y, ¿cómo cree que influye su actitud o características en el aprendizaje del alumnado?
P22: De manera general estamos hablando, ¿no? Ya no tiene que ver esto con…
E: No, no, de manera general.
P22: Pues, no lo sé, yo creo que no debe ser un problema, pero, ciertamente, a ellos parece que les gusta los profesores que les hacen reír un poco, que son más simpáticos… Yo, como nunca lo he sido, pues, posiblemente sea un…que exista un poco de barrera con respecto a eso, ¿no?, pero yo te digo, siempre es al principio, porque luego voy estando bastante bien con ellos, en general.


Documento:		4. Ciencias Sociales y Jurídicas\P23 CCSS Creencias
Peso:	0
Posición:	40 - 41
Código:	1. Creencias\Rol docente y actitudes\1.5. Actitudes
E: Seguramente que la vean cercana. ¿Cómo crees que influye tu actitud o características en el aprendizaje del alumnado?
P23: Pues mira, por un lado, creo que bien porque me lo han comentado, que les doy como mucha energía, muchas ganas de aprender, les hago que amen la asignatura y que se interesen... Pero por otro lado soy poco exigente. Entonces, creo que tengo que ser más exigente. Estoy en ese reto. En ese reto de ser más exigente con ellos y más durilla con ellos.


Documento:		4. Ciencias Sociales y Jurídicas\P25 CCSS Creencias
Peso:	0
Posición:	48 - 49
Código:	1. Creencias\Rol docente y actitudes\1.5. Actitudes
E: ¿Cómo crees que influyen esas características de las que me has hablado, la actitud que tienes con los alumnos en el aprendizaje del alumnado? ¿Crees que tienen una influencia en sus creencias, en su aprendizaje, en su enganche a la asignatura y demás?
P25: Esto es complicado ¿eh? Yo he tenido experiencias desde hace mucho tiempo con los alumnos y yo lo que voy observando en los años, es que cada vez, ellos tienen más dificultades. Los alumnos vienen a clase con muy poca base en muchas cuestiones, que se supone que las tienen que tener claras, porque yo doy temas que son recordatorios de bachillerato, de historia contemporánea y cosas así, y no traen nada claro, ellos traen la mente en blanco, no han ordenado interiormente, y entonces, yo me encuentro con mucha dificultad en ese sentido. A mí, los alumnos…y luego hay mucho absentismo, muchísimo. Yo tengo cincuenta y tantos alumnos en clase y luego, me vienen unos 30, pero en comparación con otras compañeras me puedo dar como satisfecha, porque en sus clases hay aún menos estudiantes. Entonces, yo creo que los atrapa el orden y la sistemática de las clases, porque ellos saben por dónde tienen que ir, con otras asignaturas me expresan “es que estamos perdidos”. Pues claro, en otras asignaturas a lo mejor, los dejan más a sus bolas y búscate la vida y sé más independiente. Yo a lo mejor, los atrapo más porque soy más controladora. Pero claro, ellos necesitan eso a veces, ellos necesitan saber por dónde van, qué se tienen que estudiar, qué tienen que hacer; Entonces, yo creo que eso les aporto yo y que le da esa seguridad. Y todo esto, les atrapa, para trabajar la asignatura, para meterse, para motivarse, pero les cuesta mucho, ¿eh?, yo creo que les cuesta mucho trabajo.
