# Supplementary material for: Is pedagogical training an essential requirement for inclusive education? The case of faculty members in the area of Social and Legal Sciences in Spain
Source: PLoS One. 2021 Jul 2;16(7):e0254250. doi: 10.1371/journal.pone.0254250 (PMC8253417; doi:10.1371/journal.pone.0254250)
Supplement: S1 File — (ZIP) [file pone.0254250.s001.zip › 1.6. PROFESOR INCLUSIVO IDEAL (1).rtf]

Documento:		4. Ciencias Sociales y Jurídicas\P1 CCSS Creencias
Peso:	0
Posición:	30 - 31
Código:	1. Creencias\Rol docente y actitudes\1.6. Profesor inclusivo ideal
E: Vale. Y cuáles crees que son las características personales y profesionales que debe tener un profesor ideal de la educación inclusiva.
P1: Pues mira, tiene que ser docente, es decir, tiene que preocuparse porque el alumnado asimile toda la información y competencias que estamos aprendiendo, tanto dentro como fuera de clase. Y eso te exige a ir más allá de la clase, porque tienes que confirmar que lo que tú estás impartiendo, tu asignatura, tus conocimientos, están siendo asimilados correctamente por las personas que tienes delante, tengan algún tipo de discapacidad o no. Y preocuparte de que las condiciones a las que se enfrentan sean las óptimas para poder sacar el máximo provecho a esa inversión de conocimiento. Por eso te digo que las personas con discapacidad, lo importante para ellas, mi experiencia ha sido que lo que quieren es ponerse en las mismas condiciones. Entonces, tú tienes que trabajar para eso, pero la exigencia y los contenidos es igual para todos.


Documento:		4. Ciencias Sociales y Jurídicas\P4 CCSS Creencias
Peso:	0
Posición:	36 - 37
Código:	1. Creencias\Rol docente y actitudes\1.6. Profesor inclusivo ideal
E: Y cuáles crees que son las características personales, profesionales y las actitudes fundamentales que debe de tener un profesor, así como ideal, de la educación inclusiva.
P4: Debe ser una persona con valores, con sentido de la justicia, empática, dispuesta a ayudar y hacer bien su trabajo…no tanto…quizá estoy yéndome un poco como si fuera un favor, no quiero transmitir eso, no sé si puede entenderse así, es una parte de hacer bien tu trabajo, igual que atiendes a otros estudiantes, atender a los estudiantes con discapacidad y hacer todo lo posible para que estén en las mismas condiciones que el resto de estudiantes, pero como una parte del trabajo, no como un favor.


Documento:		4. Ciencias Sociales y Jurídicas\P5 CSS Creencias
Peso:	0
Posición:	42 - 43
Código:	1. Creencias\Rol docente y actitudes\1.6. Profesor inclusivo ideal
E: Y cuáles crees que son las características personales, profesionales y actitudes fundamentales que debe de tener el profesorado para desarrollar una educación inclusiva y dar una respuesta educativa de calidad.
P5: Hombre, sensibilidad, primero sensibilidad, e imagino que luego, instrumentos pedagógicos. Es verdad que yo he hecho algunos cursos en relación de metodologías de la educación, ¿no? Dinámicas de grupo… Que es verdad que facilita algo a lo que tradicionalmente…y ni siquiera se exige para ser profesor. Yo llevo aquí diez años dando clase y yo no he hecho ninguna prueba al respecto sobre cómo… Pero…


Documento:		4. Ciencias Sociales y Jurídicas\P6 CCSS Creencias
Peso:	0
Posición:	24 - 25
Código:	1. Creencias\Rol docente y actitudes\1.6. Profesor inclusivo ideal
E: Y, qué características personales, profesionales y actitudes fundamentales crees que debería de tener el profesorado para dar una respuesta educativa de calidad a todo el alumnado, con o sin discapacidad.
P6: Hombre, yo vuelvo a repetir, creo que es importante la cercanía, creo que es importante humanizar nuestro trabajo que, desde tiempos ancestrales está un poco mecanizado, como todo en la vida, y creo que eso es un error en cualquier tipo de trabajo que nos encontremos y más si cabe en un trabajo en el que, al final, estás educando y estás enseñando algo. No creo que solo nuestra labor sea enseñar la parte de nuestra asignatura, ¿no? Sino que tenemos además una serie de valores que, con nuestro comportamiento diario, seguramente alguien se fije en él y aprenda algo. Entonces claro, de eso no debemos olvidarnos en nuestro día a día como docentes y lo tenemos que tener muy presente desde que entramos en clase hasta que nos vamos, a la hora de responder los mails, a la hora de encontrártelos en cafetería, a la hora de que les surja una duda, a la hora… No sé. Y muchas veces también…yo no llevo muchos años como docente, pero no quiero que nunca se me olvide tener la capacidad de ponerme en el lugar del otro, creo que es muy importante.


Documento:		4. Ciencias Sociales y Jurídicas\P7 CCSS Creencias
Peso:	0
Posición:	36 - 39
Código:	1. Creencias\Rol docente y actitudes\1.6. Profesor inclusivo ideal
E: Entonces, ¿cuáles son las buenas prácticas que debe tener un profesor que tiene y que quiere ser inclusivo? Que quiere atender en igualdad de oportunidades a todo el alumnado que quiere fomentar el aprendizaje y la participación de todos sus alumnos…
P7: Pues, lo primero como persona, yo creo que tiene que tener esa motivación especial y ver en la otra persona, tener empatía hacia la otra persona y, al final, querer a la otra persona, tenerlo como objetivo el que esa persona salga adelante, el no cometer tampoco injusticias, al margen de que esa persona pueda tener necesidades especiales, pero tampoco ser excesivamente duro. Sí que es verdad que tienes que ser diferencial, porque esa persona necesita, no le puedes tratar de la misma forma que a los otros, le tienes que dar también otras oportunidades… eso es igualar, pero que tampoco sea de ninguna forma injusto. Que esa persona vea que le cuesta lo mismo que a los otros, lo que pasa que tú también le das muchas facilidades. Eso también le va a dar a él mucho más refuerzo personal, que ve que también le estás exigiendo lo mismo que a los demás, lo que pasa que le ofreces más oportunidades o más…
E: Más apoyos…
P7: Eso, el tener un seguimiento continuo de esa persona, el que te vean no solo como profesor, sino como profesor tutor también, que les estás orientando en otras cosas que no…como puede ser el motivarle, ya no solo en otras asignaturas sino en otros aspectos, el mostrarte accesible si tiene cualquier dificultad, pues que noten el apoyo ese, yo creo que… Y no sé que más cosas decir, una metodología.


Documento:		4. Ciencias Sociales y Jurídicas\P7 CCSS Creencias
Peso:	0
Posición:	63 - 63
Código:	1. Creencias\Rol docente y actitudes\1.6. Profesor inclusivo ideal
Yo creo que el profesor debe mostrar y formar en valores y muchas veces yo creo que en eso fallamos en la universidad española. Yo creo que tenemos que hacer mucho esfuerzo y los profesores no establecer tanta distancia con el alumno. Sí que es diferente el profesor…lo que no se puede es endiosar y poner dificultades para que el alumno acuda. Si todos recordamos, yo estudié ingeniería y no recuerdo nunca haber ido a tutorías porque teníamos miedo al profesor. Eso no puede ser. Impide el aprendizaje. Al hacerlo, al final sales de la universidad con un rechazo. Yo de hecho, un rechazo me dirigió completamente a otra…al margen de que, por temas de empleo, dejó de gustarme mi carrera. Y yo creo que eso ocurre mucho en España, que desgraciadamente la enseñanza es una enseñanza con profesores que no están por vocación ahí, que no tienen una formación especial o un poco de empatía hacia el alumno…


Documento:		4. Ciencias Sociales y Jurídicas\P8 CSS Diseños
Peso:	0
Posición:	14 - 15
Código:	1. Creencias\Rol docente y actitudes\1.6. Profesor inclusivo ideal
E: Y cuáles crees que son las características personales, profesionales y actitudes que debería de tener un docente, así como ideal de la educación inclusiva, que atiende a todos y a todas.
P8: Yo creo que tiene que estar motivado con la docencia con independencia del tipo de alumnos, que debe tener cierta información sobre la discapacidad de sus alumnos con minusvalías, si se puede llamar así, porque ahora parece que está mal visto lo de las minusvalías. Entonces, no sé si formarse, pero sí al menos informarse de cómo puede hacer que el seguimiento de la asignatura que tenga que impartir sea más efectivo o por lo menos más positivo para el estudiante. Con esto no se quiere decir que…o sea, no se trata de que pases el nivel de exigencia con ese alumno, sino de que adaptes la forma comunicarte con él, la forma de desarrollar las clases teniendo en cuenta sus limitaciones o sus dificultades. O sea, yo no les aprobaba por ser personas, entre comillas, con dificultades, lo que hacía era adaptarme a sus necesidades. Si necesitaban más tutorías, pues más tutorías, si necesitaban otro tipo de actividad… Entonces, yo creo que es muy, muy positivo un acercamiento personal.


Documento:		4. Ciencias Sociales y Jurídicas\P9 CCSS Creencias
Peso:	0
Posición:	34 - 37
Código:	1. Creencias\Rol docente y actitudes\1.6. Profesor inclusivo ideal
E: ¿Cómo crees que influye tu actitud en el aprendizaje del alumnado?
P9: Pues, yo creo que influye mucho porque es el simple hecho de cómo tú transmites las cosas y si lo transmites como algo positivo, pues ellos lo van a ver así y si lo transmites como algo negativo, pues lo mismo.
E: ¿Cómo crees que son las características personales y profesionales y actitudes fundamentales que debe tener el profesorado que desarrolla una educación y da respuesta de calidad al alumnado con y sin discapacidad?
P9: Pues yo creo que lo fundamental es que te tiene que gustar, o sea, yo creo que es una profesión vocacional, o sea, te tiene que gustar el trato con los alumnos, el discutir con ellos, el que ellos te lleven la contraria. A mí me encanta que ellos me planteen algo diferente a lo que yo estoy contando que es que nosotros no tenemos la verdad absoluta y menos en una asignatura como la que yo doy. Y bueno, hay otras que hay verdades que no se pueden cambiar, pero en las asignaturas que yo doy, tanto en la del comportamiento del consumidor como en la de gestión de marketing, las cosas pueden verse desde muchos puntos de vista. Entonces, a mí me parece que está bien y hay que dejarles y tener la capacidad de permitir que lo digan. Yo creo que es vocacional, que la gente que se dedica a la educación, a cualquier nivel, tiene que ser gente que le gusta de verdad compartir sus conocimientos y no pensar que por saber más somos diferentes de los demás, porque no es verdad que sepamos más, sino que sabemos de otras cosas. Sabemos mucho de algo o un poco más de algo, pero ellos tienen miles de conocimientos que no vamos a tener nosotros nunca en la vida. Entonces, ¿por qué no vamos a aprender nosotros de nuestros estudiantes? Yo aprendo muchísimo de ellos. Entonces, yo creo que una de las cosas tiene que ser que sea vocación y que sea súper abierto a la crítica y al conocimiento que ellos te aportan.


Documento:		4. Ciencias Sociales y Jurídicas\P10 CCSS Creencias
Peso:	0
Posición:	39 - 44
Código:	1. Creencias\Rol docente y actitudes\1.6. Profesor inclusivo ideal
E: Claro. Y, claro, este proyecto va sobre educación inclusiva, sobre inclusión en la universidad, entonces, me gustaría preguntarte que qué características, tanto personales como profesionales, crees que debe de tener el profesorado universitario, en general, ya no solo hablando de ti, sino en general, para que todos los alumnos se puedan sentir incluidos.
P10: Uf, pues no sé, no sé. Sinceramente, no sé, por decir algo, pero, insisto, esto es cuando te preguntan de un tema y te dicen “como eres economista…”, “sí, pero yo de eso no sé”, pues no veas de esto otro, no tengo ni idea, ¿vale? Puestos a eso, pues yo creo que, en general, si tu preocupación, digamos, y tu objetivo, es que los estudiantes aprendan, y poner los máximos medios para que lo hagan, pues, sabiendo, además, que cada uno es de un color, de una pasta y de tal, pues tratar de no sé, de llegar, siendo respetuoso con ellos, cordial, de manera que te sientan más cercano, y procurando, pues no sé, que las cosas más complejas o más abstractas, puedan tender puentes para que puedan llegar desde diferentes posiciones…no sé eso qué es, pero pienso que puede tener alguna lógica. Hay gente de todos los colores y tal, por lo que cuentan y tal. Entonces, no sé, eso es lo que veo. Tampoco creo que sea una cosa que exija tantísimo tiempo y esfuerzo, a lo mejor, también es una cuestión de carácter y de forma de ser.
E: De actitud, ¿no?
P10: De actitud. Por ejemplo, un vecino de mi escalera, del que la gente decía, en plan de broma, que debía ser sordomudo, porque el hombre, te lo encontrabas en el ascensor y no decía nada. Entonces, bueno, me dediqué cada vez que me cruzaba con él a saludarlo y darle los “buenos días”. Al final el hombre no sólo me saludaba, sino que incluso me sonreía e intercambiaba alguna frase conmigo. Cada uno es como es, está claro, porque a lo mejor no es el más dicharachero del mundo, pero supongo que si tú no le dices nada, pues adiós, muy buenas. Entonces, cómo se llama eso, no tengo ni idea.
E: Vale, pero te he entendido. 
P10: Vale.


Documento:		4. Ciencias Sociales y Jurídicas\P11 CCSS Creencias
Peso:	0
Posición:	39 - 40
Código:	1. Creencias\Rol docente y actitudes\1.6. Profesor inclusivo ideal
E: Claro. Y cuáles crees que son las características personales, profesionales y actitudes fundamentales que debería de tener un profesor así como ideal de la educación inclusiva.
P11: Pues la primera la empatía, conocer a la otra persona cómo es y cómo se siente. Entonces, luego es más fácil tratar si te pones en el lugar de esa persona. Lo segundo, por supuesto, la vocación docente, o sea, que tú no estás para transmitir una materia, sino para que el que sea, aprenda contigo, es decir, pasión por el aprendizaje. Y tercero y para mí muy importante, que a veces no se da siempre, es el respeto, que hay que trabajarlo, los alumnos necesitan tener respeto por parte de sus profesores y los profesores necesitan ganarse el respeto de sus alumnos. Entonces, yo esto lo cuido mucho, es decir, a los alumnos no les pongo el listón alto, yo soy muy accesible, pero claro, tú ya sabes que a los chavales ahora de 20 años, les das así y te cogen así. Entonces, también hay que marcar unas líneas, digamos, que hay un espacio común, un intervalo que hay que crear. Para mí es muy importante, es decir, “de aquí a aquí, podemos jugar, podemos reír y somos compañeros de esta asignatura, yo soy el profe o el entrenador, pero estamos en el mismo equipo. De aquí para allá te estoy faltando el respeto, de aquí para allá me estás faltando el respeto tú. O sea, hay líneas rojas que no podemos cruzar, pero hay que establecerlas, para mí eso es importante, establecerlas”. 


Documento:		4. Ciencias Sociales y Jurídicas\P12 CCSS Creencias
Peso:	0
Posición:	40 - 41
Código:	1. Creencias\Rol docente y actitudes\1.6. Profesor inclusivo ideal
E: ¿Cuáles crees que son las características personales, profesionales actitudes que tiene que tener un profesorado que desarrolla educación inclusiva y que da una respuesta de calidad al alumnado con y sin discapacidad? 
P12: Yo es que creo que son las características que debería tener todo profesor universitario y es tener una mente abierta, nada más. Esta mente abierta, pues te permite adaptarte y buscar alternativas, pues para diferentes situaciones que se plantea, es decir si en la clase se te planeta un conflicto entre dos alumnos, pues tienes que tener la mente lo suficientemente abierta para solucionarlo. Es una amplitud de miras, no centrarte en los problemas, sino buscar soluciones y estas soluciones vienen del diálogo.


Documento:		4. Ciencias Sociales y Jurídicas\P13 CCSS Creencias
Peso:	0
Posición:	31 - 32
Código:	1. Creencias\Rol docente y actitudes\1.6. Profesor inclusivo ideal
E: ¿Y tú qué características crees que tiene que tener el profesorado que desarrolla una educación inclusiva?
P13: Tiene que ser empático, saber ponerse en el lugar del otro, fundamental.


Documento:		4. Ciencias Sociales y Jurídicas\P14 CCSS Creencias
Peso:	0
Posición:	32 - 33
Código:	1. Creencias\Rol docente y actitudes\1.6. Profesor inclusivo ideal
E: Y, en términos ya más generales, cuáles crees que son las características, ya no solo profesionales, sino también personales, que debe tener cualquier profesor para que el alumnado con discapacidad se pueda sentir incluido en el aula.
P14: Pues, supongo que intentar generar un ambiente de participación en clase, ¿no? Pues hacer preguntas, que se sientan partícipes del proceso, salir a la pizarra y que traten de resolver algo, de una pregunta que entre todos saquen adelante el proceso de enseñanza-aprendizaje, ¿no? Que no sea solo mi trabajo.


Documento:		4. Ciencias Sociales y Jurídicas\P15 CCSS Creencias
Peso:	0
Posición:	42 - 43
Código:	1. Creencias\Rol docente y actitudes\1.6. Profesor inclusivo ideal
E: Vale. Y cuáles crees que son las características personales y profesionales que debería de tener un profesor o profesora, así como ideal de la educación inclusiva, es decir, que da una respuesta de calidad a todos y a todas sus estudiantes.
P15: Bueno, pues primero, que sepa de lo que habla y, en segundo lugar, que lo sepa transmitir. Y, para ser un buen profesor hay muchas maneras diferentes. Depende del tamaño del aula, no es lo mismo tener 15 que 100 alumnos. No es lo mismo una asignatura que otra… Entonces, hay que adaptarse a lo que hay, a lo que tienes. 


Documento:		4. Ciencias Sociales y Jurídicas\P17 CCSS Creencias
Peso:	0
Posición:	84 - 89
Código:	1. Creencias\Rol docente y actitudes\1.6. Profesor inclusivo ideal
E: Y, hablando así…porque estamos hablando de rol docente. Desde tu punto de vista, por tu experiencia, porque tú has sido alumno, por tus ideas en general de lo que es un buen docente, ¿qué características personales y profesionales crees que debe tener un profesor o profesora que tiene en cuenta a todo el alumnado en su aula? Que no excluya, vamos que hace que todo el mundo participe y vaya aprendiendo. Tú qué características personales y profesionales crees que debería tener.
P17: Como personal, tiene que tener una cierta resistencia a la frustración, porque te encuentras cada cosa que te caes de espaldas. Yo, ahora mismo, por ejemplo, ahora les he pedido que me hagan una ficha y me han hecho la ficha que les ha dado la gana, se la tengo que devolver, la he puesto en la página web y no ha habido forma. El año pasado no hubo forma, y nada más que les he pedido una foto, y no hubo forma. Te encuentras con mucha gente que no sabes cómo han llegado a este nivel, no entiendo cómo han aprobado los cursos previos y estoy dando clases, teóricamente, en una carrera fuerte. Cuando daba clase en otras carreras más suaves, era terrible, de tener que explicar cosas de matemáticas básicas, o sea que, muy mal, muy mal. Y entonces, tienes que tener una cierta resistencia a la frustración, a saber, que te encuentras con casos así, con casos de exámenes que no eres capaz de descifrar la letra, con alumnos que, además, no quieren estudiar, porque hay muchos niños que no quieren estudiar, que están porque bueno, me han mandado a estudiar, y he cogido esto porque he podido entrar y no quieren estudiar o no quieren estar en la carrera que ellos quisieran estudiar. Entonces, principalmente eso, principalmente, resistencia a la frustración, porque te encuentras con muchos obstáculos contra los alumnos, frente a los alumnos, que tienes que ir superando poco a poco para que se integren y que ellos vean como una cosa interesante la asignatura, que a ellos les preocupe, que quieran aprender.
E: Pero, todo el mundo no está capacitado para ser profesor, ¿no?
P17: No. 
E: Porque claro, tú puedes tener mucha resistencia a la frustración, pero tienes que tener otras cualidades.
P17: A mí es que me gusta, o sea, yo tengo una dedicación…hay que tener cierta dedicación a preparar las clases y a hacer presentaciones agradables, porque ahora trabajamos con presentaciones, y a mí es que me gusta dar las clases. Lo que sí que veo como mal profesor es ese profesor que va a leerme un libro, o que va sin la clase preparada, porque eso lo he visto, lo he vivido y eso me sienta como un tiro. Eso es lo que no debe de hacerse. Después, ¿lo que debe de hacerse? Preparar bien las clases, preparar los materiales, presentar las cosas adecuadamente, tratar con respeto a todos, ser amable, hasta cierto punto, porque llega un punto que, como hoy, que voy a devolver la ficha. El año pasado, por primera vez, tuve que expulsar a dos alumnos de clase, yo lo entiendo, estaban entretenidos con otra cosa, “jijijaja”, yo no les digo que vengan a clase, tienen que venir a las prácticas, pero puedes irte de clase, vete de clase, si no te voy a echar cuentas, me va a ser indiferente que estés o no estés, el que esté, que esté entretenido, que esté viéndome a mí. Además, lo que quiero es que me atiendan a mí, no quiero que miren los libros…quiero que me atiendan a mí la clase que estoy dando y después que cojan lo que quieran.


Documento:		4. Ciencias Sociales y Jurídicas\P18 CCSS Creencias
Peso:	0
Posición:	48 - 49
Código:	1. Creencias\Rol docente y actitudes\1.6. Profesor inclusivo ideal
E: Y luego, ¿me podrías decir algunas características…? Parece que te estoy… ¿no? Pero la entrevista se trata de eso, de que saquemos lo mejor de ti, para conocerte como docente, ¿no? Desde tu punto de vista, algunas claves que deben tener los docentes que hacen partícipes a todo el alumnado, que son inclusivos, desde tu punto de vista, o cuando tú has tenido profesorado, aquellos que te han marcado o que te han gustado, cuáles son las claves o pinceladas que tienen que les hacen que aprenda todo el alumnado, digamos, profesores carismáticos que hayas conocido en tu trayectoria o tu experiencia.
P18: Hombre, no sé, en derecho, al menos…es que depende también del tipo de curso en el que imparta, ¿no? Pero también muy relacionado con las asignaturas, entonces, profesores carismáticos, sobre todo, yo recuerdo las asignaturas prácticas, para mí, derecho del trabajo, que, cuando yo estudiaba, era una asignatura que me encantaba, entonces, claro, cuando una asignatura te gusta muchísimo, vas, aparte de todo, que también depende de si es muy práctica o no. Entonces, yo recuerdo, cuando son asignaturas y clases muy teóricas, pero, sobre todo, que se utiliza un lenguaje…porque yo en cuarto y en quinto de derecho podías utilizar un determinado tipo de lenguaje, pero depende del curso en el que estés tienes que utilizar, si quieres conectar con los alumnos, enseñarlos a utilizar un lenguaje, en este caso, jurídico, pero no darle una clase continuamente utilizando un tipo de lenguaje que ellos no…que ellos pueden desconectar. Entonces, aquellos que te acercan más esa asignatura al día a día y que te hacen ver lo importante de la asignatura o cuáles son las…creo yo que tienen muchas más posibilidades de tener un desarrollo óptimo de la docencia o, al menos, yo recuerdo eso, que, a mí, los que me gustaban eran impuestos, la parte de los impuestos, porque era muy práctico, y la parte del derecho del trabajo, que era muy, muy práctico. Me gusta también el constitucional, por ejemplo, ¿no? Pero, constitucional se daba en primero y estas personas para hacértelo más…tenían que utilizar un lenguaje mucho más asequible, aunque ya, al final, se utilizase, pero sí para llevarte a tu terreno tienes tú también que bajar un poco y, a veces no es divagar sobre un determinado tema, sino, a ver, preparártelo, utilizar palabras…de vez en cuando vocabulario jurídico, “este término, qué significa”, “bueno, pues en este contexto significaría esto”, pero, al menos, yo no soy de las que opinan que una clase teórica, magistral, implique utilizar muchos términos y dejarte a la gente olvidada, que no te siga.


Documento:		4. Ciencias Sociales y Jurídicas\P18 CCSS Creencias
Peso:	0
Posición:	50 - 53
Código:	1. Creencias\Rol docente y actitudes\1.6. Profesor inclusivo ideal
E: Vamos a pasar ahora a la parte de formación, si crees que el profesorado es necesario que se forme para un mejor proceso de aprendizaje.
P18: Sí. Yo he hecho muchos cursos. Ya, últimamente hago menos, y, además, he hecho muchos proyectos de innovación docente, tenemos. Y esos proyectos son dirigidos a la formación de los alumnos, para facilitarles la formación, que este año he leído que sí que nos lo habían aceptado, es de derecho sindical, aprender sobre derecho sindical, pero con pequeños casos prácticos, pero cortos, cortitos, y como en derecho sindical son siete temas y cada uno de un contenido distinto, entonces, es una de las formas de verlo.
E: Y qué formación consideras que debería tener un profesor, así imprescindible. No la que tú tengas, sino la que debería de tener porque puede influir en que el alumno aprenda más. El alumno con discapacidad o sin discapacidad.
P18: Hombre, yo ya te digo, la formación es simplemente, sobre todo, captar…yo es que no sé qué formación podría ser, pero sí enseñar, al menos, que los profesores sepan captar la atención del alumno. Entonces, ¿que lo sepa captar? Hay veces que sí y hay veces que no. Hay veces que me he llevado una hora y media ahí dando clase y, entonces, la atención que tienen al principio no es la misma que la que tienen al final, por mucho que yo les haya dicho, incluso “esto es muy importante, esto puede caer en el examen”, cuando yo los veo un poco perdidos, sí es cierto que “esto es muy importante para la práctica, esto es…”. Entonces, ellos directamente vuelven a…yo creo que sí deberíamos de tener una cierta formación sobre cómo captar la atención. De eso hay cursos también, lo que pasa que este año no me he apuntado, este año no me apunto a nada, ya con el proyecto tengo suficiente, pero sí es verdad que hay cursos de cómo captar la atención de los alumnos, no en las clases prácticas que ellos siempre están dispuestos, sino en las más complejas.


Documento:		4. Ciencias Sociales y Jurídicas\P20 CCSS Creencias
Peso:	0
Posición:	36 - 37
Código:	1. Creencias\Rol docente y actitudes\1.6. Profesor inclusivo ideal
E: Claro. Y qué características personales, profesionales y actitudes crees que debería tener un profesorado así ideal de la educación inclusiva, que da respuesta de calidad a todos y a todas, qué no le podría faltar.
P20: Mira, yo creo que profesionales, yo creo que todos lo tenemos, o sea que, por ahí yo no creo que vaya a reforzarse en nada, pero, sin embargo, sí creo que hay que tener una cierta actitud personal libre de todo tipo de problemática o de prejuicio sobre temas específicos y demás. En la facultad, yo no lo he tenido como alumno, pero ha habido también una persona en silla de ruedas, pues hemos tenido que ir cambiando las clases, porque me correspondió ser durante una época vicedecana, entonces tuvimos que cambiar las clases según esa persona se iba incorporando a los cursos superiores porque bueno, nuestro centro data del siglo XV, entonces, claro, tenemos que ir adoptando medidas, pero bueno, es que eso tiene que ser así.


Documento:		4. Ciencias Sociales y Jurídicas\P21 CCSS Creencias
Peso:	0
Posición:	56 - 59
Código:	1. Creencias\Rol docente y actitudes\1.6. Profesor inclusivo ideal
E: Estupendo. Y, ¿cuáles crees que son las características y actitudes personales y profesionales que debe tener el profesorado que desarrolla una educación inclusiva y da una respuesta de calidad a todo el alumnado, independientemente de que tenga o no tenga discapacidad?
P21: Bueno, está un poco en la línea de lo que estábamos hablando ahora mismo, o sea, para mí, sobre todo, hay una cuestión que me parece fundamental, que es la empatía. De hecho, mis peores crisis docentes son cuando empiezo a bajar y en periodos, afortunadamente reducidos, casi a perder la empatía del alumnado, ¿no? Cuando te desesperas, ¿no? Tú me entiendes, las actitudes del desinterés o del desencanto, porque lo que estamos sufriendo hoy en la universidad, y, concretamente en esta facultad, es que tenemos un alumnado muy desencantado de saber que la profesión está fatal, que están estudiando algo que son conscientes de que no tiene prácticamente futuro porque en la crisis los medios y la profesión es muy…es vital, ¿no? Y, en ese sentido, pero tú tienes que empatizar con el alumnado, y ahí es donde volvemos a lo que decíamos antes, empatizar es ponerte en el lugar del otro, cuando te encuentras con un alumno con una determinada discapacidad, te tienes que poner en el lugar y punto, y a partir de ahí…pero si no tienes esa capacidad, yo siempre, cuando he analizado problemas en otros docentes que conozco, compañeros y tal, cuando veo que hay problemas que son difíciles de solucionar y cuando veo, que es lo peor que puede pasar, que es cuando hay que retirarse…yo siempre digo que la docencia hay que retirarse cuando uno pierde completamente la capacidad de empatizar con los alumnos, de ponerse en el lugar de los alumnos, ¿no? 
E: Totalmente.
P21: Partiendo de esa característica, lo demás son ya cuestiones muy puntuales que van a depender de las circunstancias, de la asignatura, de los créditos, del tipo de prácticas… Pero siempre partiendo de ese principio general, ¿no? De que tienes que entender y empatizar con los alumnos.


Documento:		4. Ciencias Sociales y Jurídicas\P22 CCSS Creencias
Peso:	0
Posición:	36 - 39
Código:	1. Creencias\Rol docente y actitudes\1.6. Profesor inclusivo ideal
E: Y, cuáles crees que son las características personales y profesionales, así como las actitudes fundamentales, que debe tener el profesorado que desarrolla una educación inclusiva en la universidad, para dar una respuesta de calidad al alumnado que tiene discapacidad y que no tiene discapacidad.
P22: ¿Características del profesorado?
E: Sí, profesionales, personales y actitudes.
P22: Hombre, en principio, yo creo que, una capacidad de empatizar con la gente, sensibilidad para entender los problemas de los alumnos, en concreto, hablo de los alumnos con discapacidad ¿Con respecto a los otros? Yo creo que nada especial, simplemente, que te guste estar con alumnos, te guste enseñar, y te guste tu asignatura, que tengas ganas de comunicarles. Eso es algo complicado con los alumnos de ahora, ¿eh? Porque no son receptivos, y eso hace que no te motives tú y es como una rueda, ¿no? Tú no les motivas a ellos y ellos… Entonces, eso sí que lo veo ahora con los alumnos. Con los alumnos que tienen discapacidad, pues yo creo que sí, sensibilidad, empatía, buena comunicación…


Documento:		4. Ciencias Sociales y Jurídicas\P23 CCSS Creencias
Peso:	0
Posición:	42 - 43
Código:	1. Creencias\Rol docente y actitudes\1.6. Profesor inclusivo ideal
E: Y, ¿cuáles crees que son las características personales o profesionales, así como actitudes fundamentales, que debe tener el profesorado que desarrolla educación inclusiva y que da una respuesta al alumnado, con o sin discapacidad?
P23: Pues, fíjate que creo que tiene que tener flexibilidad porque hay profesores en la universidad que tienen la cabeza muy “cuadrada” con que “esto es lo que se tiene que aprender en mi asignatura” y no entienden razones o circunstancias personales. Y yo creo que para hacer educación inclusiva tienes que tener cierta flexibilidad y entender que en la vida el libro de texto no es lo más importante. O sea, que hay cosas que... Valores o actitudes, habilidades o competencias que no se adquieren con un libro de texto.


Documento:		4. Ciencias Sociales y Jurídicas\P25 CCSS Creencias
Peso:	0
Posición:	50 - 51
Código:	1. Creencias\Rol docente y actitudes\1.6. Profesor inclusivo ideal
E: Eso es positivo también, teniendo en cuenta que son de primer año. Entonces, ese salto, digamos que tu estrategias pues lo suavizan un poco. Muy bien. Ahora de manera general, ¿cuáles crees que son las características tanto personales como profesionales y actitudes que crees que tiene que tener un buen profesor para que desarrolle una educación de calidad, que atienda a todo su alumnado, etc.?
P25: Pues bueno, un buen profesor para mí, tiene que ser una persona honesta, tiene que formarse, y saber muy bien discriminar qué se le dice al alumnado, ya que no todo hay que decirlo. Yo creo que no es un buen profesor aquel que da un rollo y se queda tan satisfecho y no capta al alumnado, no sabe qué es lo que el alumnado ha aprendido. Me parece, que un buen profesor tiene que ser dinámico, divertido, creativo, tiene que hacer propuestas interesantes al alumnado para que estos se motiven y se entusiasme.  
