# Supplementary material for: Is pedagogical training an essential requirement for inclusive education? The case of faculty members in the area of Social and Legal Sciences in Spain
Source: PLoS One. 2021 Jul 2;16(7):e0254250. doi: 10.1371/journal.pone.0254250 (PMC8253417; doi:10.1371/journal.pone.0254250)
Supplement: S1 File — (ZIP) [file pone.0254250.s001.zip › 1.9. CLAVES PARA EL APRENDIZAJE.rtf]

Documento:		4. Ciencias Sociales y Jurídicas\P1 CCSS Creencias
Peso:	0
Posición:	23 - 23
Código:	1. Creencias\Proceso de enseñanza y aprendizaje\1.9. Claves para el aprendizaje
Pero luego yo sé que hay otro tipo de discapacidades que no se ven, entonces, por eso es muy importante que el primer día de clase todo el alumnado sepa que tú estás dispuesto a escuchar y a ayudar. Y que el alumno pueda expresarse en público si lo desea o en privado sobre su circunstancia y ver en qué medida el profesor y la asignatura se pueden adaptar a esas circunstancias. Eso es fundamental para mí.


Documento:		4. Ciencias Sociales y Jurídicas\P1 CCSS Creencias
Peso:	0
Posición:	32 - 33
Código:	1. Creencias\Proceso de enseñanza y aprendizaje\1.9. Claves para el aprendizaje
E: Vale. Y, por último, en este apartado, cómo crees que influye tu rol como docente en el aprendizaje del alumnado.
P1: Sí influye porque un docente, cuando le interesa mucho su asignatura, su campo de conocimiento y se preocupa porque ese conocimiento llegue al alumnado, el alumnado lo asimile y lo trabaje… Yo intento, y hablo de mi experiencia particular, tengan discapacidad o no, eso ya me da un poco igual, que participe activamente en la generación de ese conocimiento. Que ese conocimiento le sirva para algo, que vayamos más allá de los contenidos teóricos, sino que introduzcamos contenidos prácticos, que ellos vean que lo que están aprendiendo aquí les sirve para algo… Y al mismo tiempo, para mí es muy importante trabajar las competencias verticales, es decir, el conocimiento, sino también las transversales, que van a influir en su inserción laboral: comunicación, expresión, dominio de idiomas extranjeros… Vemos vídeos en inglés en clase… Hacemos muchas cosas que, en principio, parece que “uh, esto no sé a qué viene”, pero que a la hora de desarrollar determinadas competencias laborales o profesionales les va a venir bien. Es verdad que en una asignatura tú puedes hacer cosas limitadas, pero cada granito de arena suma. Entonces, para mí eso es muy importante a la hora de diseñar mis clases. Y los alumnos están super contentos, eso se ve en las valoraciones. O sea, yo disfruto en clase, ellos disfrutan, aprendemos, sacan buenas notas, y además aprenden cosas que les van a servir para su futuro profesional, con lo que todos ganamos.


Documento:		4. Ciencias Sociales y Jurídicas\P1 CCSS Creencias
Peso:	0
Posición:	40 - 41
Código:	1. Creencias\Proceso de enseñanza y aprendizaje\1.9. Claves para el aprendizaje
E: Claro. Y qué crees tú que es lo más importante para que los estudiantes aprendan adecuadamente.
P1: Para mí es fundamental, y es lo que yo utilizo, que ellos participen en todo el proceso, desde la construcción de los contenidos, el desarrollo y la evaluación también. Nosotros cuando hacemos trabajos grupales, se autoevalúan entre ellos, se dicen qué cosas hay que mejorar, yo hago como unos jurados que les dicen a sus propios compañeros qué pueden mejorar… Entonces, eso genera una dinámica en la que constantemente estamos aprendiendo, ya sea de economía o de cómo hacer un trabajo en equipo, o de cómo hablar en público de un tema… Cosas que luego le van a servir para su desarrollo profesional, ¿no? Entonces, yo les intento involucrar en todo el proceso, desde la creación de contenidos, al desarrollo de contenidos, asimilación y evaluación.


Documento:		4. Ciencias Sociales y Jurídicas\P2 CCSS Creencias
Peso:	0
Posición:	46 - 47
Código:	1. Creencias\Proceso de enseñanza y aprendizaje\1.9. Claves para el aprendizaje
E: Y más sobre el proceso de aprendizaje, ¿tú qué crees que es lo que más importante para que los estudiantes aprendan?
P2: Pues para aprender, lo que hay que tener en primer lugar son ganas de aprender. Nos encontramos con mucha gente que no tienen esa motivación, pero a veces conseguimos despertarla, a veces se puede conseguir despertar esa motivación. Para ellos tienes que intentar hablar en su lenguaje, en su nomenclatura, usando las herramientas a las que ellos están habituados, con las nuevas tecnologías y tal, pero intentando que las usen bien, ¿no? Las referencias correctas y tal. Entonces, yo creo que para motivarlos hay que conseguir que le vean la utilidad a todo lo que están trabajando, a todo lo que están haciendo. O sea, que a todo le vean una proyección en la práctica. Es que eso hoy en día a la gente le interesa muy poco, piensan que todo lo tienen en Google, tienen que ver que toda esa información tiene una utilidad directa e inmediata en su entorno, entonces, todos los ejemplos son fundamentales para motivar a los alumnos. 


Documento:		4. Ciencias Sociales y Jurídicas\P2 CSS Diseños
Peso:	0
Posición:	18 - 19
Código:	1. Creencias\Proceso de enseñanza y aprendizaje\1.9. Claves para el aprendizaje
E: Vale, vale. ¿Tú cuáles crees que son las principales dificultades que tienen los estudiantes para superar tu asignatura?
P2: Una nada más, venir a clase, que hay muchos que pasan de venir a clase, y estudiar un poquito. Los que estudian y los que vienen a clase no tienen dificultades. Yo no puedo identificar una cuestión que diga “pues por esto me suspende la gente”, siempre es por eso, o porque no vienen o porque no estudian, nada más.


Documento:		4. Ciencias Sociales y Jurídicas\P2 CSS Diseños
Peso:	0
Posición:	28 - 29
Código:	1. Creencias\Proceso de enseñanza y aprendizaje\1.9. Claves para el aprendizaje
E: Pero con esto que estás comentando con respecto a lo qué hacer, tú cómo lo facilitas, en referencia a lo que haces, la metodología, la evaluación…
P2: Pues siempre con respecto a las necesidades específicas, claro. Cuando he conocido determinadas circunstancias pues, o bien he adaptado todos los ítems que has mencionado o bien alguno en concreto claro. Es que depende de cada caso, pero vuelvo a repetir que la flexibilidad y la tutoría es fundamental.


Documento:		4. Ciencias Sociales y Jurídicas\P3 CSS Creencias
Peso:	0
Posición:	40 - 41
Código:	1. Creencias\Proceso de enseñanza y aprendizaje\1.9. Claves para el aprendizaje
E: Vale. En relación con el proceso de enseñanza-aprendizaje de los estudiantes, ¿cómo crees tú que aprenden los estudiantes? ¿Qué crees tú que es lo más importante para que los estudiantes puedan aprender?
P3: Que el profesor haga que ellos estén implicados, que no haga que se sientan como diferente, sino que haga que se sientan uno más. Y esto qué significa, pues significa que hay que adoptar ciertas medidas en positivo para estas personas con discapacidad para conseguir que puedan seguir las clases de alguna manera. Así que eso, hacer que todos estén implicados, esto es lo más importante. 


Documento:		4. Ciencias Sociales y Jurídicas\P4 CCSS Creencias
Peso:	0
Posición:	42 - 43
Código:	1. Creencias\Proceso de enseñanza y aprendizaje\1.9. Claves para el aprendizaje
E: Y, qué crees que es lo más importante para que el alumnado aprenda adecuadamente.
P4: Lo más importante para que el alumnado aprenda es que tenga un buen profesor que le haga estar enganchado a la asignatura y eso, que lo enganche, que le siente una base mediante la enseñanza, ¿no? y que luego lo culmine con su propio esfuerzo, pero eso es muy importante.


Documento:		4. Ciencias Sociales y Jurídicas\P5 CSS Creencias
Peso:	0
Posición:	52 - 53
Código:	1. Creencias\Proceso de enseñanza y aprendizaje\1.9. Claves para el aprendizaje
E: Te voy a preguntar ahora por el proceso de enseñanza-aprendizaje. Desde tu punto de vista, qué es lo más importante para que el alumnado pueda aprender adecuadamente.
P5: No sé si es muy básico, pero primero, tener muy claro lo que quieres contarle, ¿no? Y después, yo creo que lo decía un compañero en uno de estos cursos, renovar el compromiso con los alumnos que uno tiene, ¿no? Yo no soy profesor a tiempo completo, no soy profesor, pero es verdad que, lo que te decía antes, yo trato de tomarme en serio mi trabajo, porque tratas con personas y eso es más importante que cuando no lo haces, ¿no? Entonces, ese compromiso de trabajarte bien lo que tienes que impartir, hacerlo de la manera que uno pueda, ¿no? Hay gente que tiene más o menos habilidades de…pero no sé…


Documento:		4. Ciencias Sociales y Jurídicas\P6 CCSS Creencias
Peso:	0
Posición:	32 - 33
Código:	1. Creencias\Proceso de enseñanza y aprendizaje\1.9. Claves para el aprendizaje
E: Vamos a hablar ahora sobre el rol de lo que es enseñar y aprender. Desde tu punto de vista, ¿qué es lo más importante para que los estudiantes puedan aprender adecuadamente?
P6: Hombre, pues yo creo que es importante, primero, que el profesor esté formado de la asignatura que va a impartir, eso es fundamental. Y luego, creo que es importante, como te decía antes, trasladarle las asignaturas en su idioma, o sea, en un idioma…por eso te decía lo de ponerte en el lugar de ellos, no llegar a clase y ponerte a contar todo lo que sabemos nosotros como profesores, que eso a ellos ahora mismo no les ayuda prácticamente en nada. Creo que les ayuda que les hablemos en su idioma, que llevemos la materia a su manera de entender ahora mismo el mundo, la vida, la economía o la materia que estemos impartiendo.


Documento:		4. Ciencias Sociales y Jurídicas\P8 CCSS Creencias
Peso:	0
Posición:	118 - 121
Código:	1. Creencias\Proceso de enseñanza y aprendizaje\1.9. Claves para el aprendizaje
E: Otra vez sentados ahí. Y, desde tu punto de vista, P8, ya que yo creo que tú has reflexionado mucho sobre el aprendizaje, la evaluación y cómo ayudar a los alumnos, ¿qué crees que ayuda más al alumno para aprender adecuadamente? No sé si la metodología, ya que estabas hablando…o el sistema de evaluación que les planteas, no sé.
P8: Vamos a ver, el sistema de evaluación tiene que ser coherente con tu metodología, si no, los haces un lío, los pierdes y, además, son muy hábiles encontrando incoherencias. Pero yo creo que el éxito de verdad del aprendizaje de los alumnos es hacerles entender que no se trata de incorporar ideas para acceder al curso siguiente, como si fuese un disco duro, sino que tienen que intentar buscar siempre eso que tiene que ver con lo que ya saben. Yo siempre les digo “tenéis que buscar en el fondo de vuestro conocimiento. Sabéis mucho más de lo que os pensáis que sabéis, Muchísimo más, pero no os habéis parado nunca a pensarlo ¿Os pensáis que os estoy contando algo nuevo ahora? No os estoy contando nada nuevo ¿En teoría económica habéis visto elasticidades? Pues fijaros lo que es esto que os pongo aquí yo con betasulcero, una elasticidad, porque es cómo crecería una variable cuando actúa otra variable en sentido positivo o negativo. Eso es el efecto respuesta y eso es el concepto de elasticidad y lo habéis visto en micro, en macro durante tres años de la carrera ¿Os habéis planteado alguna vez que esto era…?” Entonces claro, se te quedan mirando como “y ahora fijaros, una elasticidad, ¿qué es? Lo que habéis visto en matemáticas, las funciones derivadas ¿O es que os enseñamos derivadas para machacaros? ¿Es que somos tan tontos que queremos ahí, haceros pasarlo fatal con las matemáticas para nada?” Entonces, cuando ellos ven que tienen que indagar en lo que ya saben porque todo forma parte de un todo, digamos que es como…la frase de los alumnos, que lo explican mucho mejor a veces que nosotros es “por fin me he dado cuenta de para qué llevo estudiando tres años”, porque lo tienen todo como desmenuzado. Son como asignaturas estanco, no les damos esa coherencia que tendrían que tener.
E: Un enfoque integrador.
P8: De conocimiento evolutivo, es decir, tú vas aumentando tus conocimientos, pero porque vas aumentando tu capacidad objetiva de análisis. Tú vas creando conocimiento en ti mismo con todas las aportaciones de otras asignaturas. Si a lo que te dedicas es a estudiar una cosa, a soltarla en el examen y ya me he olvidado porque ya he pasado primero, la hemos liado, porque es que has pasado primero, pero no te has creado el pozo suficiente para que en segundo te aporte mucho más que lo que has hecho en primero, si quieres en un nivel un poco más difícil, pero no, porque parece que hasta bachillerato los cursos van aumentando el análisis. Lo que cambia en la facultad son los contenidos temáticos porque los hemos repartido en tiempo, pero no hay esa progresión de dificultad en conocimientos en muchas asignaturas, ¿no? Lo que tienes que ver es eso, cómo los interrelacionas ¿Por qué estudio sociología? Bueno pues porque en economía los sentimientos son fundamentales y los individuos tenemos actos individualizados, pero generalmente actos conectados colectivamente, entonces hay que entender los procesos sociológicos ¿Y por qué tenemos que estudiar redes? Porque es que las redes son fundamentales en el desarrollo sociológico ¿Y por qué marketing? Porque ahora tú te vas a comprar las cosas al FNAC a través de Internet; tienes que ver toda esa trayectoria, si no ves todo eso, ahora llego yo y ¿qué te cuento? Si tienes que hacer un análisis del comportamiento del consumo de los españoles, me tienes que hacer sí o sí como variable explicativa la generalización del uso de compras por medios informáticos, porque ha cambiado la ruta de comportamiento.


Documento:		4. Ciencias Sociales y Jurídicas\P9 CCSS Creencias
Peso:	0
Posición:	46 - 51
Código:	1. Creencias\Proceso de enseñanza y aprendizaje\1.9. Claves para el aprendizaje
E: Sí, sí. Y desde tu punto de vista, ¿qué es lo más importante para que el alumnado pueda aprender adecuadamente?
P9: ¿El alumnado en general dices?
E: Sí.
P9: Yo creo que primero te tiene que gustar lo que estás contando. Si no te crees lo que les están contando es muy difícil que les transmitas a ellos el interés por tu asignatura, y luego tienes que utilizar el lenguaje adecuado para ellos. Es verdad que a veces nos encantan los tecnicismos porque así parece que sabemos más, y está bien que se los contemos porque luego ellos se los van a encontrar, pero que se los contemos como tecnicismos y no como parte propia de nuestro lenguaje, que al final yo creo que les hacen más complejas asignaturas que deberían ser más sencillas y buscar la forma de que realmente ellos vean lo práctico que tiene lo que les estamos contando.
E: La utilidad.
P9: Sí, el verlo en plan teórico, es muy complejo para todos. Para mí la primera. Yo me pongo en su puesto y me digo “¿cómo me gustaría a mí que me contaran esto?”. Trato de contármelo de una forma que yo creo que para mí me resultara sencillo y sobre todo recordarlo, porque a mí lo que me gustaría es que luego no tuvieran que estudiar mucho. Yo se los digo “si estáis pendientes en clase de las cosas que contamos, luego solo tenéis que leer vuestros apuntes. Luego va a venir a la memoria todo.”, porque trato de dárselo todo muy masticado, ¿no? para que realmente les resulte más cómodo el aprenderlo y no se me desconecten en clase. Porque claro, si te pones muy filosófico o muy complejo, esto les lleva a perder el interés y, aunque estén ahí, no se van a enterar de nada.


Documento:		4. Ciencias Sociales y Jurídicas\P10 CCSS Creencias
Peso:	0
Posición:	55 - 56
Código:	1. Creencias\Proceso de enseñanza y aprendizaje\1.9. Claves para el aprendizaje
E: Bueno, vamos a seguir para que la cosa sea rapidita. Vamos a hablar ahora del proceso de enseñanza aprendizaje. Desde tu punto de vista, qué es lo más importante o lo que no puede faltar para que se dé un aprendizaje adecuado.
P10: Probablemente, ganas de aprender y esfuerzo por aprender. Yo entiendo que una cosa es enseñar y que otra cosa es aprender. Aprende el estudiante, entonces, es decir, por más que tú te empeñes en intentar transmitir las cosas, si luego no hay un esfuerzo por parte del estudiante en el proceso de aprendizaje, pues es como echarle piedras al campo, o algo así, no sé, ¿vale? Y esas cosas, digamos, para mí, ahí hay un problema ahora mismo en general, y un problema social, y es que digamos, ahora mismo, se ha ido deslizando hacia abajo el listón de aprendizaje, para un aprendizaje correcto. Entonces, al final ahí vas viendo de todo, desde gente deslizando hacia abajo también su esfuerzo por enseñar, y entonces hay ahí un círculo que me parece peligroso y no muy bueno para la universidad, al menos, como yo la entiendo, porque, digamos, como si fuera necesario llevarnos a algo parecido a una formación profesional, cosa que hacemos muy mal, muy mal, porque no es lo nuestro…y se va degradando un poquito el nivel y la cualificación de los egresados. Y, ahí, yo creo que hay un problema doble. Y creo que un profesor tiene que esforzarse por enseñar y transmitir adecuadamente, pero eso es como predicar en el desierto si no tienes por la otra parte el esfuerzo por aprender y tal…y hay gente que a lo mejor se sienta mira una cosa y “lo tengo claro”, y hay gente que puede necesitar tres días, no sé.


Documento:		4. Ciencias Sociales y Jurídicas\P11 CCSS Creencias
Peso:	0
Posición:	47 - 48
Código:	1. Creencias\Proceso de enseñanza y aprendizaje\1.9. Claves para el aprendizaje
E: Muy bien. Y en cuanto a los procesos de enseñanza-aprendizaje, qué crees que es lo más necesario para que el alumnado aprenda, en general.
P11: Que el alumno se sienta protagonista de su aprendizaje, que todo lo que aprenda es para él, no porque yo lo digo, sino porque él lo entienda y lo asuma. Y que le vale para algo, que hay mucha paja en la universidad, y nosotros que tenemos una experiencia profesional fuera, pues muchas veces de la paja decimos “pero esto, esto y esto es para toda la vida, que no se os olvide”, y claro, les pincho, les hago reaccionar para que ellos entiendan que el concepto que estamos dando es aplicable hoy ya a su propia familia si quisieran montar un negocio. O sea, quiero que vean las cosas, no me gusta que se las aprendan de memoria, quiero que entiendan todo lo que explicamos.


Documento:		4. Ciencias Sociales y Jurídicas\P11 CCSS Creencias
Peso:	0
Posición:	52 - 52
Código:	1. Creencias\Proceso de enseñanza y aprendizaje\1.9. Claves para el aprendizaje
Digamos, ese componente físico, pero luego hay un componente mental, que es la motivación, y esto es igual, si tú tienes una discapacidad y estás motivado, vas a aprender. Si tú no tienes una discapacidad funcional como esta que estamos tratando y no tienes motivación, no vas a aprender. Entonces, para mí, el elemento motivador es el más importante de todos, no la capacidad o discapacidad que pueda tener.


Documento:		4. Ciencias Sociales y Jurídicas\P12 CCSS Creencias
Peso:	0
Posición:	52 - 53
Código:	1. Creencias\Proceso de enseñanza y aprendizaje\1.9. Claves para el aprendizaje
E: Desde tu punto de vista, ¿qué es lo más importante para que el alumnado pueda aprender adecuadamente?
P12: El interés que le pongan al trabajo, las horas que le dediquen.


Documento:		4. Ciencias Sociales y Jurídicas\P13 CCSS Creencias
Peso:	0
Posición:	39 - 40
Código:	1. Creencias\Proceso de enseñanza y aprendizaje\1.9. Claves para el aprendizaje
E: ¿Y qué crees que es lo más importante para que el alumnado aprenda?
P13: Que ellos se sientan cómodos con todos los recursos que están a su alcance. Porque de lo contrario, si ya de partida tienen las barreras para entrar a la universidad, llegan tarde a las clases, no se pueden sentar, no ven la boca del profesor cuando explica o no ven la pizarra, pues estamos conjurando al fracaso. Es decir, fundamental eliminar las barreras directas, garantizar que el alumno pueda tener acceso a lo que explicas al mismo nivel que el resto. Y luego, en la medida en que el estudiante lo necesite y está involucrado en la asignatura, que el profesor haga accesibles las partes que son más inaccesibles para su propia necesidad de esa asignatura. Que haya cierta colaboración por parte del profesor cuando el estudiante muestra su interés por superar la asignatura.


Documento:		4. Ciencias Sociales y Jurídicas\P14 CCSS Creencias
Peso:	0
Posición:	40 - 41
Código:	1. Creencias\Proceso de enseñanza y aprendizaje\1.9. Claves para el aprendizaje
E: Y, si nos adentramos un poco en el tema de la enseñanza-aprendizaje, lo que ocurre en el aula, ¿vale? Desde tu punto de vista, qué es lo más importante para que el alumnado pueda aprender. Me refiero a qué condiciones deben darse, qué tiene que pasar…
P14: ¿Para que aprendan? Pues, en primer lugar, disposición y cierto aliciente, cierta motivación, ¿no? Entonces, hay estudiantes que tienen una motivación ya de fábrica, por defecto, porque tienen una disposición y les interesa el tema. Entonces, con esas personas, pues el proceso intuyo que será más sencillo, pero hay otros que no tienen motivación hacia un objetivo concreto y pueden ser más reacios. Entonces, esas personas, les es necesario continuamente recordarles para qué sirve la asignatura, qué implicaciones tiene, qué consecuencias tendrá eso para su futuro trabajo en el mercado laboral… Muchas veces preguntan, ¿y esto para qué sirve? Siempre, para esas personas que son reacias por defecto, es recordarles continuamente o darles la analogía de “esto en el mundo real qué implicaciones tiene o qué significa”. 


Documento:		4. Ciencias Sociales y Jurídicas\P15 CCSS Creencias
Peso:	0
Posición:	52 - 53
Código:	1. Creencias\Proceso de enseñanza y aprendizaje\1.9. Claves para el aprendizaje
E: Y, en cuanto a los profesos de enseñanza y aprendizaje, desde tu punto de vista, qué es lo más importante para que el alumnado aprenda. Qué dirías que no puede faltar.
P15: Qué no puede faltar…pues que el alumnado estudie.


Documento:		4. Ciencias Sociales y Jurídicas\P15 CCSS Creencias
Peso:	0
Posición:	110 - 113
Código:	1. Creencias\Proceso de enseñanza y aprendizaje\1.9. Claves para el aprendizaje
E: Vale. Y como docente, qué estrategias de enseñanza conoces que motivan y favorecen más el aprendizaje de los estudiantes en general.
P15: ¿Estrategias?
E: Sí, estrategias que tú utilices en clase o que conozcas que sepas que les motivan más.
P15: Pues sinceramente, no sabría decirte. Lo que motiva más es que ellos deseen estudiar lo que están estudiando. Motivación propia.


Documento:		4. Ciencias Sociales y Jurídicas\P17 CCSS Diseños
Peso:	0
Posición:	2 - 3
Código:	1. Creencias\Proceso de enseñanza y aprendizaje\1.9. Claves para el aprendizaje
E: En el proceso de enseñanza aprendizaje, para ti, ¿qué es lo más importante que el alumno aprenda?
P17: Conceptos. Me niego a que aprendan de memoria definiciones, quiero que las entiendan. Hay una definición que les di la semana pasada que es, por ejemplo, la de la elasticidad, que se aplica en muchísimos campos y yo quiero que ellos entiendan el concepto de elasticidad porque se aplica en muchas ocasiones, es uno de los conceptos que más se aplica en realidad, es decir, cuando mido el efecto de una acción, estoy explicando lo que es el concepto de la elasticidad. Si yo aplico una fuerza sobre un artículo, se mueve hasta que se rompe, pues igual es la oferta y la demanda, entonces quiero que lo entiendan porque en los mercados se utiliza mucho y si ellos…Ellos son estudiantes de empresariales, no son de economía, pero cuando ven marketing y ven mercados lo van a usar mucho, entonces, yo quiero que se queden con el concepto, a mí no me interesa que, como papagayos, me digan la elasticidad significa esto, na, na, na y no sepan aplicarlo.


Documento:		4. Ciencias Sociales y Jurídicas\P18 CCSS Creencias
Peso:	0
Posición:	58 - 59
Código:	1. Creencias\Proceso de enseñanza y aprendizaje\1.9. Claves para el aprendizaje
E: Desde tu punto de vista, qué es lo más importante para que un alumno aprenda.
P18: Hombre, principalmente, veo primero cuál es su disposición, para que un alumno aprenda tiene que estar dispuesto a aprender. Si un alumno viene “vaya rollo, vaya esto y lo otro”, es decir, es su disposición. Segundo, fomentar que, de vez en cuando, den su opinión, no utilizar un lenguaje excesivamente técnico que haga que se pierda, porque como lo pierdas al principio, lo perdiste ya. Entonces, continuamente tienes que estar haciéndole llamadas de atención y, después ya, pues es el hecho de que una asignatura te guste, ¿no? 


Documento:		4. Ciencias Sociales y Jurídicas\P19 CCSS Creencias
Peso:	0
Posición:	38 - 39
Código:	1. Creencias\Proceso de enseñanza y aprendizaje\1.9. Claves para el aprendizaje
E: Desde tu punto de vista, ¿qué es lo más importante para que el alumnado pueda aprender adecuadamente? 
P19: Pues vuelvo a incidir en que lo más importante es saber cómo transmitir los contenidos que se tienen que impartir. Además, otra cosa muy importante es conocer los beneficios que te dan las nuevas tecnologías, porque hay que estar actualizado en ese campo también, creo que proporcionan herramientas muy potentes para aprender.


Documento:		4. Ciencias Sociales y Jurídicas\P20 CCSS Creencias
Peso:	0
Posición:	44 - 45
Código:	1. Creencias\Proceso de enseñanza y aprendizaje\1.9. Claves para el aprendizaje
E: Vale. Y desde tu punto de vista, qué dirías que es lo más importante para que el alumnado pueda aprender, el alumnado en general.
P20: Adaptarse y que vean la importancia de lo que estás explicando, porque algunas veces o te quedas en la parte teórica y no les haces ver la parte práctica. Entonces, claro, cuando llevas un caso práctico dices “a ver, has tenido una multa, has tenido tal…”. Entonces ya ven ese valor añadido de las cosas que tienen que aprender. Entonces, bueno, en ese aspecto yo creo que hay que adaptarse.


Documento:		4. Ciencias Sociales y Jurídicas\P21 CCSS Creencias
Peso:	0
Posición:	82 - 91
Código:	1. Creencias\Proceso de enseñanza y aprendizaje\1.9. Claves para el aprendizaje
E: Creo que la pregunta que le voy a hacer está un poco respondida, pero bueno. Desde su punto de vista, ¿qué es lo más importante para que el alumnado pueda aprender adecuadamente? Creo que ya, un poco, lo hemos ido viendo a lo largo de las preguntas.
P21: Sí, pero vamos, en este caso, ahí si tengo yo una respuesta concreta, y es el interés, por parte del alumnado, porque, insisto, esa es otra de las…a ver, de mis luchas, digamos, yo parto, y creo que no estoy equivocado, de que hay un porcentaje muy alto de alumnos, que están en la universidad como si fuera una continuidad del sistema de enseñanza obligatorio. La vida es estar en el colegio, estar en el instituto, estar en la universidad, casarme y tener hijos, y encontrar un trabajo, ¿vale? En este proceso. He cogido esto, algunos te dicen que era por vocación, o porque tienen una idea equivocada o no, o sea, por las circunstancias que sea, pero yo siempre digo “la universidad no es el sistema de enseñanza obligatoria que tú tienes cuando eres adolescente, rebelde, que, además, ejercitas la rebeldía diciendo pues no estudio”, y ahí está el profesor, que eso sí que tiene también mérito de tú tener que estar convenciéndolo para que estudie y al mismo tiempo incentivándole el estudio y tal…yo siempre digo “señores, ustedes están aquí porque quieren ser periodistas”.
E: Efectivamente.
P21: Yo doy una asignatura que es Fotoperiodismo, que, insisto, yo estoy para orientarlos, y digo “señores, por si alguien todavía tiene la idea del periodismo tradicional donde existía el plumilla y el reportero gráfico, que sepa que eso ha desaparecido, o sea, hoy no dirigen ustedes su especialidad hacia la pluma o hacia la cámara de fotos, hoy ustedes son un multimedia, que tienen que saber manejar, por tanto, estamos ante una asignatura de algo que para vuestro futuro profesional es imprescindible”, aun así, hay alumnos que dicen “es que yo no quiero hacer fotos”, y, aun así, a partir de ahí, lo único que quieren saber es cómo quitarse la asignatura de encima. Con ese alumnado es imposible trabajar, es más, yo les digo, “es vuestro problema”, yo puedo seguir…porque es un alumnado que frena mucho al grupo, que después te pone…yo el curso pasado, llegaron a presentar, que yo de estas cosas, me río por no llorar, una queja en el departamento, a la comisión de docencia, porque el profesor de Fotoperiodismo hablaba en clase de fotógrafos que no vienen en el programa. Yo, desde la actitud infantil que sé que hay detrás de eso, es el alumno que ha cogido el programa, que lo que quiere es estudiarse, pensando en el examen y en aprobar, y diciendo, “está metiendo en el examen contenidos que no vienen en el programa”, y hablar de un fotógrafo es llegar un día a clase diciendo que le acaban de dar unos Per fotos a fulanito de tal y hablando de su obra, al que le acaban de dar el mayor premio de fotoperiodismo que hay…
E: Es actualizar el contenido.
P21: Claro. Y te dicen que como ese no aparecía en el listado, eso no es materia. Por eso te digo, que con ese tipo de alumno que a lo que viene es a querer aprobar, ¿tú que haces? Y yo, ahí sí que tengo muy claro, y lo planteo muy claro desde el primer momento, “yo estoy aquí para ayudar al que quiera aprender, el que no quiera aprender yo no me voy a poner a bailarle jotas para que…”
E: A perder el tiempo.
P21: Claro. Hay muchos alumnos que te hacen perder mucho el tiempo, mucho. Y, desgraciadamente, esto son curvas, y el porcentaje, ya hablamos de porcentajes, y últimamente, los últimos cursos tengo la impresión de que el porcentaje cada vez es mayor, lo que pasa que, afortunadamente te centras en esos alumnos que son los que demandan esa atención, ese papel que tienes. Es como cuando les dices…yo he ido a clase y les digo “señores, ustedes entenderán que la clase no es que yo dicte apuntes, porque si es un capítulo de un tema que es de pura teoría del fotoperiodismo que aquí lo tienen ustedes que está ya en el capítulo de este libro”, que se les da ya el capítulo del libro seleccionado para que se lo lean, “sobre esto haremos referencia, pero léanlo ya ustedes”, “¿pero si no lo dice usted en clase entra en el examen?”. Con ese alumnado, ¿qué haces? Luego, para que aprenda ese alumnado, yo no sé qué hacer, porque hay un límite, “señores, aquí están los argumentos por los que creo que ustedes deben ver el interés que tiene esta asignatura, si aun así ustedes no lo ven, entre otras cosas, porque no tienen el mínimo interés en formarse como periodistas…” A partir de ahí es imposible. Por eso les digo, que para que un alumno aprenda, lo más importante es tener ganas de aprender. Que cuando…siempre te dicen la típica frase “en esta facultad no se aprende nada”, ¿no? O cuando la gente o yo mismo me digo a veces, ¿habré dado lo suficiente para que los alumnos aprendan? Y digo, bueno, vamos a ver, si hay un porcentaje, aunque sea reducido, que han aprendido y que te lo demuestran, porque claro, si fuera solo una cuestión mía, no he motivado, no he sabido transmitir, no lo haría nada bien, no sabría transmitir a nadie. Yo siempre lo hablo, se lo pongo a ellos, sin hablar de mí, de la figura del profesor, sino de ellos como alumnos, digo “señores, quien diga que en esta facultad no se aprende nada, miente, porque os puedo dar el listado de alumnos que han salido de esta facultad tremendamente bien formados y lo están demostrando como profesionales. Si esos profesionales, que ellos mismos reconocen que lo han aprendido en esta facultad, es que en esta facultad se puede. Lo que pasa es que ahora tendréis que plantearos si estáis poniendo el mismo esfuerzo, si estáis poniendo la misma voluntad, si…”, ¿vale? Y lo que yo siempre digo “si alguna vez tienen ustedes una queja, que sea que no les estoy atendiendo en sus necesidades”.
E: Claro.
P21: Pero, si tu necesidad es que necesito saber qué es lo que entra en el examen porque lo que quiero es aprobar, que hasta en eso los atiendo, porque les digo “señores, si lo que quieren es aprobar, yo en el sistema de evaluación tengo una vía, quien quiera cubrir expediente que se prepare esto que yo le hago el examen, allá ustedes, en el mercado se van a estrellar, pero…”, porque te quitas del medio a un alto porcentaje de alumnos que lo único que quieren es aprobar, y, aun así, están después ahí, son unas moscas cojoneras tremendas... Eso…


Documento:		4. Ciencias Sociales y Jurídicas\P22 CCSS Creencias
Peso:	0
Posición:	48 - 49
Código:	1. Creencias\Proceso de enseñanza y aprendizaje\1.9. Claves para el aprendizaje
E: Y, desde su punto de vista, ¿qué es lo más importante para que el alumnado con discapacidad pueda aprender adecuadamente?
P22: Lo más importante, pues no sabría decirte qué es lo más importante. En principio, actitud del propio alumno, ese profesor puede ayudar, pero si la actitud, porque a veces, pues ese alumno es muy tímido, porque se siente inseguro. Eso es natural en su situación, ¿no? Y, a veces, ellos no colaboran, los alumnos, bueno, el que acepten el grupo, unas veces empatizan mucho, otras veces no tanto, y él también tiene que tener una actitud colaborativa, porque no por su situación todo el mundo le tiene que dar todo, ¿no? Entonces, yo creo que esas cosas hay que tenerlas en cuenta. 


Documento:		4. Ciencias Sociales y Jurídicas\P23 CCSS Creencias
Peso:	0
Posición:	54 - 55
Código:	1. Creencias\Proceso de enseñanza y aprendizaje\1.9. Claves para el aprendizaje
E: Qué bien. Y, desde su punto de vista, ¿qué es lo más importante para que el alumnado pueda aprender adecuadamente?
P23: En mi caso, como imparto asignaturas prácticas, pues es el ejercicio o las prácticas en clase y en casa, motivarlos. Como tengo la suerte de que les doy clases de periodismo y ellos entraron aquí a estudiar periodismo, pues lo más importante es vincularlos con la carrera. Hacerles ver lo prácticos que les va a resultar eso cuando lo estén ejerciendo, enseñarles periódicos del día a día y hacerles entender que ellos lo van a necesitar eso, que es una necesidad urgente para ellos. Entonces, tengo suerte también por las asignaturas que imparto.


Documento:		4. Ciencias Sociales y Jurídicas\P24 CCSS Creencias
Peso:	0
Posición:	25 - 25
Código:	1. Creencias\Proceso de enseñanza y aprendizaje\1.9. Claves para el aprendizaje
Mi gran objetivo es tocar emociones, cuando lo haces, se produce un cambio magnífico porque deja entrar la información en la persona, se transforma, hablan del tema con normalidad con la familia, los amigos… Es muy gratificante esa parte e intento ser cercana. 


Documento:		4. Ciencias Sociales y Jurídicas\P25 CCSS Creencias
Peso:	0
Posición:	58 - 61
Código:	1. Creencias\Proceso de enseñanza y aprendizaje\1.9. Claves para el aprendizaje
E: Hablando ahora de los procesos de enseñanza-aprendizaje, ¿qué es lo más importante, desde tu punto de vista, para que el alumnado pueda aprender adecuadamente? ¿Qué variables crees que influyen en el aprendizaje del alumnado?
P25: Es muy importante la motivación, es importante saber con qué motivación vienen ellos al aula, y el interés por la profesión, por la asignatura, el interés que se despierta. Esto es para mí clave.  Si tú no consigues despertar el interés por lo que estás explicando en clase, o por las propuestas que le estás haciendo, entonces, será una memorización y punto. Esto es una cosa, otra cosa es ser capaz de discriminar y seleccionar qué contenidos tienen ellos que trabajar, ya que todo no vale, hay muchos contenidos que no sirven para nada; repetir mucho, repetir las mismas ideas constantemente, ¿eh? es otra tarea muy importante para el alumnado. Y a mí, me parece que es muy importante estar cerca de ellos, estar cerca y disponible para que ellos tengan la confianza de venir a tutorías y de plantearte dudas y qué tú puedas entrar en mayor profundidad con ellos.
E: Mostrarte accesible a ellos, ¿no?
P25: Sí, accesible.
