# Supplementary material for: Is pedagogical training an essential requirement for inclusive education? The case of faculty members in the area of Social and Legal Sciences in Spain
Source: PLoS One. 2021 Jul 2;16(7):e0254250. doi: 10.1371/journal.pone.0254250 (PMC8253417; doi:10.1371/journal.pone.0254250)
Supplement: S1 File — (ZIP) [file pone.0254250.s001.zip › 1.11. ADAPTACIONES (1).rtf]

Documento:		4. Ciencias Sociales y Jurídicas\P1 CCSS Creencias
Peso:	0
Posición:	46 - 47
Código:	1. Creencias\Adaptaciones curriculares\1.11. Adaptaciones
E: En relación con las adaptaciones curriculares, qué opinas sobre la necesidad de realizar adaptaciones en tu asignatura para que los estudiantes con discapacidad puedan aprender.
P1: Pues mira, yo las adaptaciones que he hecho no han sido de contenidos, lo que sí he adaptado son las PPT, o he adaptado la forma en que, si yo pongo una presentación, a un alumno se la doy también en papel en grande para que la pueda seguir desde el papel mejor en su asiento… Ese tipo de adaptaciones. Son adaptaciones de formato, podríamos decir, de forma, pero de contenido no me he enfrentado a ninguna, solamente de forma.


Documento:		4. Ciencias Sociales y Jurídicas\P2 CCSS Creencias
Peso:	0
Posición:	52 - 57
Código:	1. Creencias\Adaptaciones curriculares\1.11. Adaptaciones
E: Con respecto a las adaptaciones curriculares, ¿tú qué opinas sobre la necesidad de hacer modificaciones o adaptaciones en la asignatura para que determinados estudiantes no estén, digamos en desventaja?
P2: Bueno, sí, claro, pero eso lo que no puedes hacer es decir “venga, te voy a quitar la mitad del temario”. No, pero claro, sí se puede hacer, pero es que ahí tampoco podemos matar con tirachinas, a ver a lo que me vengo a referir, que tú tienes que tener en cuenta que tu proyecto docente y todo tiene que tener suficiente margen de actuación para estas personas, porque tú no sabes las necesidades específicas que puede tener cada uno.
E: Y qué tipo de adaptaciones se podrían hacer en tu asignatura.
P2: Pues afortunadamente la tengo planificada de una manera que sí me permitiría tener margen para hacer ciertos tipos de exámenes diferentes: escritos, orales, tipo test, distintos modelos de… Y luego los recursos que tengo son lo suficientemente accesibles como que los recursos, fuentes bibliográficas, páginas oficiales del ministerio o la unión europea son lo suficientemente accesibles como para que cualquier persona pudiera acceder.
E: Todos los materiales son accesibles, ¿no? También para personas con discapacidad.
P2: Perfectamente vamos, porque yo sé que incluso las páginas oficiales del ministerio, tienen acceso al ciudadano con necesidades específicas, ¿no?


Documento:		4. Ciencias Sociales y Jurídicas\P3 CSS Creencias
Peso:	0
Posición:	46 - 49
Código:	1. Creencias\Adaptaciones curriculares\1.11. Adaptaciones
E: Vale. En relación con las adaptaciones curriculares, ¿qué opinas sobre la necesidad de realizar adaptaciones en las asignaturas dirigidas a los estudiantes con discapacidad? 
P3: Esto creo que habría que articular algún tipo de mecanismo desde la propia universidad, no dejarlo todo en manos de los profesores. Sino, que hubiera un mecanismo articulado desde la propia universidad, donde se pudiera hacer esas adaptaciones, pero no dejarlo en manos del profesor porque si no cada profesor va a hacer lo que le parezca bien.
E: En tu asignatura, ¿se puede realizar adaptaciones? ¿qué tipos de adaptaciones se pueden realizar? 
P3: No es que se haya hecho, pero, por ejemplo, la persona con discapacidad, pues tenía un lector de transparencias. Entonces, tenía un programa e iba leyendo las transparencias. Los exámenes, cuando lo hemos hecho, pues lo ha hecho en un ordenador con más tiempo del necesario, hasta un 30 o 40% del tiempo. Siempre, hemos hablado con la persona para ver qué le interesa más, si examen oral o escrito, tipo test. Entonces, siempre hemos hecho adaptaciones, pero hasta donde hemos podido.


Documento:		4. Ciencias Sociales y Jurídicas\P4 CCSS Creencias
Peso:	0
Posición:	47 - 47
Código:	1. Creencias\Adaptaciones curriculares\1.11. Adaptaciones
Los estudiantes con discapacidad, pues puede ocurrir de todo, puede ocurrir que tengan mucho apoyo en las clases, que necesiten que se les refuerce… Puede ocurrir lo contrario, que no puedan venir a clase por el tema de su discapacidad y entonces hay que de alguna forma compensar el hecho de que no venga a clase ayudando entre su esfuerzo y lo que el profesor pueda hacer para que se ponga al día con la asignatura…


Documento:		4. Ciencias Sociales y Jurídicas\P4 CCSS Creencias
Peso:	0
Posición:	50 - 51
Código:	1. Creencias\Adaptaciones curriculares\1.11. Adaptaciones
E: Y ahora, hablando sobre adaptaciones curriculares, qué piensas sobre la necesidad de realizar adaptaciones en tu asignatura.,
P4: Pues opino que hay que afrontarlas si es necesario porque haya personas que lo necesiten.


Documento:		4. Ciencias Sociales y Jurídicas\P4 CCSS Creencias
Peso:	0
Posición:	71 - 71
Código:	1. Creencias\Adaptaciones curriculares\1.11. Adaptaciones
Entonces, ya no…con los estudiantes con discapacidad intelectual, no es el caso que yo he tenido, pero por reflexionar un poco sobre otras discapacidades. Discapacidad de tipo mental, pues que, además, discapacidad física, visual, auditiva… Quizá sean más manejables, pero discapacidad mental…ahí se necesita mucho apoyo, prestar mucha ayuda, darle opciones, trabajar mucho la motivación, sentirse que está apoyado, facilitar la evaluación y no “venga un examen de tres horas”, pues a lo mejor eso no puede ser. En cualquier caso, nosotros como profesores, en la evaluación, a la hora de determinar si el estudiante ha adquirido los conocimientos suficientes para superar y pasar la asignatura, pues plantearse de que otra forma se puede plantear en ese caso… Es que son muchas cosas las que se pueden plantear.


Documento:		4. Ciencias Sociales y Jurídicas\P5 CSS Creencias
Peso:	0
Posición:	60 - 63
Código:	1. Creencias\Adaptaciones curriculares\1.11. Adaptaciones
E: Y, vamos a pasar ahora a hablar sobre adaptaciones curriculares, que es cualquier cambio que tú hagas en la asignatura para favorecer que el alumno aprenda. Entonces, qué opinas sobre la necesidad de realizar adaptaciones en tu asignatura para que el alumnado con discapacidad pueda aprender.
P5: La que puedan demandar, pero no se me ocurre…no sé.
E: Pero qué opinión tendrías al respecto en caso de que tuvieras que hacer alguna.
P5: Claro, sin problema.


Documento:		4. Ciencias Sociales y Jurídicas\P6 CCSS Creencias
Peso:	0
Posición:	40 - 43
Código:	1. Creencias\Adaptaciones curriculares\1.11. Adaptaciones
E: Estupendo. Te voy a hacer algunas preguntas sobre adaptaciones curriculares; nosotros entendemos adaptación curricular, no adaptar un temario, no es eso, adaptación curricular es una adaptación de cualquier cosa, cualquier tipo de adaptación que se haga en una asignatura para facilitar el proceso de enseñanza y aprendizaje a uno o varios estudiantes y, una adaptación curricular puede ser, simplemente, dar más tiempo en un examen a un estudiante que lo necesita o cambiar tus diapositivas y poner el tamaño de letra más grande o cualquier cosa, es decir, la adaptación es un término muy general. Te lo dejo claro porque muchas veces la gente que no somos de pedagogía, cuando nos dicen el término, pensamos que es cambiar el temario, ¿no? 
P6: Sí, sí.
E: Y no es eso en absoluto. Es realizar cualquier adaptación. Entonces, ¿qué opinas sobre la necesidad de realizar adaptaciones en tu asignatura para que los estudiantes puedan aprender?
P6: Creo que, si son necesarias, deberían ser obligatorias, esa es mi opinión, que, si con ello facilitas el aprendizaje de cualquier alumno, deberían incluso obligarte a tenerlas que realizar. Yo estaría encantada, pero como habrá quién no lo esté, creo que se debería de obligar. 


Documento:		4. Ciencias Sociales y Jurídicas\P7 CCSS Creencias
Peso:	0
Posición:	54 - 65
Código:	1. Creencias\Adaptaciones curriculares\1.11. Adaptaciones
E: No sé si alguna vez ha hecho o le han propuesto hacer adaptaciones curriculares. Una adaptación curricular es una medida que se hace al currículum, por ejemplo, o a la metodología, para apoyar el aprendizaje, por ejemplo, una adaptación muy sencilla es lo que usted comentaba antes: el tiempo de examen se amplía para ese alumno porque se entiende que necesita más tiempo para poder desarrollar el examen, o la forma de evaluación se cambia y se hace un examen oral y no un examen escrito, o viceversa, es decir, pequeños ajustes que se hacen para intentar que alumnado tenga igualdad de oportunidades a la hora de aprender o de evaluarse.
P7: De manera informal sí que lo hago, pero no lo tengo normalizado. Es verdad que cuando a veces tengo Erasmus, pues si he puesto un texto muy largo que había que leer pues digo “como hay Erasmus, normalmente, son para estas preguntas son tres cuartos de hora, pues amplio algo más” o a lo mejor el examen de los Erasmus no les pongo el texto y les hago un resumen para que no les cueste tanto.
E: ¿Y solo le da el resumen a los Erasmus? Ese tipo de adaptaciones.
P7: Sí, eso es, sí. Y los alumnos con dificultad, sí que es verdad lo que le comentaba, pues a lo mejor le he dicho “pues si quieres ven a tutoría” y le he hecho el examen en tutorías porque a lo mejor se ponía más nervioso o le costaba más hacerlo o, en algún caso, se lo he hecho oralmente porque tenía dificultades para escribir bien.
E: Y las posibilidades…porque muchas veces los profesores hemos atendido de manera particular, como tú dices, a los alumnos con determinadas características, Erasmus, mayores, con cargas familiares, discapacidad… Esas posibilidades de incorporar esas prácticas a toda la clase; por ejemplo, que cada uno pudiera evaluarse según quisiera, porque a veces, a mí me pasa, por ejemplo, que yo noto que algunos alumnos míos podrían tener mejor evaluación si lo hicieran de manera oral, porque tienen una mejor habilidad para comunicarse. Y no les pasa nada ni tienen discapacidad ni nada, simplemente que les da bien comunicarse.
P7: Yo en eso, como creo que luego en la empresa tienen que afrontar las diferentes modalidades, no hago diferencias. Yo sí es verdad que en el examen les someto a mucha tensión porque sé que luego eso, esa capacidad, es decir, el examen ya no lo veo como una evaluación de los conocimientos, sino que el examen lo veo como un ejercicio de desarrollo y una capacidad, que es la tensión que tienes que tener. No lo veo como algo negativo, esa tensión, al contrario, para el alumno puede ser una experiencia, en ese momento…
E: Traumática.
P7: Sí, pero, sin embargo, es un aprendizaje. Yo siempre digo que se presenten aun pensando que van a suspender porque en sí el propio examen es un proceso de aprendizaje, el someterse a la tensión esa, a la tensión de que lo tienes que cumplir en un tiempo determinado, cómo expresarte… Yo creo que eso no es malo, sí que es verdad que yo combino, por eso hago evaluación continua, combino mucho los métodos de evaluación, en algunos están más fuerte unos, en escritos, hago exposiciones donde todo el mundo tiene que exponer oralmente durante todo el curso, y luego exámenes un poco…siempre son muy prácticos, pero no. No hago esa diferencia de que, si a uno le va mejor lo oral, le hago esa diferencia. Sí en cuanto a esas problemáticas especiales, pues si hay que abordarlo en tutorías, o personas que por cualquier circunstancia no pueden venir al examen, ofrezco alguna flexibilidad, pero sin ser injusto. Que todos puedan estar…
E: ¿Crees que la universidad favorece la educación inclusiva? Favorece la igualdad de trato, la igualdad de aprendizaje…
P7: No, no. Yo sí que lo veo continuamente porque, pues como tengo un seguimiento con antiguos alumnos y…creo que no que, al contrario, mucha rigidez, incluso no solo con personas con discapacidad, sino con personas con problemáticas especiales, familiares, de cualquier circunstancia, y creo que no. Se encuentran con muchas rigideces, incluso con personas, con profesores que son muy rígidos, que no muestran aspecto humano y que, al final, le dejas a ese alumno, le estás formando en una serie de capacidades y valores muy negativos en la sociedad. Yo creo que el profesor debe mostrar y formar en valores y muchas veces yo creo que en eso fallamos en la universidad española. Yo creo que tenemos que hacer mucho esfuerzo y los profesores no establecer tanta distancia con el alumno. Sí que es diferente el profesor…lo que no se puede es endiosar y poner dificultades para que el alumno acuda. Si todos recordamos, yo estudié ingeniería y no recuerdo nunca haber ido a tutorías porque teníamos miedo al profesor. Eso no puede ser. Impide el aprendizaje. Al hacerlo, al final sales de la universidad con un rechazo. Yo de hecho, un rechazo me dirigió completamente a otra…al margen de que, por temas de empleo, dejó de gustarme mi carrera. Y yo creo que eso ocurre mucho en España, que desgraciadamente la enseñanza es una enseñanza con profesores que no están por vocación ahí, que no tienen una formación especial o un poco de empatía hacia el alumno…
E: U otro interés.
P7: Sí, eso. Y los intereses de que hoy se está dando más interés hacia la investigación y a la docencia se la está poniendo…yo estoy totalmente en contra, yo provengo del sector privado y creo que cuando entré aquí, para mí fue un mazazo. No me esperaba yo ver…parece que son todo guetos, cada departamento. Dentro de los departamentos, incluso los profesores, parece que estamos en un monasterio, cada uno en su celda. No hay un compartir los conocimientos. Sin embargo, en el sector privado no ocurría, sino que continuamente estábamos…yo creo que nos queda mucho por hacer, sobre todo en formación humana, yo creo que por experiencia y sin criticar, porque lo mismo me puede ocurrir a mí, pero por lo que oigo de estudiantes, de encontrarse con personas muy duras, que les han hecho mucho daño por lo que decíamos, que es una edad muy conflictiva en una persona y lo que necesitan es una persona que sea un referente y valores, y no encontrarse con personas que les haga difícil su vida porque ya la adolescencia ya tiene su dificultad, que encima ya tenga esa dificultad añadida.


Documento:		4. Ciencias Sociales y Jurídicas\P8 CSS Diseños
Peso:	0
Posición:	20 - 21
Código:	1. Creencias\Adaptaciones curriculares\1.11. Adaptaciones
E: Y, pasando a hablar de las adaptaciones curriculares, qué opinas sobre la necesidad de realizar adaptaciones…
P8: Yo te diría que adaptarlas más en el proceso de aprendizaje que en contenidos o resultados. Me explico, yo creo que es necesario un acercamiento muy, muy directo y mucho más personalizado con ese tipo de personas, con personas que tienen discapacidad, o sea, necesitan una atención especial, y eso lo puedes desarrollar haciendo distintas actividades y también viendo la respuesta del estudiante a esas propuestas que tú le haces. Yo te pongo ejemplos porque no te puedo hablar de términos genéricos, sino de mi experiencia, ¿vale? Entonces, por ejemplo, esta persona tenía, a ver, no dificultades, pero sí temor, por ejemplo, a intervenir en un grupo grande en el aula, entonces, yo en las clases, sobre todo al final, en las preguntas de las minis, les hago una pregunta en clase y es una manera de controlar la asistencia, y después de que me entregan los resultados, porque estoy convencida de que sabe perfectamente lo que tienen que contestar. Entonces, esta persona nunca participaba, pues nunca se me ocurrió insistir porque sabía que le podía poner en una situación incómoda. A continuación, si algún día tenía que salir a la pizarra, yo tampoco le insistía, pero hablando con él en las tutorías que hacía individualizadas, le dije que si le daba terror o pánico, o simplemente quería darle la oportunidad, entonces, al final del curso el que levantaba la mano para salir a la pizarra era él porque se había sentido integrado y, digamos, que no el punto de mira en valores negativos, sino como uno más. Pero eso nos llevó mucho tiempo, en cómo te sientes, si la próxima sesión, ¿prefieres que te pregunte o que no te ponga en la tesitura?... Quiero decir, como un acercamiento muy personal. A la hora de hacer los trabajos, por ejemplo, yo pensaba que podía resultar muy complejo incorporarle en un grupo de trabajo porque les hago trabajos individuales y en grupo, ¿no? Entonces, al principio le propuse que si quería hacer los trabajos individuales. Cuando veía cómo se desarrollaba y cómo hacía los trabajos en grupo, lo que hice fue, lanzar a través de Moodle una pregunta de cuántos grupos había solo integrados por tres personas donde hubiese cabida para otra persona que se incorporara y daba el nombre. Entonces, sí había tres o cuatro grupos que me dijeran “nosotros”, pues al final se incorporó en un grupo y estuvo trabajando. Es decir, pues eso, facilitándole su condición social.


Documento:		4. Ciencias Sociales y Jurídicas\P9 CCSS Creencias
Peso:	0
Posición:	62 - 65
Código:	1. Creencias\Adaptaciones curriculares\1.11. Adaptaciones
E: Sí, sí. ¿Y qué opinas sobre la necesidad de realizar adaptaciones en tu asignatura para que los estudiantes con discapacidad puedan aprender?
P9: ¿Adaptaciones del tema del material, por ejemplo? Me parecería genial y fantástico. Sobre todo, cuando tienes alumnos con alguna discapacidad física y tal, pues si pudieran tenerlo en Braille o con anterioridad pues me parecería perfecto. Eso sería genial.
E: Tú claro, en este caso, no tienes esa experiencia por lo que me has contado antes porque la pregunta un poco iba dirigida a qué te supone como docente adaptar una asignatura para hacerla más inclusiva. Pero, aunque no sea una adaptación de materiales, porque no has tenido ningún estudiante con discapacidad visual o auditiva, yo creo que tal vez has tenido que adaptar en algún momento de tu carrera profesional algún contenido, algún ejercicio para…por dificultad, por lo que decías del idioma…
P9: A los Erasmus. Claro. Realmente es un tema de tiempo, o sea, es un poco dedicarle, pero que te vuelvo a decir que al final tú estás aprendiendo. Si yo tengo que buscar marcas similares en Italia, en Francia o en Alemania, pues en ese momento puede parecer que estoy perdiendo el tiempo para esto, pero yo estoy aprendiendo, o sea, eso me sirve porque a los alumnos también les gusta ver ejemplos de otros países, que no solo te centres en España. Con lo cual, si ya lo has aprendido pues lo has tenido que buscar para esos alumnos que son de fuera, pues mira, para lo próxima ya tengo ejemplos para que ellos puedan ver cómo se implementan determinadas cosas en España, en Italia, en Francia o en China, que también tengo muchos alumnos chinos. Además, les pregunto “¿y eso cómo se hace en China?” porque claro, yo no tengo ni idea, ¿no? Pues como cuando hablas de las costumbres “¿y qué desayunáis en China?”, pues te dicen “pues sopa de…” uno me dijo que depende de la zona de China que seas. Uno me dijo “sopa de nido de golondrina” que dije “madre mía, si me tengo que tomar una sopa de nido de golondrina…”. Pues lo aprendes, luego cuando les pones los ejemplos igual es de eso de lo que se acuerdan, del ejemplo que les has puesto sobre cualquier cosa que era diferente a lo que ellos están acostumbrados. Entonces, bueno, pues sí, les tienes que dedicar más tiempo, pero al final también te enriqueces. Yo creo que todo tiene una vuelta.


Documento:		4. Ciencias Sociales y Jurídicas\P10 CCSS Creencias
Peso:	0
Posición:	61 - 62
Código:	1. Creencias\Adaptaciones curriculares\1.11. Adaptaciones
E: Entonces, ¿has realizado alguna adaptación curricular, algún ajuste…?
P10: Vamos a ver, por eso te digo, que yo recuerde, en un caso, el del chico este, donde al final lo que hicimos fue casi examinarlo tema por tema porque no había forma de que aprobara, y, aun así, por los pelos. Y, no me siento especialmente ni contento ni orgulloso ni nada de ese caso, porque no entiendo que…no sé, es como alguien que está cojo, pues no puede ir a una carrera, no sé, no pasa nada, pero no…hay cosas que a mí no me cuadran porque yo veía que este chico tenía muchas dificultades de…claro, dices, cuando acabe es que va a mezclar las cosas, va a tener un título, pero…entonces, yo no lo veía claro, no lo veía claro. Y tuvo, pues años, es decir, facilidades de “mira este tema y la semana te quiero ver aquí y con todas las dudas que tengas”, y el chico lo hacía, muy cumplidor y tal. Pero luego, llegaba el momento y…yo con lo que tengo, con ayudar a la gente, no tengo mayor problema, ahora con que se le cambie el listón, no me siento nada cómodo, porque pienso que es un fraude de alguna manera.


Documento:		4. Ciencias Sociales y Jurídicas\P11 CCSS Creencias
Peso:	0
Posición:	55 - 56
Código:	1. Creencias\Adaptaciones curriculares\1.11. Adaptaciones
E: Y en cuanto a las adaptaciones curriculares, qué opinas sobre la necesidad de realizarlas para los estudiantes con discapacidad.
P11: Me parece bien, pero no quiero ser políticamente correcto en eso de la discriminación positiva. Como todo, es matizable. No más privilegios, sí más adaptabilidad, es decir, ser flexible para que puedan llegar al objetivo final, pero es el mismo para todos.


Documento:		4. Ciencias Sociales y Jurídicas\P12 CCSS Creencias
Peso:	0
Posición:	64 - 65
Código:	1. Creencias\Adaptaciones curriculares\1.11. Adaptaciones
E: Estupendo. ¿Qué opinas sobre la necesidad de realizar adaptaciones en una asignatura para que el estudiantado con discapacidad pueda aprender? 
P12: No lo sé, depende de qué tipos de adaptaciones. Digamos que si la persona puede conseguir lo mismo que puede conseguir un estudiante sin discapacidad, pues hay que dar la máxima facilidad, pero no lo sé, para mí esto es demasiado complicado en mi especialidad. Yo creo que hay que darles facilidades al alumnado con discapacidad, pero creo que hay que exigirles exactamente lo mismo que se le exige al alumnado sin discapacidad. Es decir, si el estudiante sin discapacidad tiene que tener una serie de contenidos mínimos, pues los estudiantes con discapacidades también los tienen que tener. Lo que pasa que si estos necesitan más tiempo de estudio, más flexibilidad, pues por supuesto se le ofrece, pero al final tiene que terminar el curso en unas condiciones similares como lo termina el resto de sus compañeros. 


Documento:		4. Ciencias Sociales y Jurídicas\P13 CCSS Creencias
Peso:	0
Posición:	45 - 48
Código:	1. Creencias\Adaptaciones curriculares\1.11. Adaptaciones
E: Con respecto a las adaptaciones curriculares, qué opinas sobre realizarlas.
P13: Yo creo que gracias al desarrollo de las nuevas tecnologías, ahora las herramientas son globales y más inclusivas, lo cual facilita ya la inclusión en la educación. Estas herramientas favorecen a todos y creo reducen la necesidad de hacer grandes cambios. Considero que si son necesarias pequeñas modificaciones dentro de lo razonable que permitan a cada uno desarrollar sus capacidades, es bueno realizarlas. Es la base del derecho a la educación, del desarrollo del ser humano, y del desarrollo social inclusivo. 
E: ¿Y en tu asignatura es posible realizar estas adaptaciones?
P13: Estamos sujetos a unas guía docente, y a unos planes de estudio públicos cuyos contenidos se someten a un proceso de verificación muy detallado. Hay muy poca flexibilidad en ese sentido.  Mi asignatura es contabilidad. Sí que favorece el que la asignatura, a pesar de que es presencial, tiene todos los recursos docentes y actividades de evaluación propios y típicos de un aprendizaje virtual, salvo el examen final. No tengo mucho conocimiento del tema de las adaptaciones curriculares, por lo que no sé de qué alcance estamos hablando. Particularmente no soy partidaria de reducir contenidos de aprendizaje, si es eso de lo que estamos hablando, porque se verían mermadas las competencias propias de los profesionales que estamos formando. Sí que soy partidaria de afrontar el tema con recursos específicos como los que hemos hablado antes (colgar materiales en el AV, hacer las pruebas escritas con apoyo, con mayor tiempo…) No sé si esto último es adaptación curricular…


Documento:		4. Ciencias Sociales y Jurídicas\P14 CCSS Creencias
Peso:	0
Posición:	50 - 51
Código:	1. Creencias\Adaptaciones curriculares\1.11. Adaptaciones
E: Vale. Y hablamos ahora de las adaptaciones curriculares que llamamos, de modificar cualquier elemento del currículum, sean materiales, objetivos, forma de acceder… Lo que sea, ¿vale? Modificarlo para que todos los alumnos puedan conseguirlo, ¿no? Entonces, qué opinas sobre la necesidad de adaptar asignaturas en la universidad para que todos los estudiantes puedan aprender.
P14: Pues, es realmente difícil porque, bueno, claro, quién determina y cómo determina cómo hacer esa adaptación para que tengan todos las mismas oportunidades. Pues soy consciente de que eso no debe ser muy fácil, ¿no? Porque, al final tienes que intentar eso, que las oportunidades sean simétricas, y no dar ventajas extra a nadie, más de lo que merece, ¿no? Entonces, bueno, yo en ese sentido, en la USE, cuando dan esas propuestas, yo desde luego, no sería capaz de decidir “pues esta persona necesita más tiempo, o un examen distinto…”, puede ser peligroso porque tú velas porque todos se enfrenten y demuestren el mismo nivel de adquisición de conocimientos. Entonces, lo que desvirtúe eso, ¿no? Para hacer una discriminación positiva, aunque sea, pues hay que hacerlo con mucho cuidado, ¿no? Pues, bien, es necesario, lo entiendo, pero también con mucho cuidado, ¿no? 


Documento:		4. Ciencias Sociales y Jurídicas\P15 CCSS Creencias
Peso:	0
Posición:	62 - 67
Código:	1. Creencias\Adaptaciones curriculares\1.11. Adaptaciones
E: Claro. Y, ahora vamos a hablar sobre las adaptaciones curriculares, que nosotros entendemos adaptación como cualquier tipo de modificación que se haga, por pequeña que sea, para que los estudiantes con discapacidad puedan aprender, no tiene por qué suponer una modificación de los objetivos o de los contenidos, puede ser de cualquier cosa. ¿Qué opina sobre la necesidad de realizar adaptaciones en su asignatura?
P15: ¿Del currículum?
E: En general. Me has comentado al principio que sí que hacíais, por ejemplo, dar más tiempo en los exámenes, pues cosas de ese tipo.
P15: Sí, sí, eso sí. Si necesitan más tiempo para hacer un examen de dos horas, pues se les da dos horas y media o el tiempo que necesiten, eso sí.
E: Porque, ¿has realizado algún tipo de modificación más aparte de esa del examen?
P15: A ver…no. Dar más tiempo en el examen o en algún caso de algún alumno con deficiencia visual, el examen se le proporciona en un tamaño de letra mayor o se le permite, por ejemplo, salir al cuarto de baño con más frecuencia o… Eso, sí.


Documento:		4. Ciencias Sociales y Jurídicas\P16 CCSS Creencias
Peso:	0
Posición:	40 - 41
Código:	1. Creencias\Adaptaciones curriculares\1.11. Adaptaciones
E: Con respecto a las adaptaciones curriculares, ¿tú qué opinas sobre la necesidad de realizar ajuste en las diferentes asignaturas para favorecer o para que los estudiantes con discapacidad estén en igualdad?
P16: Pues hay que hacer la adaptación a las discapacidades que existen en cada curso. 


Documento:		4. Ciencias Sociales y Jurídicas\P18 CCSS Creencias
Peso:	0
Posición:	64 - 65
Código:	1. Creencias\Adaptaciones curriculares\1.11. Adaptaciones
E: Tengo aquí una serie de preguntas, pero creo que ya me las has respondido, qué opinas sobre la necesidad de realizar adaptaciones para que los alumnos puedan aprender, de hecho, cuando ha sido necesario lo has hecho, es que las tienes previstas por adelantado.
P18: Sí, claro, es que, realmente, yo si es con este alumno, yo no le pregunto a él, yo le digo “¿tienes esto claro?”, “si tenemos aula de informática coges el ordenador, si no, pues puedes traer el ordenador”, pero él y cualquiera. No es adaptar solo a él, sino en general, bueno, a cualquier tipo de circunstancias que existan.


Documento:		4. Ciencias Sociales y Jurídicas\P18 CCSS Creencias
Peso:	0
Posición:	66 - 67
Código:	1. Creencias\Adaptaciones curriculares\1.11. Adaptaciones
E: Si hacen un examen necesitan más tiempo, se le haría esa excepción, pero también a otra persona, ¿no?
P18: Claro. Se le haría. Lo único es que hay también se tiene que saber y solicitar, entonces, yo, hombre, sobre todo, porque no podemos ser arbitrarios, yo…a mí no me puede decir una persona “es que a él le has dejado y a mí no”, yo se lo dejo por este motivo, entonces, lo tendrá que solicitar, pero en un examen oral como él hizo, en este caso, este alumno hizo un examen escrito, que yo estuve presente, y esta persona tardó más, lógicamente. Él era discapacitado físico y psíquico, entonces, era…se le dio un poco más de tiempo, entonces, sí es adaptarlo, pero que, con esta persona no he adaptado, sino que esa posibilidad se la permitía también a otros que quisieran.


Documento:		4. Ciencias Sociales y Jurídicas\P19 CCSS Creencias
Peso:	0
Posición:	46 - 47
Código:	1. Creencias\Adaptaciones curriculares\1.11. Adaptaciones
E: ¿Qué opinas sobre la necesidad de realizar adaptaciones en su asignatura para que los estudiantes con discapacidad puedan aprender?
P19: Pues que son necesarias, porque si no llegas a ese alumno…creo que son necesarias.


Documento:		4. Ciencias Sociales y Jurídicas\P20 CCSS Creencias
Peso:	0
Posición:	52 - 53
Código:	1. Creencias\Adaptaciones curriculares\1.11. Adaptaciones
E: Claro. Y con respecto a las adaptaciones curriculares, ya me has comentado que consideras fundamental ir adaptándote, ¿no?
P20: Sí, sí.


Documento:		4. Ciencias Sociales y Jurídicas\P21 CCSS Creencias
Peso:	0
Posición:	104 - 107
Código:	1. Creencias\Adaptaciones curriculares\1.11. Adaptaciones
E: Y, ¿qué opina de la necesidad de realizar adaptaciones en su asignatura para que los estudiantes con discapacidad puedan aprender?
P21: ¿Qué opino de si es bueno o es malo? Es que...
E: Sí, la necesidad que hay para realizar ese tipo de adaptaciones. ¿Considera que son trato de favor, que aprenden menos, que no aprenden menos, que es positivo, que no es positivo...?
P21: No, positivo es porque es lo que hablamos. O sea, si tu problema está en una... en algo tan simple como que para entrar en un aula está pensado para un perfil de persona que ocupa un asiento, si tú vienes con una silla de ruedas, tienes que tener desde la infraestructura física...yo leí hace poco que una alumna matriculada en una universidad, donde para acceder a su aula la tienen que subir los compañeros en la silla de ruedas por las escaleras porque está todavía en un edificio antiguo. Entonces, claro que tú tienes que facilitar. Tú tienes que facilitar eso o no tienes que facilitar el que, si yo estoy entregando una documentación y tú eres invidente, pues ojalá, lo facilite la ONCE o ellos mismos a través de la ONCE. Pero, claro que sí tendría que tener la universidad algo que...fuera la universidad la que a mí me dice desde secretaría... Pero a mí no me llega desde secretaría ningún tipo de información en ese sentido. Ojalá funcionáramos de manera en la que a mí me dice la universidad “en la lista de clase de alumnos matriculados para el curso que viene tiene usted un alumno invidente”. Y que yo tuviera en mi propia facultad o en el seno de mi departamento la maquinita que...el material que cuando yo le digo “lean ustedes esto” se lo pueda ya directamente adaptado en braille o en el método que ellos necesiten. Por eso, aquí eso lo paliamos con lo que te comentaba, que yo le digo “oye, lo que necesites”. Y, entonces, normalmente, te dicen “bueno, pues ese material, ¿le importa dármelo en un pendrive o mandármelo?”. Que yo lo mande a la ONCE y ellos me... Y entonces, vamos solucionando los problemas sobre la marcha.


Documento:		4. Ciencias Sociales y Jurídicas\P22 CCSS Creencias
Peso:	0
Posición:	56 - 59
Código:	1. Creencias\Adaptaciones curriculares\1.11. Adaptaciones
E: Y, ¿qué opina sobre la necesidad de realizar adaptaciones en su asignatura para los estudiantes con discapacidad?
P22: Bien, necesarias. Se hacen adaptaciones ¿Te refieres físicas o…?
E: Sí, las que sean, físicas o curriculares… Centradas, sobre todo, en el proyecto docente.
P22: Vale. Bueno, es que en el proyecto docente sería complicado, salvo que tú sepas con antelación que lo vas a tener. Se hace la adecuación sobre la marcha. Si tienes alumnos…te adecuas a ellos. Y, en cuanto a los espacios físicos, eso sí se preocupan…nosotros tenemos aulas que están preparadas, sí, sí, para los alumnos que vienen en silla de ruedas, tienen su espacio y tal, y luego siempre hay alumnos que les llevan al baño, que los traen, que, en ese sentido, yo creo que va funcionando bien. 


Documento:		4. Ciencias Sociales y Jurídicas\P23 CCSS Creencias
Peso:	0
Posición:	60 - 61
Código:	1. Creencias\Adaptaciones curriculares\1.11. Adaptaciones
E: Estupendo. Y, ¿qué opinas sobre la necesidad de realizar adaptaciones en su asignatura para que los estudiantes con discapacidad puedan aprender?
P23: Sí, yo creo que se pueden hacer adaptaciones. Es decir, si la asignatura exige un examen escrito y yo tengo un alumno que no es capaz de hacer, describir... Porque me dijo una el otro día que no podía escribir al mismo ritmo que los demás porque tenía una dislexia o una cosita así... Se lo hago oral. Y lo grabo y luego lo corrijo. Si tengo una alumna que escribir le cuesta mucho, pues que me lo haga en el ordenador y me lo... Yo pienso que sí, que se deben hacer adaptaciones. En ese sentido, yo no sé si tú en tu por tu proyecto lo sabes, pero la facultad está bastante bien. Es decir, tiene las aulas adaptadas, tiene los accesos adaptados... Incluso para el profesorado. Porque tenemos una profesora con discapacidad y tenemos un aula donde da clase ella porque tiene también un problema con la climatización. Entonces claro, tenemos bastante adaptada la facultad en esas cuestiones.


Documento:		4. Ciencias Sociales y Jurídicas\P25 CCSS Creencias
Peso:	0
Posición:	68 - 69
Código:	1. Creencias\Adaptaciones curriculares\1.11. Adaptaciones
E: Perfecto. Bueno, en cuanto a las adaptaciones que ya me has comentado que no has hecho muchas, pero, ¿qué opinas tú sobre la necesidad de realizar adaptaciones en la asignatura para apoyar el aprendizaje de los estudiantes con discapacidad?
P25: Yo pienso que tendría que hacer muchas cosas para adaptarla porque está hecha para chicos y chicas sin discapacidad. Entonces, yo sí que tendría, por ejemplo, si me llega una chica ciega, pues ya tendría que ver cómo lo hago, qué tipo de prácticas harías, etc., es algo que no está pensado. Es que el problema también es que alumnos con discapacidad no vienen, sordos, por ejemplo, no he tenido ninguno, jamás. Y yo pienso, “¿por qué no llegan los sordos a la universidad?”, pues no, pues no llegan. ¿Qué pasa con ellos? ¿se quedan ahí no?
