# Supplementary material for: Is pedagogical training an essential requirement for inclusive education? The case of faculty members in the area of Social and Legal Sciences in Spain
Source: PLoS One. 2021 Jul 2;16(7):e0254250. doi: 10.1371/journal.pone.0254250 (PMC8253417; doi:10.1371/journal.pone.0254250)
Supplement: S1 File — (ZIP) [file pone.0254250.s001.zip › 2.1. DISCAPACIDAD Y TIPOS.rtf]

Documento:		4. Ciencias Sociales y Jurídicas\P1 CCSS Creencias
Peso:	0
Posición:	64 - 65
Código:	2. Conocimientos\Conocimiento general discapacidad\2.1 Discapacidad y tipos
E: Vale. Y pasando a la segunda parte sobre los conocimientos, qué sabes tú sobre la discapacidad.
P1: Pues que existen diferentes tipologías con diferentes necesidades, como tengo el curso del ICE, pues tengo un poco más de información.


Documento:		4. Ciencias Sociales y Jurídicas\P1 CCSS Creencias
Peso:	0
Posición:	74 - 75
Código:	2. Conocimientos\Conocimiento general discapacidad\2.1 Discapacidad y tipos
E: Y qué tipos de discapacidades crees que podemos encontrar en un aula universitaria.
P1: Todas. Lo que pasa es que las visuales son bastante aparentes y las no visuales están escondidas, pero por eso es fundamental que el profesorado exponga que cualquier alumno con cualquier problema lo podemos atender y lo vamos a atender, pero realmente nos podemos encontrar cualquier tipo de discapacidad, seamos realistas.


Documento:		4. Ciencias Sociales y Jurídicas\P2 CCSS Creencias
Peso:	0
Posición:	78 - 79
Código:	2. Conocimientos\Conocimiento general discapacidad\2.1 Discapacidad y tipos
E: ¿Tú qué tipo de discapacidades crees que podemos encontrar en un aula?
P2: Pues por lo que yo estoy viendo creo que cada vez será más frecuente las personas que tengan diagnosticado asperger u otro tipo de personas que pudieran ser bipolares o hiperactivos. Eso sí me he dado cuenta. Gente que tenga algún tipo de trastorno…vamos yo tampoco soy médico ni quiero diagnosticar, pero hay veces que te das cuenta que hay gente que tiene algún tipo de enfermedad mental o lo que fuera. Y después, el tema de las minusvalías físicas. Es las que yo creo que puedan ser más frecuentes.


Documento:		4. Ciencias Sociales y Jurídicas\P3 CSS Creencias
Peso:	0
Posición:	72 - 73
Código:	2. Conocimientos\Conocimiento general discapacidad\2.1 Discapacidad y tipos
E: Ya me has comentado algo pero, ¿cuántas discapacidades crees que nos podríamos encontrar en un aula?
P3: Pues, nos podemos encontrar varios tipos de discapacidad como física, desde el punto de vista de movilidad, deficiencia visual, y deficiencia o discapacidades psicológicas. 


Documento:		4. Ciencias Sociales y Jurídicas\P4 CCSS Creencias
Peso:	0
Posición:	66 - 67
Código:	2. Conocimientos\Conocimiento general discapacidad\2.1 Discapacidad y tipos
E: Antes hemos hablado sobre las ideas que se te venían a la cabeza cuando oías la palabra discapacidad, ahora te pregunto, qué sabes sobre discapacidad.
P4: Bueno, yo sé que hay muchos tipos diferentes, que es una característica que tienen determinadas personas que les hace, pues tener unas necesidades diversas, ¿no? Y que hay que prestar atención a qué tipo de discapacidad, porque son muy diferentes. Eso es lo que creo que es más importante.


Documento:		4. Ciencias Sociales y Jurídicas\P5 CSS Creencias
Peso:	0
Posición:	11 - 14
Código:	2. Conocimientos\Conocimiento general discapacidad\2.1 Discapacidad y tipos
Pero, no sé, había una alumna que es que ella tenía dificultades en el habla, no sé si eso es una discapacidad.
E: Sí.
P5: Provocado por una operación del cerebro que, además, tenía pendiente otra… Y luego, pues esa alumna que se puso en contacto conmigo hace un par de cursos para decirme que tenía una discapacidad, que yo, de verdad, no supe…ni me lo contó, ni yo, por supuesto, me di cuenta, terminó el curso y no supe qué discapacidad tenía esta alumna, vamos. ¿La tartamudez es una discapacidad?
E: Es una dificultad del lenguaje, me imagino que sí.


Documento:		4. Ciencias Sociales y Jurídicas\P5 CSS Creencias
Peso:	0
Posición:	78 - 79
Código:	2. Conocimientos\Conocimiento general discapacidad\2.1 Discapacidad y tipos
E: Muy bien. Vamos a pasar al segundo bloque de preguntas de entrevistas que nosotros llamamos de conocimientos, ¿no? Saber qué cosas conoces tú sobre discapacidad, educación inclusiva, etc. ¿Qué sabes sobre discapacidad?
P5: Nada, nada, no te puedo decir…incluso al principio te dije “¿la tartamudez es una discapacidad?”. Tendría problemas para definir qué es discapacidad, por no decir ninguna barbaridad. Ahora yo he escuchado, cómo se llama…que las discapacidades son capacidades diferenciadas, ¿no? Algo así, ¿no? Entonces, dices “¿estoy diciendo alguna chorrada o bestialidad diciendo que alguien tiene una discapacidad?”. No lo sé. O sea, que poco, de discapacidad, poco.


Documento:		4. Ciencias Sociales y Jurídicas\P5 CSS Creencias
Peso:	0
Posición:	90 - 91
Código:	2. Conocimientos\Conocimiento general discapacidad\2.1 Discapacidad y tipos
E: Y qué tipos de discapacidades crees que nos podemos encontrar en un aula en la universidad.
P5: Pues, visto lo visto, de todo. Aquí hay alumnos y profesores con movilidad reducida, que van en silla de ruedas, dificultades de aprendizaje, no sé si se puede decir severo, pero sí importante, gente que lleva cuatro o cinco años con la asignatura y estudia y se lo curra y no… ¿No?


Documento:		4. Ciencias Sociales y Jurídicas\P6 CCSS Creencias
Peso:	0
Posición:	58 - 59
Código:	2. Conocimientos\Conocimiento general discapacidad\2.1 Discapacidad y tipos
E: Claro. Bueno, pasamos al segundo bloque de la entrevista que es al que nosotros llamamos de conocimientos, que es para, bueno, saber qué conocimientos tienes tú sobre educación inclusiva, discapacidad… ¿Qué sabes o qué conoces sobre la discapacidad?
P6: No sé nada, realmente, lo único que puedo decirte es que creo que toda discapacidad es superable, sin duda alguna, si la sitúas en el contexto adecuado, ¿no? Y, bueno, como te he comentado, creo que es un concepto muy amplio que abarca muchas circunstancias, que habría que analizarlas una a una y no hablar de esta manera tan genérica del concepto de discapacidad. Y que, bueno, que quiero pensar que solamente se convierte en una dificultad añadida a la hora de poder enfrentarte a cualquier proceso de aprendizaje.


Documento:		4. Ciencias Sociales y Jurídicas\P6 CCSS Creencias
Peso:	0
Posición:	68 - 69
Código:	2. Conocimientos\Conocimiento general discapacidad\2.1 Discapacidad y tipos
E: Estupendo. Y, ¿qué tipo de discapacidades crees que nos podemos encontrar en las aulas universitarias?
P6: Pues aquí en la universidad sé que tenemos alumnos minusválidos, sé que tenemos alumnos ciegos porque me los he encontrado alguna vez, y claro, discapacidades que no sean físicas ya te digo que no me he encontrado ni tengo conciencia de…claro, es que como no son visibles a la percepción simple, pues no soy consciente, pero me imagino que habrá discapacidades en el sentido de eso, de que quizá te cueste más trabajo entender las cosas o más tiempo… Supongo que seguro, pero no soy consciente de ello.


Documento:		4. Ciencias Sociales y Jurídicas\P8 CCSS Creencias
Peso:	0
Posición:	71 - 75
Código:	2. Conocimientos\Conocimiento general discapacidad\2.1 Discapacidad y tipos
P8: Era una cosa increíble, o sea, estoy como flipada, ¿no?
E: Normalmente cuando no toman la medicación se nota mucho, se descompensa. Todo lo mal que le pueda ir es cuando no toman la medicación. Claro y hasta que vuelven a medicarse no vuelven otra vez a tener cierta objetividad sobre la vida y sobre la situación.
P8: Claro es que me daba la sensación de que cuando no se medicaba era todo muy, muy negativo, ¿no? Porque era “esto no sé para qué si no voy a encontrar trabajo en la vida en cuanto ponga esquizofrénico, patada”. Era eso lo que transmitía, y yo decía “qué quieres que te diga, tú sabes lo que tienes que hacer”. Eso es igual que la tensión alta. Yo tengo la tensión alta y me tengo que tomar todos los días dos pastillas, pues ya está.
E: Normalizar quizás eso también ayudaba, ¿no?
P8: Sí, yo creo que habar con naturalidad las cosas.


Documento:		4. Ciencias Sociales y Jurídicas\P8 CCSS Creencias
Peso:	0
Posición:	163 - 169
Código:	2. Conocimientos\Conocimiento general discapacidad\2.1 Discapacidad y tipos
 Te voy a contar otra cosa, no es de discapacidad, pero a ver, yo he tenido una niña con depresión de enfermedad. No es la tontería que decimos “no, está deprimida”.
E: Crónica, ¿no?
P8: Una depresión medicada, ¿no? No quería que se supiera en clase hasta el punto de que, en algún momento, alguien, que es una frase tonta “que estás loca”, dirigida a ella, era como…
E: Una ofensa impresionante.
P8: Una ofensa y no lo sabía articular. Te digo, o sea, porque a ver, a lo mejor es que yo soy muy madre…no es que sea buena docente, que no tiene nada que ver, soy bastante receptiva con los alumnos. No sé por qué se crea eso, yo creo que es porque les doy facilidades de comunicación, pero, para que te hagas una idea, el año pasado se suicidó la madre de un estudiante mío. La niña vino a contarme, “mi madre se ha suicidado, estoy fatal” y te digo, no era problema de este chico y ahí le dije “ningún problema”. Ahí estábamos el día 22, con la lotería puesta en internet por si nos tocaba el gordo que nos íbamos a ir a emborrachar juntas, estudiando el examen porque le iba a examinar una semana después, es decir, yo no puedo ser insensible a una situación de ese tipo. No puedo, o sea, y me pidió por favor que no comentara nada en clase. Solo se lo había dicho a una persona porque su madre era una persona depresiva y estaba en tratamiento y se había suicidado. Tenía una depresión que le llevó a suicidarse. Entonces, me decía “es que yo sé que algunos se lo toman a cachondeo y me dicen la hija de la loca, y tú que siempre estás triste vas a hacer lo mismo”, o sea… por eso te digo que me parece que la calidad humana y esa colaboración entre compañeros, ese compañerismo en el fondo, falta.
E: Falta…
P8: Falta muchísimo.


Documento:		4. Ciencias Sociales y Jurídicas\P9 CCSS Creencias
Peso:	0
Posición:	78 - 81
Código:	2. Conocimientos\Conocimiento general discapacidad\2.1 Discapacidad y tipos
E: ¿Y tú qué sabes de discapacidad? 
P9: Yo, nada.
E: Nada, o sea que nunca te has formado ni sobre educación inclusiva…
P9: No, nunca. Tampoco. O sea, yo el único curso que he ido un poco relacionado con el tema de la discapacidad es este del lenguaje de signos y un poco porque, como a mí me gusta mucho el tema de la comunicación, y con las asignaturas que tengo pues dije “oye, pues es otra forma de comunicarte”, ¿no? Ver cómo se comunican ellos y cómo influyen el que tengas mermadas unas determinadas capacidades como pueda ser la visión o el oído y tal. Es lo único y fue como tres tardes, unas tres o cuatro horas cada tarde, o sea, que no fue más. No, no, no tengo ningún tipo de formación.


Documento:		4. Ciencias Sociales y Jurídicas\P10 CCSS Creencias
Peso:	0
Posición:	88 - 91
Código:	2. Conocimientos\Conocimiento general discapacidad\2.1 Discapacidad y tipos
E: Sí, sí, sí. Vamos a hacer ya la última parte de la entrevista, ¿vale? Entonces, esta es sobre conocimiento. Sobre lo que conoces, sobre lo que sabes, sobre... Vamos a ver, lo primero es: ¿qué sabes sobre discapacidad? Así un poco...
P10: Madre mía. ¿Qué sé sobre discapacidad? Vamos a ver, pues saber no sé nada. No tengo ni puñetera idea de nada, pero entiendo yo que podemos estar hablando de personas que se salen, en algunos aspectos, de los estándares más al uso. Yo que sé, es decir, pues por una patología o por algún tipo de problema del tipo que sea. Patología del tipo que sea, es decir: física, psíquica... No lo sé. O sea, que digamos con respecto a los estándares... Con muchísimo miedo, por eso los estándares yo creo que, como te he dicho antes, creo que el mundo es poliédrico y, por lo tanto, es decir “yo es que soy normal” ¿Tú normal? ¿De qué dimensión me estás hablando?
E: ¿Qué es lo normal aquí?
P10: ¿Sabes? Que dices, pues sí. Lo que pasa es que hay... Que yo creo que esto acaba definiéndose algo que no se aclara muy bien nunca por temas culturales, sociológicos que se crea pues una imagen que yo creo que es diferente para cada uno de lo que es “normal”. Entonces, cuando de eso se sale y está identificado... Mira, es que “está discapacitado. Es que le falta un brazo”. Pues hombre, pues sí. A la inmensa mayoría de la gente no le falta un brazo. Pues evidentemente. Le falta un brazo, pues claro. No sé, por decir una cosa y otras historias... Entonces para mí, discapacidad en ese sentido es algo que limita tus capacidades. Respecto a eso que no sé muy bien cómo definirlo. Entonces... Y que también si hurgamos mucho seguro que nos sacan alguna discapacidad a la mayoría en algún aspecto. Que a lo mejor aquí no es muy relevante y a lo mejor en una sociedad de cazadores seríamos unos gilipollas perdidos porque no servimos para nada porque claro... Entonces, supongo que es una cosa también que hay que poner en contexto histórico... No sé. Es lo que pienso.


Documento:		4. Ciencias Sociales y Jurídicas\P11 CCSS Creencias
Peso:	0
Posición:	77 - 78
Código:	2. Conocimientos\Conocimiento general discapacidad\2.1 Discapacidad y tipos
E: Vale. Y qué tipos de discapacidades crees que podemos encontrarnos en un aula universitaria.
P11: Pues yo por mi casuística he visto distintas, tanto físicas como psíquicas. No sé si llamarlas físicas o mentales, pero claro, no es igual una persona con una deficiencia visual, que lógicamente es un problema físico, que una persona con un síndrome de Asperger, que es un tema no sé si psicológico o mental, cerebral… No sé cómo denominarlo. Entonces, claro, las barreras físicas se tratan de una manera y las mentales de otra.


Documento:		4. Ciencias Sociales y Jurídicas\P12 CCSS Creencias
Peso:	0
Posición:	86 - 93
Código:	2. Conocimientos\Conocimiento general discapacidad\2.1 Discapacidad y tipos
E: ¿Qué sabes sobre la discapacidad?
P12: Yo poca cosa. Lo que veo en mi entorno es lo que sé. 
E: ¿Qué es la discapacidad? Bueno, esto ya lo has dicho antes. 
P12: Sí, ya lo he comentado.
E: ¿Qué tipos de discapacidades conoces o sabes que existen?
P12: Discapacidad intelectual, motora y visual. Éstas son las tres que he visto.
E: ¿Conoces si existen más discapacidades a parte de estas tres que has descrito?
P12: Sí, discapacidad auditiva…yo que sé, hay más, aunque yo no he trabajado con ellos.


Documento:		4. Ciencias Sociales y Jurídicas\P14 CCSS Creencias
Peso:	0
Posición:	20 - 21
Código:	2. Conocimientos\Conocimiento general discapacidad\2.1 Discapacidad y tipos
E: Vale. Pasamos ahora a las creencias, el bloque que te comentaba antes. Entonces, si hablamos sobre las concepciones de discapacidad, cuando hablamos de discapacidad, ¿qué ideas se te vienen a la cabeza?
P14: Claro, a ver, de discapacidad, yo lo que entendía, era, por ejemplo, pues esta persona que le faltan dedos en la mano, ¿no? Entonces, entiendo que por esa circunstancia necesita alguna adaptación o tiene unas necesidades educativas un tanto especiales para garantizar que tenga las mismas oportunidades que el resto, ¿no? Entonces, es, digamos, compensar una discapacidad física, en este caso, pues para que tenga las mismas oportunidades que los demás.


Documento:		4. Ciencias Sociales y Jurídicas\P14 CCSS Creencias
Peso:	0
Posición:	66 - 67
Código:	2. Conocimientos\Conocimiento general discapacidad\2.1 Discapacidad y tipos
E: Vale. Y, ahora ya vamos terminando, vamos con la parte un poco de conocimientos. Antes me has dicho lo que te viene a la cabeza cuando te digo la palabra discapacidad, pero a nivel ya más de conceptos, ¿tú qué sabes sobre discapacidad?
P14: Pues, nunca he tenido ninguna experiencia cercana en mi familia y entonces, conceptualmente, no podría definirte creo que, correctamente qué es discapacidad y no tengo una definición.


Documento:		4. Ciencias Sociales y Jurídicas\P14 CCSS Creencias
Peso:	0
Posición:	74 - 77
Código:	2. Conocimientos\Conocimiento general discapacidad\2.1 Discapacidad y tipos
E: Y, ¿qué tipo de discapacidades crees que se pueden encontrar en un aula universitaria? Es decir, por ejemplo, ¿crees que podríamos encontrarnos alumnos con parálisis cerebral? Sabemos que lo que son discapacidades físicas sí llegan, ¿algún tipo más? Ya no que conozcas, sino que deduzcas por cómo funciona la universidad que puedan llegar o que sea posible. 
P14: Sí. Pues, desde estudiantes con movilidad reducida o que les falte algún…que no puedan ver, o que no puedan oír… ¿no? Entiendo que eso un tipo de discapacidad. Luego, ansiedad, porque sí es verdad que algún año he recibido recomendaciones de la USE para alumnado que tenía ansiedad, pero tampoco tengo muy claro qué entendemos por discapacidad o no, pero entiendo que discapacidad física o que tenga movilidad reducida, o que le falte algún miembro o algún sentido que…que no vea o no escuche o lo que sea y no sé qué otros tipos de discapacidad…
E: Autismo, por ejemplo…
P14: Autismo, el otro día me comentaba una compañera que tenía un estudiante con Asperger, ¿no? y que se había dormido en clase o que no atendía y estaba jugando al ordenador… Pues este tipo de casos intuyo que pueden llegar, pero ya te digo, no he tenido en el aula estas experiencias.


Documento:		4. Ciencias Sociales y Jurídicas\P15 CCSS Creencias
Peso:	0
Posición:	82 - 83
Código:	2. Conocimientos\Conocimiento general discapacidad\2.1 Discapacidad y tipos
E: Vale. Y, con respecto a tus conocimientos acerca de la discapacidad, ¿qué sabes sobre discapacidad?
P15: Pues sé muchas cosas y sé pocas cosas. No sé, lo que uno medianamente se entera a través de los medios. No sé, no sabría tampoco cómo concretar.


Documento:		4. Ciencias Sociales y Jurídicas\P15 CCSS Creencias
Peso:	0
Posición:	102 - 103
Código:	2. Conocimientos\Conocimiento general discapacidad\2.1 Discapacidad y tipos
E: Vale. ¿Qué tipos de discapacidades crees que podemos encontrarnos en un aula universitaria?
P15: Hombre, supongo que como poder, de todo lo más diverso que haya, como poder, sí.


Documento:		4. Ciencias Sociales y Jurídicas\P16 CCSS Creencias
Peso:	0
Posición:	54 - 55
Código:	2. Conocimientos\Conocimiento general discapacidad\2.1 Discapacidad y tipos
E: Con respecto a los tipos de discapacidad, ya me has mencionado la discapacidad sensorial, la física, ¿conoces otros tipos de discapacidad? 
P16: La psíquica.


Documento:		4. Ciencias Sociales y Jurídicas\P18 CCSS Creencias
Peso:	0
Posición:	80 - 81
Código:	2. Conocimientos\Conocimiento general discapacidad\2.1 Discapacidad y tipos
E: Y, ¿qué conoces sobre la discapacidad?, por ejemplo.
P18: Lo que estoy dando yo ahora mismo en la asignatura, en el acceso al empleo, en las cuotas de empleo que existen tanto en la administración como en determinadas empresas, tienen puestos reservados a ellos, que hay distintos tipos de discapacidad…


Documento:		4. Ciencias Sociales y Jurídicas\P19 CCSS Creencias
Peso:	0
Posición:	60 - 61
Código:	2. Conocimientos\Conocimiento general discapacidad\2.1 Discapacidad y tipos
E: Bueno, ahora vamos a pasar a los conocimientos que tienes sobre la discapacidad ¿Qué sabes sobre la discapacidad?
P19: Buf, nada, nada. Yo solo sé el tema de la incapacitación judicial, porque cuando aquí me vienen al despacho casos para que a alguien le den la incapacitación judicial, yo les digo que deben traer el Informe médico, quiénes son los parientes cercanos, a quien quieren nombrar tutor, y poco más. No sé.


Documento:		4. Ciencias Sociales y Jurídicas\P19 CCSS Creencias
Peso:	0
Posición:	72 - 73
Código:	2. Conocimientos\Conocimiento general discapacidad\2.1 Discapacidad y tipos
E: ¿Qué tipos de discapacidades crees que podemos encontrar en un aula universitaria?
P19: Pues cualquier tipo de discapacidad, quizás los alumnos que más nos encontremos sean los alumnos con discapacidades sensoriales, como sordomudos o ciegos, o discapacidades físicas, que vayan en silla de ruedas o en muletas, pero también nos podemos encontrar discapacidades intelectuales, o dificultades de aprendizaje, como es el caso de Alberto, no sé, creo que cualquiera. Bueno, no creo que nos encontremos discapacidad intelectual severa, te lo digo porque conozco, con nombres y apellidos, a un chico con Síndrome de Down, que no creo que pueda estudiar una carrera, una discapacidad muy profunda no creo, pero al final, la universidad es un reflejo de la sociedad, por lo que nos podemos encontrar cualquier tipo de discapacidad.


Documento:		4. Ciencias Sociales y Jurídicas\P20 CCSS Creencias
Peso:	0
Posición:	26 - 27
Código:	2. Conocimientos\Conocimiento general discapacidad\2.1 Discapacidad y tipos
E: Claro. Y bueno, pasando a las creencias que tienes sobre discapacidad. Cuando hablamos de discapacidad, ¿a ti qué ideas se te vienen a la cabeza?
P20: Pues mira, la discapacidad para mí, sinceramente, es que una persona tiene algún tipo de dificultad para desarrollar sus habilidades, pero no mucho más…y desde el punto de vista jurídico, que sí es verdad que nosotros lo tenemos como más interiorizado, ¿no? Que se reconoce la discapacidad cuando tienes el 33% o más de discapacidad, de que no puedes desarrollar unas labores igual que el resto de los alumnos, vamos, esa es mi idea, no es más.


Documento:		4. Ciencias Sociales y Jurídicas\P20 CCSS Creencias
Peso:	0
Posición:	70 - 71
Código:	2. Conocimientos\Conocimiento general discapacidad\2.1 Discapacidad y tipos
E: Y pasando a los conocimientos acerca de la discapacidad, qué sabes sobre discapacidad.
P20: Pues mira, lo que sé es un poco lo que te comentaba antes desde el punto de vista legal. Yo me preocupo de lo de capacidad legal, capacidad jurídica, del desarrollo de cuándo se declara una persona en situación de discapacidad… O sea, mis conocimientos son más de tipo jurídico, la verdad. Te podría decir los cambios hasta en el lenguaje, que hemos pasado de la ley con respecto a minusválidos, que eso ya hace años que se cambió, pero bueno, vamos un poquito cambiando. La palabra incluso “discapacidad”, que ya intenta cambiarse por otro tipo de lenguaje menos violento incluso, ¿no? Porque discapacidad es como que no tuvieras capacidad. Y no, tienes otro tipo de capacidades. Y bueno, pues ese tipo de conocimiento sí que lo tengo, pero jurídico, no de otro tipo. O, por ejemplo, los cupos para el empleo público o en la legislación de contratos… Pues bueno, ese tipo de conocimientos son los que tengo.


Documento:		4. Ciencias Sociales y Jurídicas\P20 CCSS Creencias
Peso:	0
Posición:	80 - 81
Código:	2. Conocimientos\Conocimiento general discapacidad\2.1 Discapacidad y tipos
E: Muy bien. Y esto ya me lo has comentado, que conoces los servicios y recursos que tienen, ¿qué tipos de discapacidades crees que podríamos encontrarnos en un aula universitaria?
P20: Pues mira, yo creo que, de todo, porque nosotros nos hemos encontrado de todo, durante los años de docencia, excepto el caso este de no movilidad, todo lo demás nos lo hemos encontrado: personas con discapacidad auditiva, visual, de movimientos también, de ciertas enfermedades de tipo esquizofrenia o… Nos hemos encontrado un poco de todo. Discapacidades intelectuales… O sea, de todo y no ha habido problema.


Documento:		4. Ciencias Sociales y Jurídicas\P21 CCSS Creencias
Peso:	0
Posición:	136 - 145
Código:	2. Conocimientos\Conocimiento general discapacidad\2.1 Discapacidad y tipos
E: Y pasamos al segundo bloque de la entrevista. Como conocimiento general, ya no es lo que piensa, sino, ¿qué sabe sobre la discapacidad?
P21: ¿Qué sé? Es que, desde el punto de vista cuantitativo, no sabría decirte. Porque no es un tema que yo haya abordado específicamente como estudio para saber de dónde he llegado hasta dónde he llegado. Yo tengo una hermana con parálisis, con lo cual, he vivido con la discapacidad...
E: Toda la vida.
P21: Desde niño. Y bueno, por las circunstancias, mi madre era “tú te encargas de tu hermana”. Yo he sido el encargado de mi hermana porque había que ayudarla a andar, la tenías que llevar, la tenías que traer. La discapacidad ha formado para de...
E: De su vida.
P21: De mi vida. Quizás también en ese sentido... Entonces, sé de las dificultades, sé de... Pero no es lo mismo, ¿no? Y afortunadamente yo he vivido en un entorno familiar en el que “eres coja, pero no eres tonta”. Con lo cual, “venga, ¿no?” y se la atendía en la medida en la que ves que tú tienes un límite. Si tú no puedes correr, no puedes correr. Pero esto lo puedes hacer y esto lo haces, ¿no?
E: De su forma.
P21: Claro, y es que no sabría decirte qué sé en el sentido de...hombre, es un tema que me interesa y sé lo que puede saber cualquier persona. Que me gusta pensar que soy una persona informada, ¿no? Y me interesa el tema. Es un tema al que presto atención como en líneas generales a…yo siempre digo, por hablar de las secciones de un periódico, ¿no?  Yo nunca leo las páginas de deporte, pero siempre leo las páginas de sociedad. No de sociedad rosa, sino de los temas... Porque me interesan los temas sociales y ahí suele estar incluido...
E: La discapacidad.
P21: Todo este tema, ¿no?


Documento:		4. Ciencias Sociales y Jurídicas\P21 CCSS Creencias
Peso:	0
Posición:	162 - 165
Código:	2. Conocimientos\Conocimiento general discapacidad\2.1 Discapacidad y tipos
E: Y, ¿qué tipo de discapacidades cree que podemos encontrar en el aula?
P21: Hombre, yo aquí en este sentido, sí es verdad, aunque conozco como decía, no por estudios, pero sí por estar informado, pues de esos casos, ¿no? de discapacidades mentales que...lo que pasa que también es un tema que...cuando la gente te dice “ay mira, un síndrome de Down que ha hecho una carrera universitaria”. Pero es que del síndrome de Down hay grados y grados, es como la discapacidad física. No es lo mismo una cojera, que el caso de Rafa, ¿no? Entonces, hablar de tipos de discapacidad...yo con las que me he encontrado han sido siempre discapacidades físicas. De diferentes ámbitos, pero no sé si hay casos muy concretos de discapacidades en ese sentido. Volvemos a lo mismo. Porque no es lo mismo la enseñanza obligatoria o el periodo de inclusión...o sea, tú a un niño no puedes dejar de llevarlo al colegio en edad escolar porque tenga una discapacidad mental y ya ahí es donde se debe fomentar la inclusión, que es buena para que los niños se relacionen con personas de todo tipo y tal. Pero, claro, llegará un momento en el que, si tú no tienes capacidad mental para estructurar pensamiento y tal, para hacer en un momento determinado una carrera universitaria, no llegarás. Yo todo lo que he tenido han sido alumnos con discapacidades físicas. Desconozco si hay grados de discapacidad mental, cómo se afrontan. Yo siempre digo lo mismo, yo... Hay una anécdota de un día que entró en el ascensor... Entrábamos varios, ya había unos alumnos dentro y uno de los que entraba era una alumna que venía de sacar un libro de la biblioteca. Y le dice la compañera “mira, la pelota, lo habrá sacado a ver si le ponen...” y la otra le responde de una manera muy natural “no, que, además, este libro es muy interesante”. Y salta la otra y dice “pues yo te puedo asegurar que llevo aprobadas todas las asignaturas y no he necesitado leerme ningún libro. Vamos, yo no me leo un libro...”. Presumiendo de...
E: De una gran carencia.
P21: De que ella no necesita leerse un libro. Y en ese momento, de verdad, yo digo que tendríamos que tener un sistema en el que eso quedara registrado y ese alumno fuera expulsado de la universidad. Porque eso sí que son carencias. Que van buscando por allí, que van buscando el aprobado, que van buscando cómo aprueban y he aprobado y fuera.


Documento:		4. Ciencias Sociales y Jurídicas\P22 CCSS Creencias
Peso:	0
Posición:	88 - 89
Código:	2. Conocimientos\Conocimiento general discapacidad\2.1 Discapacidad y tipos
E: Claro. Vamos a la segunda parte de la entrevista y ya terminamos con ella ¿Qué sabe sobre la discapacidad?, ¿qué conocimientos tiene?
P22: No muchos, confieso, no muchos. Tengo la idea de que son distintas personas, de diferentes tipos, físicas, psíquicas, y bueno, poco más, la verdad. Que hay esfuerzos, que, en los medios de comunicación, pues tenemos también unas determinadas recomendaciones de tratamiento, que ellos se sienten discriminados en los medios, pero como todos los colectivos minoritarios, tengo que decirlo, es decir, que no es una cosa solamente de discapacitados, ¿no?


Documento:		4. Ciencias Sociales y Jurídicas\P22 CCSS Creencias
Peso:	0
Posición:	110 - 111
Código:	2. Conocimientos\Conocimiento general discapacidad\2.1 Discapacidad y tipos
E: ¿Qué tipos de discapacidades cree que podemos encontrarnos en el aula?
P22: Bueno, pues física normalmente, porque claro, estamos en la universidad y, difícilmente tendremos una deficiencia psíquica aquí. Ya te digo, ciegos, y alumnos paralíticos…ese tipo de cosas es lo que se suele dar.


Documento:		4. Ciencias Sociales y Jurídicas\P23 CCSS Creencias
Peso:	0
Posición:	76 - 77
Código:	2. Conocimientos\Conocimiento general discapacidad\2.1 Discapacidad y tipos
E: Y el segundo bloque, centrado en el conocimiento. ¿Qué sabe sobre la discapacidad?
P23: Sobre la discapacidad sé poco porque claro, yo no estoy formada en temas de discapacidad. Sé lo que tengo al alcance por familiares, compañeros, amigos y alumnos. Nada más.


Documento:		4. Ciencias Sociales y Jurídicas\P23 CCSS Creencias
Peso:	0
Posición:	104 - 109
Código:	2. Conocimientos\Conocimiento general discapacidad\2.1 Discapacidad y tipos
E: Y, ¿qué tipo de discapacidades cree que podemos encontrar en el aula?
P23: En el aula... Pues yo he encontrado discapacidad visual varias veces, he encontrado discapacidad motriz varias veces y la discapacidad degenerativa que tenía Rafa, que no me la sé.
E: Sí, la enfermedad de Duchenne. Esa también es física-motriz.
P23: Física-motriz y visual. No me ha tocado encontrarme con discapacidad...
E: Auditiva.
P23: Auditiva no. Aquí en periodismo no. Pero yo me imagino que podrás encontrar también, claro.


Documento:		4. Ciencias Sociales y Jurídicas\P24 CCSS Creencias
Peso:	0
Posición:	29 - 29
Código:	2. Conocimientos\Conocimiento general discapacidad\2.1 Discapacidad y tipos
Y otro perfil invisibilizado son los estudiantes con diversidad inmigrante, extranjeros, que tienen dos o tres estigmatizaciones, por su diversidad funcional, por ser inmigrante o extranjero…y en el caso de esta chica con tartamudez, era inmigrante y tuvo la tartamudez durante la migración.


Documento:		4. Ciencias Sociales y Jurídicas\P24 CCSS Creencias
Peso:	0
Posición:	39 - 39
Código:	2. Conocimientos\Conocimiento general discapacidad\2.1 Discapacidad y tipos
Porque normalmente, si ya a nosotros, cuando tú me has preguntado al principio que qué le podía costar a los estudiantes de mi asignatura y nos cuesta, decir nuestras fortalezas, a la propia persona con diversidad, les cuesta muchísimo más, porque como la tienen tan machacada…y eso es algo estructural, les cuesta vérselas en ese sentido. 


Documento:		4. Ciencias Sociales y Jurídicas\P24 CCSS Creencias
Peso:	0
Posición:	39 - 39
Código:	2. Conocimientos\Conocimiento general discapacidad\2.1 Discapacidad y tipos
Son personas como una más, pero con unas capacidades diferentes de superación, en positivo, no en negativo como normalmente las vemos.


Documento:		4. Ciencias Sociales y Jurídicas\P25 CCSS Creencias
Peso:	0
Posición:	27 - 27
Código:	2. Conocimientos\Conocimiento general discapacidad\2.1 Discapacidad y tipos
P25: Hombre, también, he tenido alumnado con problemas de salud mental, aunque no esté catalogado como una discapacidad física o sensorial.


Documento:		4. Ciencias Sociales y Jurídicas\P25 CCSS Creencias
Peso:	0
Posición:	55 - 55
Código:	2. Conocimientos\Conocimiento general discapacidad\2.1 Discapacidad y tipos
P25: Hombre, para personas con problemas de salud mental sí, porque ahí hay cosas que a uno se le escapan. A mí…claro una persona que de pronto empieza a ilusionar, tú tienes que saber reaccionar ante eso, ¿no? y saber contener el grupo ante esa situación. Para eso sí, pero para las personas con una discapacidad física, o sensorial, o un Autismo, por ejemplo, pues mira, saber un poco de qué va el asunto, pero tratarlo con normalidad, creo yo que es lo suyo. No hace falta marcar tanto la diferencia, ya este mundo la marca.


Documento:		4. Ciencias Sociales y Jurídicas\P25 CCSS Creencias
Peso:	0
Posición:	92 - 95
Código:	2. Conocimientos\Conocimiento general discapacidad\2.1 Discapacidad y tipos
E: Entrando ahora en la parte de conocimientos sobre discapacidad. Te he preguntado antes, sobre el concepto de discapacidad, sobre qué ideas se te viene a la cabeza, y ahora te pregunto, qué sabes sobre la discapacidad, qué crees que es, si sabes definirlo, si sabes algo.
P25: A ver, yo nunca tuve que ponerme a trabajar esta materia, sé lo que una aprende. Sé que la discapacidad es un grado importante en la vida, entonces, sé que es un obstáculo para poder ser uno; pero puede ser también todo lo contrario, dependiendo de la suerte que tengas en la vida, de la familia que te toque, el contexto en el que vivas, el barrio en el que estés, las oportunidades que se te ofrezcan, entonces, yo he ido viendo que la discapacidad es un grado de dependencia, es decir, no ser capaz  de ser totalmente autónomo y estar trabajando para poder serlo. Pero claro, a veces se puede y otras no se puede; hay personas con discapacidades que nunca van a poder ser como los otros y se les dice lo contrario, y a mí, eso me da mucho coraje. Yo tengo una sobrina con Síndrome de Down profunda, muy profunda, y mi  primo hermano cuando nace se pone como loco a intentar que la niña se rehabilite como fuera, ¿no? terapias físicas, estimulación continua, el fundó un asociación de padres, él fue quien la lideró. Pero, su niña tiene una discapacidad muy profunda, su hija estaba en un centro normalizado porque él estaba totalmente en contra de los centros especiales y ahora está en un centro especial porque él ha comprendido que su hija no va a hablar con fluidez jamás. Entonces, me parece a mí que eso es también importante, no se le puede decir a la gente continuamente ese mensaje porque es engañoso. El típico “si se quiere, se puede”, pues no, a mí me parece que hay grados de discapacidad que a lo mejor sí, pero hay que trabajar con los chavales y con las chavalas la aceptación de esa discapacidad y que no vas a ser como los otros.
E: Sí, hay que ser un  poco más realista, ¿no?
P25: Me parece a mí.


Documento:		4. Ciencias Sociales y Jurídicas\P25 CCSS Creencias
Peso:	0
Posición:	96 - 97
Código:	2. Conocimientos\Conocimiento general discapacidad\2.1 Discapacidad y tipos
E: ¿Qué barreras crees que encuentran los estudiantes cuando están estudiando en la universidad?
P25: Yo creo que los estudiantes se encuentran con la barrera de lo que es el contenido de la materia. Que el profesor se plantea como hemos dicho antes, el trabajo de adaptación en muchos casos o el chaval se va a encontrar con serias dificultades, ¿no? Pero también, depende de la discapacidad que tenga, si tú tienes una discapacidad física, pero intelectualmente estás bien, por qué vas a tener algún problema, ¿no? Ahora, si eres sordo, si no ves, si tienes un nivel…los autistas son súper inteligentes, están y no están, pero cuando están sacan jugo de dónde no sacan los otros, ven lo que los otros no ven. Entonces, las barreras, pues dependerán del problema que tenga el chico o la chica, ¿no?


Documento:		4. Ciencias Sociales y Jurídicas\P25 CCSS Creencias
Peso:	0
Posición:	110 - 111
Código:	2. Conocimientos\Conocimiento general discapacidad\2.1 Discapacidad y tipos
E: ¿Qué tipos de discapacidades crees que puedes encontrar en un momento determinado en el aula?
P25: Pues puedo encontrar pues eso, desde discapacidades sensoriales que ya te he comentado, hasta discapacidades físicas importantes. Eso es lo que yo creo que me puede venir a mí, sinceramente.


Documento:		4. Ciencias Sociales y Jurídicas\P25 CCSS Creencias
Peso:	0
Posición:	130 - 139
Código:	2. Conocimientos\Conocimiento general discapacidad\2.1 Discapacidad y tipos
E: ¿Alguna otra cuestión o información que quieras añadir?
P25: No tengo ni idea ahora mismo. ¿Sabes a quién tengo continuamente en mi cabeza, ahora mismo? pues, este chico que es Síndrome de Down que ha hecho magisterio, que ha sido actor…pues el otro día salió en la televisión…ha hecho otra carrera también, que es simpatiquísimo. Su hermano tiene una carrera y él dijo “pues si tú tienes, yo también tengo”. Y yo me planteo, este chico y con esta mente y sinceramente, he pensado a veces, ¿a este chico se le ha regalado? No vamos a negar, que una siempre tiene prejuicios. Mira, otro elemento, trabajar los prejuicios.
E: No creo que le hayan regalado nada. Hay muchos Síndrome de Down que han llegado a la universidad. Hay muchos profesores y maestros con Síndrome de Down.
P25: Fíjate, pues yo no lo he visto en la vida. A lo mejor, estoy muy limitada, ¿eh?
E: De hecho, hace poco hubo una polémica sobre una chica con Síndrome de Down, que sacó su carrera, sacó su magisterio y estaba dando clases en una escuela de profesora. Y claro, ya surgió la polémica de los padres que si no estaba capacitada para dar clases y era una profesora excelente.
P25: La verdad, es que lo diferente y lo distinto es necesario.
E: Y, profesoras y profesores universitarios doctores con parálisis cerebral.
P25: Ahí, veo yo otra cosa diferente. Yo conozco a muchos chicos y chicas con parálisis cerebral, hijos de amigos míos, y los veo tan inteligentes...claro, pero con lo del Síndrome de Down, lo que yo tengo es pura ignorancia. Y sigo pensado, que me tira mucho mi experiencia.
E: También,  el Síndrome de Down, varía mucho según el grado.
P25: Claro, es que nuestra chica tiene un grado muy profundo, y ya tiene 24 años y habla muy poquito. Ella está en un estadio básico, muy básico.
