# Supplementary material for: Is pedagogical training an essential requirement for inclusive education? The case of faculty members in the area of Social and Legal Sciences in Spain
Source: PLoS One. 2021 Jul 2;16(7):e0254250. doi: 10.1371/journal.pone.0254250 (PMC8253417; doi:10.1371/journal.pone.0254250)
Supplement: S1 File — (ZIP) [file pone.0254250.s001.zip › 2.2. BARRERAS.rtf]

Documento:		4. Ciencias Sociales y Jurídicas\P1 CCSS Creencias
Peso:	0
Posición:	66 - 69
Código:	2. Conocimientos\Conocimiento general discapacidad\2.2. Barreras
E: Y qué barreras crees tú que se encuentran los estudiantes cuando estudian en la universidad.
P1: Muchos tipos. 
E: Tanto barreras como ayudas me gustaría que me dijeras.
P1: Pues las arquitectónicas, las barreras físicas, las sociales, las que se encuentran en su entorno con el profesor y el alumnado que no entiende que tienes unas circunstancias específicas que necesitan un apoyo, pues específico también. Y las ayudas que necesitan son que se escuche y que haya interés por adaptar el entorno a sus condiciones para que puedan competir en las mismas condiciones que el resto.


Documento:		4. Ciencias Sociales y Jurídicas\P2 CSS Diseños
Peso:	0
Posición:	13 - 13
Código:	2. Conocimientos\Conocimiento general discapacidad\2.2. Barreras
P2: Todo lo que es el entorno, el ambiente tiene que ser idóneo. Va a depender del alumno específico, vuelvo a repetir, del tipo de necesidades específicas que tenga. El diseño de un aula es fundamental, eso está claro. Yo, por ejemplo, me he dado cuenta que, para las personas con silla de ruedas, no tienen sitio, ¿cómo se sientan? No hay bancas adaptadas para ese tipo de personas. O personas que tengan que escribir en Braille, que también las he tenido.


Documento:		4. Ciencias Sociales y Jurídicas\P3 CSS Creencias
Peso:	0
Posición:	64 - 65
Código:	2. Conocimientos\Conocimiento general discapacidad\2.2. Barreras
E: Muy bien. Vamos a pasar ahora P3 al bloque de conocimientos. Y desde el punto de vista general, ¿cuáles crees tú que son las principales barreas y ayudas que se encuentran los estudiantes con discapacidad cuando estudian en la universidad?
P3: Yo no creo que sean tanto las barreras físicas, porque eso se está siempre intentado ir corrigiendo, sino que creo que son las barreras de atención, en la forma de atender al alumno con discapacidad, porque como no hay ningún procedimiento, pues se deja en la mano del profesor lo que cada uno vaya hacer. Entonces, creo que así dificultamos más las cosas a los alumnos con discapacidad. Entonces, si hubiera algún procedimiento, pues le ayudaríamos mucho más. Pero, esto no existe a día de hoy.


Documento:		4. Ciencias Sociales y Jurídicas\P4 CCSS Creencias
Peso:	0
Posición:	68 - 69
Código:	2. Conocimientos\Conocimiento general discapacidad\2.2. Barreras
E: Y qué barreras crees que los estudiantes encuentran cuando estudian en la universidad.
P4: Muchas. Por ejemplo, un estudiante con discapacidad física no puede ir a un aula y sentarse, por lo menos la experiencia que yo tengo…yo sé que depende del centro, del aula… Pero me acuerdo que en el caso de los estudiantes con discapacidad física, pues estaba en una mesa aparte, en la parte izquierda, en un rincón en la esquina puesta y eso no es estar integrado. Le ponían la mesita, algunas veces llegaba y no estaba la mesa, había que buscarla por todas las aulas… Para este estudiante no era agradable. 


Documento:		4. Ciencias Sociales y Jurídicas\P4 CCSS Creencias
Peso:	0
Posición:	71 - 71
Código:	2. Conocimientos\Conocimiento general discapacidad\2.2. Barreras
 Pero claro, aquí en mi departamento, que hay dos partes, yo que estoy en la parte de abajo como tú has visto, cuando viene un estudiante con discapacidad física no puedo atenderlo en mi despacho. Entonces, ya empiezan los problemas, ahora yo subo, tenemos que buscar un sitio…


Documento:		4. Ciencias Sociales y Jurídicas\P4 CSS Diseños
Peso:	0
Posición:	39 - 39
Código:	2. Conocimientos\Conocimiento general discapacidad\2.2. Barreras
 Lo que no me parece justo es que una persona por tener discapacidad tenga que estudiar el doble o dedicar el doble de tiempo porque tenga muchas barreras, pero hay que estudiar, hay que esforzarse para aprender, si no, no funciona.


Documento:		4. Ciencias Sociales y Jurídicas\P5 CSS Creencias
Peso:	0
Posición:	17 - 17
Código:	2. Conocimientos\Conocimiento general discapacidad\2.2. Barreras
P5: Yo me refiero a la discapacidad pensando en alumno que tienes enfrente, que tú sabes que hay que tener un especial cuidado, una especial atención, quiero decir, es que este en concreto es que era de un nivel avanzado, pero en primero o segundo, todavía hay gente que se ríe de los compañeros, pero claro, yo soy mucho de preguntar en clase, y si empieza a hablar tartamudeando, pues no siempre se mantiene el respeto debido. Yo, por eso, quizá sea de las cosas en que yo soy muy severo, con el trato, tenga o no tenga discapacidad, pero, ya te digo, pensando estos días, ¿discapacidad? Este compañero tenía una discapacidad…no sabía si…


Documento:		4. Ciencias Sociales y Jurídicas\P5 CSS Creencias
Peso:	0
Posición:	80 - 81
Código:	2. Conocimientos\Conocimiento general discapacidad\2.2. Barreras
E: Sí, bueno, hay incluso un debate abierto sobre la nomenclatura que se debe utilizar para hablar sobre discapacidad. De hecho, la propia palabra “discapacidad” en sí, ya empieza a tener connotaciones…parece que estamos poniendo ya en la propia palabra una barrera, ¿no? Y, hablando de barreras, qué barreras crees que los estudiantes con discapacidad se encuentran cuando estudian en la universidad.
P5: La de los propios compañeros, y luego la de unos docentes que no están preparados para atender a esos alumnos vamos.


Documento:		4. Ciencias Sociales y Jurídicas\P6 CCSS Creencias
Peso:	0
Posición:	60 - 61
Código:	2. Conocimientos\Conocimiento general discapacidad\2.2. Barreras
E: ¿Y qué barreras crees que los estudiantes se encuentran cuando estudian en la universidad?
P6: Pues, por ejemplo, en primer lugar, creo que se encuentran muchas barreras de accesibilidad a las aulas, de accesibilidad a la cafetería, de accesibilidad… O sea, muchas barreras físicas creo que se encuentran, infinitas. Creo que no somos conscientes los que no las tenemos del número infame de barreras que tienen, en el caso de que sean físicas. Y luego, a la hora de hablar de otro tipo de discapacidades, primero creo que se encuentran con profesores que no están formados para atenderlos, que a lo mejor ni están formados ni quieren dedicar tiempo a atenderlos porque a lo mejor nadie les está diciendo que lo hagan. Y, entonces, creo que en un primer contexto se pueden encontrar con que esto es bueno para mucha parte de las discapacidades, pero que claro, dada la inconsciencia que tenemos los profesores de que tenemos alumnado con discapacidad, los tratamos por igual, que está muy bien también, por otro lado, está muy bien, pero creo que la universidad debería de un poco, hacer equilibrio entre esas dos partes.


Documento:		4. Ciencias Sociales y Jurídicas\P7 CCSS Creencias
Peso:	0
Posición:	74 - 75
Código:	2. Conocimientos\Conocimiento general discapacidad\2.2. Barreras
E: ¿Y las personas con discapacidad tienen una especial barrera?
P7: Y por supuesto, personas que encima tienen, encima de los anteriores problemas, tienen la especial dificultad que hay que, en esa edad, tener una discapacidad más luego lo añadido que hemos dicho, que ya una persona en la adolescencia viene con muchos problemas de que está en procesos de transición, de cambios, si encima tiene una discapacidad pues mayor…


Documento:		4. Ciencias Sociales y Jurídicas\P8 CCSS Creencias
Peso:	0
Posición:	154 - 169
Código:	2. Conocimientos\Conocimiento general discapacidad\2.2. Barreras
E: ¿Y esas son las barreras que tú crees que el alumno encuentra con mayor frecuencia, las de accesibilidad, las de acceso?
P8: No, esa es una clara porque es física de llegar al sitio. Ahora, una vez que ya esté dentro, me puedo encontrar con insensibilidad por parte del profesorado, la que quieras y un poco más. Porque yo me imagino alguno que dirá “¡qué horror! Pues ni le miro”.
E: O que le dice al alumno, “ah, pues yo evalúo así” o “mis clases son así”, “pero mire usted, es que yo no veo la pizarra”.
P8: “Pues te aguantas”, “pues te buscas la vida, vamos”. Pues eso es lo que te digo, que no. Segundo, no tenemos un alumnado, sinceramente eh, ni solidario ni receptivo.
E: Entre ellos, ¿no?
P8: Son súper competitivos. Y entonces, la competitividad bien entendida es un refuerzo para la superación, pero yo no la veo bien entendida. Yo veo reacciones en alumnos que es que no doy crédito. O sea, a mí, cuando un alumno viene a quejarse, con todo su derecho, de que es que está un grupo y los otros no hacen nada, lo que le digo es “dentro de tus habilidades, tiene que estar el que tus compañeros reaccionen y hagan. No vengas a chivarte y, si no, este trabajo de grupo, no es como tal y vienes con ellos y ellos refuerzan esa idea”, “no, es verdad, no hemos currado nada”. Yo en mi época, sabíamos que siempre había alguno que era el que traía la pizza y con el que nos partíamos de risa.
E: Y esa es tu aportación al grupo, ¿no?
P8: Sí, no sé. Me da la sensación de que estamos creando a personas muy combativas, pero mirando sus propios intereses.
E: Es decir, tú crees que una de las barreras del alumnado con discapacidad son los propios compañeros.
P8: Yo creo que sí. O sea, yo, por ejemplo, vamos a ver, no sé si lo ocultaban, no puedo decir eso, pero no lo defendían, eso está claro. Pero yo en su situación hubiese hecho lo mismo. Yo no me atrevería a saber cómo responder 54 a una posición mía complicada. Te voy a contar otra cosa, no es de discapacidad, pero a ver, yo he tenido una niña con depresión de enfermedad. No es la tontería que decimos “no, está deprimida”.
E: Crónica, ¿no?
P8: Una depresión medicada, ¿no? No quería que se supiera en clase hasta el punto de que, en algún momento, alguien, que es una frase tonta “que estás loca”, dirigida a ella, era como…
E: Una ofensa impresionante.
P8: Una ofensa y no lo sabía articular. Te digo, o sea, porque a ver, a lo mejor es que yo soy muy madre…no es que sea buena docente, que no tiene nada que ver, soy bastante receptiva con los alumnos. No sé por qué se crea eso, yo creo que es porque les doy facilidades de comunicación, pero, para que te hagas una idea, el año pasado se suicidó la madre de un estudiante mío. La niña vino a contarme, “mi madre se ha suicidado, estoy fatal” y te digo, no era problema de este chico y ahí le dije “ningún problema”. Ahí estábamos el día 22, con la lotería puesta en internet por si nos tocaba el gordo que nos íbamos a ir a emborrachar juntas, estudiando el examen porque le iba a examinar una semana después, es decir, yo no puedo ser insensible a una situación de ese tipo. No puedo, o sea, y me pidió por favor que no comentara nada en clase. Solo se lo había dicho a una persona porque su madre era una persona depresiva y estaba en tratamiento y se había suicidado. Tenía una depresión que le llevó a suicidarse. Entonces, me decía “es que yo sé que algunos se lo toman a cachondeo y me dicen la hija de la loca, y tú que siempre estás triste vas a hacer lo mismo”, o sea… por eso te digo que me parece que la calidad humana y esa colaboración entre compañeros, ese compañerismo en el fondo, falta.
E: Falta…
P8: Falta muchísimo.


Documento:		4. Ciencias Sociales y Jurídicas\P11 CCSS Creencias
Peso:	0
Posición:	69 - 70
Código:	2. Conocimientos\Conocimiento general discapacidad\2.2. Barreras
E: ¿Qué barreras crees que los estudiantes con discapacidad encuentran cuando estudian en la universidad?
P11: Yo creo que hay una primera barrera muy típica que también los profesores nos encontramos que es la rigidez que supone que al ser una universidad, tiene que haber unos instrumentos estructurales porque se mueven muchas personas y hay un programa del que no te puedes mover, unas aulas asignadas y ahí no te puedes mover. Entonces, hay unas pautas y tú te tienes que adaptar. Y si a ti como persona no discapacitada ya te cuesta seguir el ritmo de que esto es así y es así, pues una persona que pueda tener una discapacidad, pues lógicamente eso ya es una barrera de que tiene que seguir unas pautas y que tiene que asumir rápidamente y quizá no es tan fácil. Esa sería la primera, la rigidez de los programas y estructura general de la universidad. Es posible que pueda haber barreras físicas, esto se cuida mucho, pero no nos olvidemos de que son aularios, y que una persona con una deficiencia visual, pues yo no sé hasta qué punto es más o menos cómodo, lo desconozco. Para una persona que por ejemplo tenga asperger, a mí lo que me pedían es si podía hacerle el examen a solas porque se estresaba. Bueno, si se estresa en un examen también puede estresarse en un aula. Yo no sé hasta qué punto, las aulas, de sonido, de luz, y demás están confeccionadas para personas hipersensibles. Lo desconozco. Luego, sí que puede haber unas barreras físicas. Y luego, hay unas barreras mentales de profesores o compañeros o personal de la universidad, qué de formación o trato o experiencia tienen para personas con discapacidad. Claro, yo la primera vez que me enfrenté a una persona con asperger fue con este alumno. Cuál puede ser mi reacción si de repente el alumno me dice “eres un hijo de puta”, yo qué tengo que hacer si me lo hace delante de clase. No tengo queja, nunca me insultó, todo lo contrario, pero a los compañeros sí sé que les dijo palabras fuertes. Qué haces.


Documento:		4. Ciencias Sociales y Jurídicas\P11 CCSS Creencias
Peso:	0
Posición:	74 - 74
Código:	2. Conocimientos\Conocimiento general discapacidad\2.2. Barreras
Entonces, eso es un problema de falta de medios y de interés político porque es muy bonito decir que tendremos tantos profesores y tanto presupuesto, pero luego la realidad supera la atención a este tipo de personas, luego, sí entiendo que hay una falta de atención brutal a este tema.


Documento:		4. Ciencias Sociales y Jurídicas\P12 CCSS Creencias
Peso:	0
Posición:	94 - 95
Código:	2. Conocimientos\Conocimiento general discapacidad\2.2. Barreras
E: ¿Qué barreras crees que los estudiantes con discapacidad se encuentran cuando estudian en la universidad?
P12: En la universidad las barreas arquitectónicas creo yo que están completamente eliminadas. Entonces, aquellos que tienen problemas de movilidad, pues moverse se pueden mover muy bien. Otra cosa, son aquellos que te dicen, “es que necesito ir al aseo y yo necesito a una persona”, pues esto es una barrera porque a lo mejor la facultad no tiene prevista esta persona para que le ayude. O a lo mejor para comer, esta persona para comer también necesita una ayuda porque no puede comer sola. Y  esto no es una barrera arquitectónica, pero es otro tipo de barrera que la facultad tiene que tener prevista. Ya te digo que todo está fuera del aula, así que desconozco si la universidad tiene prevista este tipo de ayuda. En el tema de la discapacidad visual, pues imagino que si no ves nada, pues necesitarán a alguien que les coloque porque una vez que están ubicado se orientan bastante bien, pero claro al principio tienen que traer una persona. Y después, como son como cualquier otro estudiante, pues los estudiantes muchas veces tienen que ir a presentar instancia, pues imagino que eso supondrá un problema. Todo tema burocrático administrativo tiene que ser un problema.


Documento:		4. Ciencias Sociales y Jurídicas\P13 CCSS Creencias
Peso:	0
Posición:	24 - 24
Código:	2. Conocimientos\Conocimiento general discapacidad\2.2. Barreras
Sé que hay gente que no está dispuesta a dedicar ese margen adicional porque es tiempo que tienes que dedicar y no está previsto en tu POD, pero bueno, y es lo que yo te decía, son estudiantes con ganas de aprender, y si lo demuestran, el éxito sirve para compensar estos costes de tiempo. Y en mi caso particular, los estudiantes que he tratado han sido muy comprometidos y eso supera todo lo que tienes que poner tú, que tampoco es tanto. Ya te digo que las nuevas tecnologías son un avance total en estos casos especiales y ellos también las manejan muy bien.


Documento:		4. Ciencias Sociales y Jurídicas\P15 CCSS Creencias
Peso:	0
Posición:	84 - 85
Código:	2. Conocimientos\Conocimiento general discapacidad\2.2. Barreras
E: Vale. Y qué barreras cree que los estudiantes con discapacidad pueden encontrarse cuando estudian en la universidad.
P15: Pues depende de la discapacidad que tengan. Ya te digo, los hay que, por lo que sea, padecen de nerviosismo. Entonces, quizá necesiten más tiempo para el examen o relajarse o estar solos y no con otros alumnos o… No sé. Y hay otros que tienen una discapacidad física que lo que necesitan es eso, un examen con una letra mayor, también más tiempo… Hay otros que necesitan a alguien que les escriba porque ellos no pueden escribir… Depende de cada uno.


Documento:		4. Ciencias Sociales y Jurídicas\P19 CCSS Creencias
Peso:	0
Posición:	62 - 63
Código:	2. Conocimientos\Conocimiento general discapacidad\2.2. Barreras
E: ¿Qué barreras crees que los estudiantes con discapacidad se encuentran cuando estudian en universidad?
P19: Bueno, barreras se pueden encontrar alguna arquitectónica, porque el edificio es muy muy antiguo, era un antiguo hospital de peregrinos de la Edad Media hoy día rehabilitado, es muy bonito pero antiguo, y puede haber algo que se escape, aunque creo que ahora mismo hay ascensores hacia todas las plantas, las escaleras han sido sustituidas por rampas, no sé, creo que no hay barreras.


Documento:		4. Ciencias Sociales y Jurídicas\P20 CCSS Creencias
Peso:	0
Posición:	72 - 73
Código:	2. Conocimientos\Conocimiento general discapacidad\2.2. Barreras
E: Muy bien, y antes me has hablado de que la universidad es inclusiva, pero, ¿crees que exista alguna barrera todavía que el alumnado pueda encontrarse en la universidad?
P20: Mira, yo creo que puede haber una barrera, que parecerá una tontería, pero es una barrera arquitectónica, en muchos sitios. Personas en silla de ruedas y demás, que cuesta, cuesta. O, por ejemplo, ordenadores más específicos con pantalla más grande para personas con discapacidad visual… Todo este tipo de cuestiones. O sea, estoy hablándote de barreras físicas más que de otro tipo.


Documento:		4. Ciencias Sociales y Jurídicas\P21 CCSS Creencias
Peso:	0
Posición:	146 - 147
Código:	2. Conocimientos\Conocimiento general discapacidad\2.2. Barreras
E: Y, ¿qué barreras cree que los estudiantes con discapacidad encuentran cuando estudian en la universidad?
P21: Tengo la impresión, porque insisto, no es una cosa que yo tenga estudiada como certeza. Tengo la impresión de que se siguen encontrando barreras físicas, ¿vale? Aunque es verdad que en eso hemos evolucionado, pero quizás la principal barrera, aunque pueda parecer que eso, incluso en determinados sentidos pueda ser una ventaja, pero yo siempre considero un inconveniente, lo que estábamos diciendo, el que se les considere desde la superioridad de los “pobrecitos discapacitados”. Yo creo que esa barrera no les hace ningún favor. Y hay todavía una barrera mental, que es una barrera social y la universidad es un reflejo de la sociedad. No somos nada especial. Y yo sí he visto el “pobrecito”. Y para mí eso es una barrera.


Documento:		4. Ciencias Sociales y Jurídicas\P22 CCSS Creencias
Peso:	0
Posición:	90 - 93
Código:	2. Conocimientos\Conocimiento general discapacidad\2.2. Barreras
E: Y, ¿qué barreras crees que los estudiantes con discapacidad se encuentran cuando estudian en la universidad?
P22: ¿Barreras?
E: Sí, obstáculos. 
P22: Yo creo que los normales de su discapacidad, ¿no? Es decir que, si son ciegos, pues tendrán que tener un ordenador especial, ¿no? Pero eso no lo facilita la universidad yo creo, que lo tienen ellos. También facilitar eso. Ahora, ya te digo, yo creo que es importante saber porcentajes, cantidades… Porque hablamos también de un dinero público y si vamos a destinarlo habrá que solicitarlo, habrá que pedirlo, en función de…si tenemos un 20% del alumnado, qué condiciones, o qué podemos tener para eso. Si tenemos un 1% seguramente no nos lo darán. Entonces, eso es importante, ¿no?


Documento:		4. Ciencias Sociales y Jurídicas\P23 CCSS Creencias
Peso:	0
Posición:	78 - 81
Código:	2. Conocimientos\Conocimiento general discapacidad\2.2. Barreras
E: Y, ¿qué barrera cree que los estudiantes con discapacidad se encuentran cuando estudian en la universidad?
P23: Bueno, se encuentran varias barreras. La principal barrera son los compañeros, el saber si van a ser aceptados o no, el sentirse diferentes o no... Luego, las barreras físicas: que tienen que adaptarse a los espacios y demás. ¿Qué más? El hecho de tener todos los materiales adaptados a todas las capacidades plenas, que yo me imagino que se encontrarán con profesorado de todo tipo. Algunos que están dispuestos, otros que no. Por ejemplo, yo no he escuchado decir a profesores que no deberíamos facilitarles porque luego no van a poder ejercer. O que si alguien con discapacidad termina una carrera está mal planteado el plan de estudios, o cosas así. Como menospreciando, como que le bajamos el listón para que lo consigan, ¿no? Entonces se encontrarán con ese tipo de actitudes también.
E: Que hay de todo.
P23: Claro.


Documento:		4. Ciencias Sociales y Jurídicas\P24 CCSS Creencias
Peso:	0
Posición:	29 - 29
Código:	2. Conocimientos\Conocimiento general discapacidad\2.2. Barreras
 Qué sucede, como dice Bourdier, tienen tan interiorizados en ellos mismos lo que la universidad les ha hecho ver, que no sirven para nada, que ni ellos mismos se creen con el potencial de que tienen esos conocimientos. 


Documento:		4. Ciencias Sociales y Jurídicas\P24 CCSS Creencias
Peso:	0
Posición:	40 - 41
Código:	2. Conocimientos\Conocimiento general discapacidad\2.2. Barreras
E: Claro. Y acerca de las barreras y ayudas que los estudiantes con discapacidad encuentran en la universidad, cuáles crees que son las que encuentran.
P24: A ver, cómo te diría…te he dicho al principio que esta es una universidad muy grande, histórica, hay patrimonio de la humanidad, que es intocable, por el tema de que fue un frente de guerra y está protegido, y no son tan accesibles. Entonces, se buscan los medios, pero hay veces que no. Después, la estructura de algunos edificios, y hablo del de la facultad de económicas, de la época de la dictadura, y es un edificio con muchas dificultades porque en ese sentido, el diseño era para que entraran los caballos y acorralar a los estudiantes en el otro extremo. Entonces, hay muchísimos escalones. Todo eso la universidad está trabajando en ello y ahora mismo estamos trabajando para detectar los sitios más accesibles de la universidad. Yo creo que las mayores barreras son las que tenemos cada uno de nosotros, las interiores, las que no se ven. Esa es la más difícil de conseguir. Hay muchos autores que hablan de eso. Quién es la discapacidad, qué es, dónde está escrito que tenemos que tener tanto de tanto, dónde. Es una construcción social, vamos lo veo y lo seguiré manifestando. No le quito el valor a los argumentos científicos, porque ellos tienen que mirarlo desde la parte sanitaria desde otra óptica, pero desde lo social, es una construcción que hemos diseñado.


Documento:		4. Ciencias Sociales y Jurídicas\P25 CCSS Creencias
Peso:	0
Posición:	96 - 97
Código:	2. Conocimientos\Conocimiento general discapacidad\2.2. Barreras
E: ¿Qué barreras crees que encuentran los estudiantes cuando están estudiando en la universidad?
P25: Yo creo que los estudiantes se encuentran con la barrera de lo que es el contenido de la materia. Que el profesor se plantea como hemos dicho antes, el trabajo de adaptación en muchos casos o el chaval se va a encontrar con serias dificultades, ¿no? Pero también, depende de la discapacidad que tenga, si tú tienes una discapacidad física, pero intelectualmente estás bien, por qué vas a tener algún problema, ¿no? Ahora, si eres sordo, si no ves, si tienes un nivel…los autistas son súper inteligentes, están y no están, pero cuando están sacan jugo de dónde no sacan los otros, ven lo que los otros no ven. Entonces, las barreras, pues dependerán del problema que tenga el chico o la chica, ¿no?


Documento:		4. Ciencias Sociales y Jurídicas\P25 CCSS Diseños
Peso:	0
Posición:	41 - 41
Código:	2. Conocimientos\Conocimiento general discapacidad\2.2. Barreras
Y luego, está el aula, el aspecto físico es muy importante. Y en este sentido, alumnos con discapacidad lo tendrían difícil. 
