# Supplementary material for: Is pedagogical training an essential requirement for inclusive education? The case of faculty members in the area of Social and Legal Sciences in Spain
Source: PLoS One. 2021 Jul 2;16(7):e0254250. doi: 10.1371/journal.pone.0254250 (PMC8253417; doi:10.1371/journal.pone.0254250)
Supplement: S1 File — (ZIP) [file pone.0254250.s001.zip › 2.3. AYUDAS.rtf]

Documento:		4. Ciencias Sociales y Jurídicas\P2 CCSS Creencias
Peso:	0
Posición:	74 - 75
Código:	2. Conocimientos\Conocimiento general discapacidad\2.3. Ayudas
E: Vale. Ya me has hablado de las barreras que crees que encuentran, pero ¿qué ayudas crees que tienen?
P2: Que yo sepa, muy pocas, vamos, que yo sepa, tampoco nunca nadie nos ha hablado sobre eso. Podríamos decir que puede haber discrepancias en función de cada campus en el que estemos. En este campus, al ser moderno, pues no tendría problemas una persona minusválida para nada, porque está completamente adaptado para los minusválidos. Ahora, vete a un edificio antiguo, que ya las historias cambian. De todas formas, en España estamos muy adaptados en este sentido. Te vas a otra universidad extranjera, vete a la Sorbona, ahí no tienen nada vamos. Allí hay unas barreras arquitectónicas tremendas vamos, pero aún queda mucho por hacer.


Documento:		4. Ciencias Sociales y Jurídicas\P3 CSS Creencias
Peso:	0
Posición:	65 - 65
Código:	2. Conocimientos\Conocimiento general discapacidad\2.3. Ayudas
Las ayudas, pues el servicio de atención al alumno con discapacidad de la Universidad de Sevilla, cuando establece sus procedimientos, a veces quizás por la falta de recursos, es que hay muy pocas personas que puedan dar ese servicio, pero siempre intentamos ayudar.


Documento:		4. Ciencias Sociales y Jurídicas\P4 CCSS Creencias
Peso:	0
Posición:	70 - 71
Código:	2. Conocimientos\Conocimiento general discapacidad\2.3. Ayudas
E: Y qué ayudas.
P4: Bueno, yo creo que sí existen. Volviendo al tema de discapacidad física, creo que está todo mucho más adaptado, dentro de…aquí vas por el campus y hay muchas rampas, ¿no? Pero claro, aquí en mi departamento, que hay dos partes, yo que estoy en la parte de abajo como tú has visto, cuando viene un estudiante con discapacidad física no puedo atenderlo en mi despacho. Entonces, ya empiezan los problemas, ahora yo subo, tenemos que buscar un sitio… Entonces, ya no…con los estudiantes con discapacidad intelectual, no es el caso que yo he tenido, pero por reflexionar un poco sobre otras discapacidades. Discapacidad de tipo mental, pues que, además, discapacidad física, visual, auditiva… Quizá sean más manejables, pero discapacidad mental…ahí se necesita mucho apoyo, prestar mucha ayuda, darle opciones, trabajar mucho la motivación, sentirse que está apoyado, facilitar la evaluación y no “venga un examen de tres horas”, pues a lo mejor eso no puede ser. En cualquier caso, nosotros como profesores, en la evaluación, a la hora de determinar si el estudiante ha adquirido los conocimientos suficientes para superar y pasar la asignatura, pues plantearse de que otra forma se puede plantear en ese caso… Es que son muchas cosas las que se pueden plantear.


Documento:		4. Ciencias Sociales y Jurídicas\P4 CCSS Creencias
Peso:	0
Posición:	74 - 75
Código:	2. Conocimientos\Conocimiento general discapacidad\2.3. Ayudas
E: Claro. Y ahora más específicamente sobre la discapacidad, ¿sabes si existe normativa universitaria sobre discapacidad?
P4: Existe una normativa sobre discapacidad, que se le dan a los estudiantes una serie de derechos de tipo, bueno, pues lo que te comentaba antes de las adaptaciones curriculares. Tienen derecho a que se le realice una adaptación curricular de forma que la metodología, la evaluación, todo esté adaptado a su discapacidad y tienen derecho en el caso de acceder a la matrícula ayudas, estudiantes colaboradores… Sé que hay muchas ayudas a las que pueden acceder.


Documento:		4. Ciencias Sociales y Jurídicas\P4 CCSS Creencias
Peso:	0
Posición:	80 - 81
Código:	2. Conocimientos\Conocimiento general discapacidad\2.3. Ayudas
E: Y como docente, qué estrategias de enseñanza conoces que favorezcan y motiven más el aprendizaje de los estudiantes.
P4:  Yo creo que lo primero que les motiva es que los tengas en consideración, lo primero que creo que les hace no rendirse, no abandonar, es que los tengas en consideración y te preocupes por ellos para ayudarle en esas trabas y barreras que ellos se encuentran, que sepan que van a tener un apoyo. Ahí, eso creo que es lo que más les ayuda.


Documento:		4. Ciencias Sociales y Jurídicas\P5 CSS Creencias
Peso:	0
Posición:	82 - 83
Código:	2. Conocimientos\Conocimiento general discapacidad\2.3. Ayudas
E: Y qué ayudas crees que se encuentran.
P5: Pues las de ciertos compañeros y ciertos profesores que se comprometan, ¿no? Con el instrumento que tengan. Quiero decir, que muchas veces puede que hagamos más por la integración el buena gente de la clase que se acerca al alumno con discapacidad y lo integra, ¿no? No sé. Vete tú a saber las herramientas metodológicas que puede poner uno en marcha.


Documento:		4. Ciencias Sociales y Jurídicas\P6 CCSS Creencias
Peso:	0
Posición:	48 - 49
Código:	2. Conocimientos\Conocimiento general discapacidad\2.3. Ayudas
E: Y, ¿conoces alguna acción concreta que haga, en este caso, tu universidad, la Pablo de Olavide, para favorecer la educación inclusiva?
P6: Sé que, por ejemplo, hay un centro de voluntariado universitario aquí y sé, por ejemplo, porque leí una noticia del boletín de aquí de la universidad, que entre los propios alumnos hay un voluntariado, por ejemplo, hay algunos alumnos que son ciegos y tienen siempre un compañero que los recoge, que va por ellos al metro o que queda con ellos y vienen a clase juntos y se van juntos… Entonces, sé que medidas hay, se me ha venido eso a la mente porque lo leí en una noticia de prensa y me ha parecido muy bonita y muy adecuada, pero no sabría decirte ninguna otra.


Documento:		4. Ciencias Sociales y Jurídicas\P8 CSS Diseños
Peso:	0
Posición:	30 - 31
Código:	2. Conocimientos\Conocimiento general discapacidad\2.3. Ayudas
E: Y qué ayudas crees que estos estudiantes encuentran cuando estudian en la universidad.
P8: Yo creo que, bueno, no sé si se les facilita, pero por lo menos no se les limita sus opciones de matrícula o se les apoya en los procesos de gestión de alumnos, eso lo tengo claro, porque la gente de gestión es estupenda, pero no conozco nada más, la verdad.


Documento:		4. Ciencias Sociales y Jurídicas\P12 CCSS Creencias
Peso:	0
Posición:	96 - 97
Código:	2. Conocimientos\Conocimiento general discapacidad\2.3. Ayudas
E: Y qué ayudas crees que encuentran en la universidad.
P12: Yo lo que he visto es que encuentran mucha colaboración por parte de los compañeros. Yo a estos estudiantes los veo como un estudiante más y siempre van acompañados. Yo me imagino que dentro de su grupo es donde tienen la ayuda más informal, a  parte de la formal que tiene la universidad. 


Documento:		4. Ciencias Sociales y Jurídicas\P13 CCSS Creencias
Peso:	0
Posición:	17 - 17
Código:	2. Conocimientos\Conocimiento general discapacidad\2.3. Ayudas
P13: Sí, normalmente sí. Y luego también es que todas las aulas aquí están habilitadas, hay espacios ya pensados. Así, por ejemplo, el que utiliza una silla de ruedas, pues hay una parte del aula, en las primeras filas, donde el espacio está liberado ara que puedan tener acceso. Así, con estos estudiantes nunca he tenido problemas de accesibilidad en el aula debido a barreras físicas.


Documento:		4. Ciencias Sociales y Jurídicas\P13 CCSS Creencias
Peso:	0
Posición:	22 - 22
Código:	2. Conocimientos\Conocimiento general discapacidad\2.3. Ayudas
Desde aquí no se le desanimó en ningún momento, nunca se le puso cortapisas, y eso hay que agradecerlo a la UJI porque siempre ha estado desde el primer momento para atender las diversidades, para responder a estas necesidades específicas. Y no solo desde la Unidad de Diversidad y Discapacidad, donde te aportan la información básica, sino también en otro tipo de órganos de la UJI como la ORI. Es la UDD la que te apoya en primer término. Allí se ponen en contacto con los estudiantes en el momento en que entran a la universidad, identifican sus necesidades educativas especiales, se las comunican a cada uno de los profesores implicados a través de una ficha confidencial interna, de forma que cada profesor sabe cuál es la diversidad, las necesidades específicas para tu asignatura y algunas sugerencias genéricas para adaptar la asignatura. Tú, como profesor, simplemente tienes que seguirla e ir afrontando luego el día a día según el contacto que tengas con el estudiante y la información que te vaya transmitiendo.


Documento:		4. Ciencias Sociales y Jurídicas\P15 CCSS Creencias
Peso:	0
Posición:	86 - 89
Código:	2. Conocimientos\Conocimiento general discapacidad\2.3. Ayudas
E: Y, ¿crees que encuentran algún tipo de ayuda cuando estudian en la universidad estos estudiantes?
P15: Aquí sí.
E: Qué tipo de ayuda crees que encuentran.
P15: Ya te digo, la adaptación de las pruebas y estas cosas, de la docencia, a las necesidades que tienen y también para la realización de los exámenes.


Documento:		4. Ciencias Sociales y Jurídicas\P19 CCSS Creencias
Peso:	0
Posición:	64 - 65
Código:	2. Conocimientos\Conocimiento general discapacidad\2.3. Ayudas
E: ¿Y qué ayudas? 
P19: Pues ayudas, muchas, está el alumno mentor, el servicio de atención a la discapacidad, los intérpretes, material adaptado, hay ayudas. 


Documento:		4. Ciencias Sociales y Jurídicas\P20 CCSS Creencias
Peso:	0
Posición:	74 - 75
Código:	2. Conocimientos\Conocimiento general discapacidad\2.3. Ayudas
E: ¿Y ayudas? Me has comentado el servicio, ¿conoces alguna ayuda más que tengan?
P20: Pues no te lo sé decir, no sé.


Documento:		4. Ciencias Sociales y Jurídicas\P22 CCSS Diseños
Peso:	0
Posición:	35 - 35
Código:	2. Conocimientos\Conocimiento general discapacidad\2.3. Ayudas
P22: Sí, las aulas aquí están bien preparadas, y, además, cuando tenemos un alumno, por ejemplo, en silla de ruedas, que es de los casos más comunes. Los casos más comunes, a te digo, son silla de ruedas y luego los problemas de visión, o ciegos completamente o con un porcentaje alto de poca visión, ¿no? Y entonces, yo creo que todo, la luz tiene muchísima todas las aulas, la acústica es buena, yo creo que es lo que te decía, los alumnos en silla de ruedas, hay aulas que tienen un espacio sin asientos para ellos, entonces, bueno, pues si tienes un alumno coges esa aula, si no tienes…pero yo creo que las condiciones son buenas.


Documento:		4. Ciencias Sociales y Jurídicas\P24 CCSS Creencias
Peso:	0
Posición:	17 - 17
Código:	2. Conocimientos\Conocimiento general discapacidad\2.3. Ayudas
Después, hay unas jornadas de técnicas de comunicación con personas con diversidad, que vienen del sector asociativo y de empresas para hablarnos profesionales sobre temáticas concretas: de la asociación de síndrome de Asperger, de la ONCE… Y después, la última mesa es de los propios estudiantes, de su experiencia en la universidad, con diversidad y del profesorado también. Después, el PAS se ha conseguido que se regularice y se tiene en cuenta en su formación continuada y cada vez va más PAS. El profesorado todavía no está regularizado…a ver, tenemos que ser conscientes, y si no es por voluntad propia, hay varios perfiles: personas, y digo personas para incluir a toda la comunidad universitaria, o les sale de dentro de sus genes, la empatía, la normalidad… Después, hay otro grupo que son ellos, ¿no? Y después hay otros que es porque tienen un familiar o alguien muy cercano con estas características. Entonces, sí o sí, la ley funciona…a ver, funciona relativamente, porque depende de la buena voluntad que tengan los técnicos, el equipo… Tenemos que decir que en esta universidad la implicación que tienen es 100% no, más. Y en la época del anterior rector, lo mismo, en el 2003 fue un despliegue muy grande el que se hizo, mucha implicación, se crearon las oficinas…


Documento:		4. Ciencias Sociales y Jurídicas\P24 CCSS Creencias
Peso:	0
Posición:	31 - 31
Código:	2. Conocimientos\Conocimiento general discapacidad\2.3. Ayudas
Perdona, la sociedad es muy variada, igual que el profesorado y, de hecho, también te tengo que decir que también tengo que trabajar con profesorado con diversidad o personas muy diversas. Y te voy a poner como ejemplo, de una conserje, que yo me quedé pasmada, porque resulta que entró en el servicio y yo le dije “oye, Cristina, y ese cuenco que hay en el suelo, qué es”, dice “para Conchita”, y Conchita es la perrita que tiene una de las estudiantes que es ciega. Y dice “este recipiente es solo para Conchita”, y que haya hecho eso, me ha encantado porque eso es humanidad. 


Documento:		4. Ciencias Sociales y Jurídicas\P25 CCSS Creencias
Peso:	0
Posición:	11 - 11
Código:	2. Conocimientos\Conocimiento general discapacidad\2.3. Ayudas
Una de las chicas ciegas, tenía su perrito guía, que venía a clases con su perrito guía, y la otra chica no necesitaba perro, pero sí necesitaba que las presentaciones fueran grandes, con letras grandes, en fin. Tenía una máquina en su mesa que se la proporcionó la universidad.


Documento:		4. Ciencias Sociales y Jurídicas\P25 CCSS Creencias
Peso:	0
Posición:	98 - 99
Código:	2. Conocimientos\Conocimiento general discapacidad\2.3. Ayudas
E: Y, ¿qué tipos de ayudas crees que encuentran en la universidad?
P25: Pues, sí hay ayuda. Esta chica que llegó y tenía dificultades, que necesita una lupa, pues aquí se gestionó rápidamente, aunque la lupa se la proporcionó la ONCE, pero aquí se gestionó rápidamente. El perro estaba en clase, aquí no hubo ningún problema, ninguna pega, es decir, aquí se facilitó todo el acceso de los chicos y de las chicas, por lo menos en mi experiencia. Ellos han tenido apoyo.
