# Supplementary material for: Is pedagogical training an essential requirement for inclusive education? The case of faculty members in the area of Social and Legal Sciences in Spain
Source: PLoS One. 2021 Jul 2;16(7):e0254250. doi: 10.1371/journal.pone.0254250 (PMC8253417; doi:10.1371/journal.pone.0254250)
Supplement: S1 File — (ZIP) [file pone.0254250.s001.zip › 2.4. EDUCACION INCLUSIVA.rtf]

Documento:		4. Ciencias Sociales y Jurídicas\P4 CCSS Creencias
Peso:	0
Posición:	72 - 73
Código:	2. Conocimientos\Conocimiento general discapacidad\2.4. Educación inclusiva
E: Y qué conoces sobre la educación inclusiva.
P4: Pues no sé si conozco mucho, sé que se trata de integrar, de trabajar con grupos de personas en las que todos se sienten integrados, y transmitir la educación desde que esa persona forme parte del conjunto, ¿no?


Documento:		4. Ciencias Sociales y Jurídicas\P5 CSS Creencias
Peso:	0
Posición:	84 - 85
Código:	2. Conocimientos\Conocimiento general discapacidad\2.4. Educación inclusiva
E: Y qué conoces sobre la educación inclusiva.
P5: Nada.


Documento:		4. Ciencias Sociales y Jurídicas\P6 CCSS Creencias
Peso:	0
Posición:	62 - 63
Código:	2. Conocimientos\Conocimiento general discapacidad\2.4. Educación inclusiva
E: Muy bien. Y, qué conoces acerca de la educación inclusiva.
P6: Pues conozco poco también, imagino que será, pues eso, un concepto que permite tomar una serie de medidas, utilizar una serie de herramientas para que la enseñanza sea un proceso en el que todo el mundo quepa, ¿no? Independientemente de las circunstancias de cada uno.


Documento:		4. Ciencias Sociales y Jurídicas\P7 CCSS Creencias
Peso:	0
Posición:	88 - 91
Código:	2. Conocimientos\Conocimiento general discapacidad\2.4. Educación inclusiva
E: Vale. ¿Qué conoces sobre la educación inclusiva?
P7: Pues yo es que estoy trabajando en temas de servicios y estrategias sociales, de consultoría, hice varios planes estratégicos, de igualdad de oportunidades, de hombre y mujer, para la Junta de Castilla y León, para el Cabildo de Tenerife. Y entonces, de ahí tengo alguna cosa. Luego formación también en cuanto a que la vida misma me ha hecho pasar por…yo pertenezco a una familia numerosa, pues eso también te hace ser más sensible a diferentes personalidades. Luego, mi hermano hizo la tesis, que mi hermano es profesor de música, e hizo la tesis sobre la inclusión de inmigrantes y cómo la música…
E: Como elemento integrador.
P7: Eso. Las diferentes culturas y cómo integrarlo en la música. Ahí también, yo me acuerdo que, hasta que defendió la tesis, estuvimos discutiendo cosas, viendo…le ayudé yo también un poco con metodología y ahí aprendí también sobre el tema de inclusión, que no veía. Y sobre todo la experiencia, el ver casos.


Documento:		4. Ciencias Sociales y Jurídicas\P8 CSS Diseños
Peso:	0
Posición:	32 - 33
Código:	2. Conocimientos\Conocimiento general discapacidad\2.4. Educación inclusiva
E: Y acerca de la educación inclusiva, qué conoces. No sé si conoces el término o si…
P8: Conozco el término, conozco el objetivo, y tengo un interrogante, porque siempre estamos en inclusión para gente con menores capacidades, pero creo que se ha abandonado la inclusión de gente con altas capacidades que lo que se les manda es a guetos de gente como genios locos, y eso sí que te lo digo por experiencia personal, es decir, la gente con altas capacidades tienen problemas porque cuando se les detecta, lo que te dicen es que les lleves a un centro internado donde van a estar rodeados de gente que está estudiando con 14 años astronomía, ¿sabes? Cuando lo que tienes que hacer es adaptarlo al contexto y que sepan…porque son gente con problemas de relaciones personales, más cerrados… Precisamente por eso, porque sus altas capacidades, le impiden comunicarse con gente de su misma edad, por ejemplo. Sus preferencias y gustos son muy extraños… Entonces no encajan y en lugar de intentar que encajen, lo que hacen es aglutinarlos en un centro de altas capacidades. Entonces, creo que cuando se habla de educación inclusiva, debería plantearse, para los que tienen altas capacidades y para aquellos que también las tienen, pero por sobre dotación. Entonces, esa es mi gran crítica al sistema de educación inclusiva. Y te diría, no sé si tanto a la universidad, pero sí a nivel inferior, mi experiencia dice que la incorporación de niños con dificultades en la escuela, te hablo de niños con síndrome de Down, por ejemplo, aportan unos valores o una capacidad de ayuda intrapersonal espectacular, no para la persona 	que tiene ese problema, sino para los compañeros, también te lo digo por experiencia. O sea, en un aula donde hay un niño Down, se crea una sensibilidad y una valoración y un sentimiento de ayuda y apoyo entre los niños que es extremadamente positiva. 


Documento:		4. Ciencias Sociales y Jurídicas\P10 CCSS Creencias
Peso:	0
Posición:	92 - 93
Código:	2. Conocimientos\Conocimiento general discapacidad\2.4. Educación inclusiva
E: Sí. Y, ¿sobre educación inclusiva?
P10: Hombre, yo creo que, la mayoría de estas cosas desde mi punto de vista, donde más efectivas pueden ser es cuanto más pequeños, mejor. Desde mi punto de vista. Que la sociedad vaya viendo con normalidad determinadas cosas. No sé. Entonces, yo lo veo así. Si eso fuese así, no tendríamos que preguntarnos cuando lleguemos a la universidad que... Sino que bueno, pues normal. Pues este es rubio, pues este tiene los ojos verdes, el otro... ¿Vale? Y eso no es limitativo para determinadas cosas, pues para otras sí. No sé. Es decir, “¿daltónico?” Pues evidentemente si usted quiere hacer bellas artes y bueno, a lo mejor ahí es complicado y tiene que... Pero que tampoco significa que lo veamos como un... No sé. Pues bueno, es así. Ya está. No sé. A lo mejor pues tienen una capacidad para ver volúmenes y ver formas y tal que los demás ni puñetera idea por más que sean muy finos con los colores. No lo sé.


Documento:		4. Ciencias Sociales y Jurídicas\P11 CCSS Creencias
Peso:	0
Posición:	73 - 74
Código:	2. Conocimientos\Conocimiento general discapacidad\2.4. Educación inclusiva
E: Y qué conoces de la educación inclusiva. Si conoces o te sugiere algo.
P11: Me sugiere una cosa y es muy fea. Por lo que yo sé, la ley de educación…bueno, ya sabes que todas las comunidades autónomas, cada una se monta la película a su manera, por lo que yo sé, que tengo muchos amigos que son maestros y que están en primaria o secundaria, que la ley dice una cosa y la práctica otra distinta. De la misma manera que dice que tiene que haber tantos alumnos como máximo por aula y que luego se cumple o incumple dependiendo de cada colegio, ya no te digo de cada ciudad, sino de cada colegio, también dicen que para una persona que necesita atención especial, necesita un profesor complementario, lo cual no se cumple siempre y por tanto genera frustración al alumno, profesor y compañeros. Y eso se repite en el colegio, instituto y luego aquí en la universidad también. Eso por un lado. Por otro, he visto la frustración de los padres, porque tengo familia que tienen un hijo con TDAH, es un alumno muy problemático, tiene muy difícil solución, y los profesores al final, cuando tienen un alumno que se inquieta le dicen “vete y no me generes problemas porque yo no puedo estar toda la clase atendiéndote a ti”. Entonces, eso es un problema de falta de medios y de interés político porque es muy bonito decir que tendremos tantos profesores y tanto presupuesto, pero luego la realidad supera la atención a este tipo de personas, luego, sí entiendo que hay una falta de atención brutal a este tema.


Documento:		4. Ciencias Sociales y Jurídicas\P12 CCSS Creencias
Peso:	0
Posición:	98 - 101
Código:	2. Conocimientos\Conocimiento general discapacidad\2.4. Educación inclusiva
E: ¿Qué conoces sobre la educación inclusiva?
P12: Nada.
E: ¿Tienes interés por conocer qué significa la educación inclusiva?
P12: Yo tengo interés en formarme en todo aquello que no conozco, pero el tiempo que tenemos es limitado. Entonces, lo que intento es focalizar el tiempo en todo aquello que me puede aportar algo. Entonces, si en algún momento determinado considero que esto me puede aportar algo, pues ahí que voy.


Documento:		4. Ciencias Sociales y Jurídicas\P14 CCSS Creencias
Peso:	0
Posición:	68 - 69
Código:	2. Conocimientos\Conocimiento general discapacidad\2.4. Educación inclusiva
E: Vale. Y, sobre educación inclusiva, ¿has oído alguna vez hablar sobre educación inclusiva?, ¿intuyes lo que es?
P14: Intuyo que es integrar personas con ciertas necesidades, pues especiales, en tareas o en proyectos… Pues eso, hacerlos partícipes y que se sientan parte, como el resto, sin discriminación.


Documento:		4. Ciencias Sociales y Jurídicas\P15 CCSS Creencias
Peso:	0
Posición:	90 - 93
Código:	2. Conocimientos\Conocimiento general discapacidad\2.4. Educación inclusiva
E: Vale. ¿Conoces el término de educación inclusiva?
P15: Sí.
E: Y qué conoces sobre ella.
P15: Pues eso, el tratar de, de alguna manera, tener en cuenta las necesidades que tienen, especiales, y tratar de ayudarles en ese sentido.


Documento:		4. Ciencias Sociales y Jurídicas\P17 CCSS Diseños
Peso:	0
Posición:	32 - 37
Código:	2. Conocimientos\Conocimiento general discapacidad\2.4. Educación inclusiva
E: ¿De educación inclusiva tampoco? ¿Discapacidad?
P17: Bueno, leyes no, leyes…
E: Lo que tú conoces a lo mejor por…
P17: Por mi trabajo, claro.
E: Por lo que me contaste de que habías sido gerente de…
P17: De una asociación de Prominusválidos Psíquicos.


Documento:		4. Ciencias Sociales y Jurídicas\P18 CCSS Creencias
Peso:	0
Posición:	82 - 83
Código:	2. Conocimientos\Conocimiento general discapacidad\2.4. Educación inclusiva
E: Sobre educación inclusiva, por ejemplo, ¿alguna vez te han mencionado el concepto?
P18: No, el concepto de educación inclusiva no. Más o menos yo lo entiendo y es el que pongo en práctica entendiendo como tal, pero no que alguien me haya formado sobre la educación inclusiva.


Documento:		4. Ciencias Sociales y Jurídicas\P19 CCSS Creencias
Peso:	0
Posición:	66 - 67
Código:	2. Conocimientos\Conocimiento general discapacidad\2.4. Educación inclusiva
E: ¿Qué conoces sobre la educación inclusiva?
P19: Pues nada, no conozco nada.


Documento:		4. Ciencias Sociales y Jurídicas\P20 CCSS Creencias
Peso:	0
Posición:	76 - 77
Código:	2. Conocimientos\Conocimiento general discapacidad\2.4. Educación inclusiva
E: Vale. Y qué conoces sobre la educación inclusiva, si lo has oído alguna vez, si lo conoces…
P20: Hombre, el término inclusivo sí que lo he oído muchas veces, pues un poco por lo que decíamos, porque siempre he estado muy pendiente de las cláusulas sociales y demás, entonces, todo lo que tiene que ver con determinados sectores o colectivos, pues sí que he estado más atenta, pero vamos, de educación inclusiva no conozco más de lo que de los términos pudieran derivarse.


Documento:		4. Ciencias Sociales y Jurídicas\P21 CCSS Creencias
Peso:	0
Posición:	148 - 153
Código:	2. Conocimientos\Conocimiento general discapacidad\2.4. Educación inclusiva
E: Totalmente. Y, ¿qué conoce sobre la educación inclusiva?
P21: Volvemos a lo mismo. Artículos que he leído, que...
E: ¿Qué diría que sería?
P21: En líneas generales, la educación inclusiva es el educar en la inclusión, en la aceptación de la diversidad, en fomentar la empatía. Que es lo fundamental en todas estas cuestiones, que es lo de ponerse siempre en el lugar del otro. Y que te ayude... Yo recuerdo siempre el caso de mi hija, que venía un día muy enfadada del cole porque...es verdad que ella siempre ha leído mucho. Y ella tenía conciencia de, en su ámbito...porque, además, los libros que mandaban se los leía, pero aparte de todo se leía...y en la clase, había una niña con discapacidad mental muy leve, que para los niños en parte era imperceptible, pero de la que los padres éramos conscientes. Y, entonces claro, ¿qué ocurre? Que cuando esa niña lee un libro en el colegio, en el cierre de curso dan las bandas, ¿no? A la mejor lectora, a la mejor no sé qué y no sé cuánto. Y bueno mi hija vino muy cabreada porque le habían dado la banda a la que más lee, a la mejor lectora de su clase a la... “papá, que se ha leído un libro”, ¿no? Y ella lo consideraba tremendamente injusto. Y ahí es donde tú tienes que ver la necesidad de educar en la inclusión. De hacerle ver y entender que ese libro...
E: Que ese libro es los diez que se ha leído ella.
P21: Efectivamente. También es cierto, que ahí ya hablé yo con la tutora, que hubiese facilitado mucho más las cosas dando un premio compartido en el que...porque eso fomenta el “pues no es justo”. Porque si todavía tú no lo entiendes...y, entonces, lo que sea dar argumentos...porque yo notaba en mi hija un cabreo de...un cabreo que vuelca sobre la compañera. Que vuelca sobre la compañera, ¿no? Y en ese sentido...


Documento:		4. Ciencias Sociales y Jurídicas\P22 CCSS Creencias
Peso:	0
Posición:	94 - 97
Código:	2. Conocimientos\Conocimiento general discapacidad\2.4. Educación inclusiva
E: Y, ¿qué conoce sobre la educación inclusiva?
P22: Sobre la…
E: Poco.
P22: La verdad es que no. 


Documento:		4. Ciencias Sociales y Jurídicas\P23 CCSS Creencias
Peso:	0
Posición:	82 - 83
Código:	2. Conocimientos\Conocimiento general discapacidad\2.4. Educación inclusiva
E: Y, ¿qué conoces sobre la educación inclusiva?
P23: Pues conozco a nivel personal simplemente. Yo no me he formado en esto, ni en la universidad como docente, ni en mi vida, sino que como tengo hijos, he escuchado las clases que nos dan a los padres. Y tengo una amiga que tiene un niño con autismo y la he acompañado a muchas de sus clases con su niño con autismo. Pero es a título personal todo.


Documento:		4. Ciencias Sociales y Jurídicas\P25 CCSS Creencias
Peso:	0
Posición:	100 - 101
Código:	2. Conocimientos\Conocimiento general discapacidad\2.4. Educación inclusiva
E: Y, ¿qué conoces, si conoces algo, sobre el término educación inclusiva?
P25: Es que para mí, la educación inclusiva es para tantas cosas, no solamente para la discapacidad. Es que yo pienso en minorías étnicas, yo he tenido alumnos gitanos, y para mí ha sido un éxito que  esos chicos estuvieran aquí y hayan logrado una carrera, para luego trabajar con su propio grupo, ¿no? Para mí, eso ha sido fabuloso. Educación inclusiva…eso es lo que tenía que ser la educación, pero desde la infantil, es que tenía que ser inclusiva. ¿Y qué significa inclusiva? pues, la apertura total para lo que te encuentres y lo que hay que hacer desde la infantil, es la integración., no separar a los chicos con problemas, no separar al grupo, no poner etiquetas, los listos por aquí…espérate, qué estás haciendo, ¿no?
