# Supplementary material for: Is pedagogical training an essential requirement for inclusive education? The case of faculty members in the area of Social and Legal Sciences in Spain
Source: PLoS One. 2021 Jul 2;16(7):e0254250. doi: 10.1371/journal.pone.0254250 (PMC8253417; doi:10.1371/journal.pone.0254250)
Supplement: S1 File — (ZIP) [file pone.0254250.s001.zip › Guion entrevista .docx]

GUIÓN ENTREVISTA. CREENCIAS Y CONOCIMIENTOS

Proyecto: Pedagogía inclusiva en la universidad: narrativas del profesorado (MINICO, ref. [EDU2016-76587-R](https://investigacion.us.es/sisius/sis_proyecto.php?idproy=27540))

**OBJETIVO:** Describir y explicar las creencias y conocimientos del profesorado universitario para desarrollar una pedagogía inclusiva en la universidad.

**Creencias:** concepciones, principios y máximas del profesorado universitario que le lleva a diseñar y desarrollar proyectos docentes para incluir a todo el alumnado.

**Conocimientos:** planteamientos teóricos, políticos y legislativos que conducen a los docentes a apostar por una pedagogía inclusiva.

Nombre y Apellidos: Años de experiencia docente:

Edad: Número de teléfono:

Correo electrónico: Departamento:

Área de conocimiento: Vinculación con la Universidad:

**INTRODUCCIÓN: EXPERIENCIA CON ESTUDIANTES CON DISCAPACIDAD (en caso de que haya tenido)**

- Breve descripción experiencia en discapacidad (tipos de discapacidad, nº alumnos, valoración general…)
- Cuando conoces que hay un estudiante con discapacidad en tu asignatura ¿qué medidas tomas? ¿qué pasos sigues?
- En cuanto a tu experiencia con estudiantes con discapacidad, ¿qué es lo que más te ha gustado? ¿y lo que menos? ¿Con qué dificultades te has encontrado a la hora de trabajar con estos estudiantes?

Si tuvieras que aconsejar a algún profesor o profesora que por primera vez tiene un alumno o una alumna con discapacidad ¿qué recomendaciones les darías?

- Cuando tuviste por primera vez a un alumno o alumna con discapacidad en el aula, ¿recibiste algún consejo o recomendación? En caso afirmativo, ¿cómo te ayudó?

**CREENCIAS**

**CONCEPCIONES SOBRE DISCAPACIDAD**

- Cuando hablamos de discapacidad, ¿qué ideas te vienen a la cabeza?
- ¿Qué crees que es lo que te ha llevado a interesarse y a trabajar por la inclusión de los estudiantes con discapacidad?

**ROL DOCENTE Y ACTITUDES**

- ¿Podrías decirme algunas características que te definan como docente?
- ¿Cómo crees que te ven tus estudiantes?
- ¿Cómo crees que influye tu actitud o características en el aprendizaje del alumnado?
- ¿Cuáles crees que son las características (personales, profesionales) y actitudes fundamentales que debe tener el profesorado que desarrolla educación inclusiva y da una respuesta educativa de calidad al alumnado, con y sin discapacidad?

**FORMACIÓN DEL PROFESORADO**

- ¿Crees que es necesario que el profesorado se forme para un mejor aprendizaje de los estudiantes? ¿Por qué?
- ¿Qué formación consideras que deberías tener para enseñar al alumnado adecuadamente y en especial, al alumnado con discapacidad?
- ¿Crees que el trabajo en equipo es necesario para atender adecuadamente a los estudiantes con discapacidad? ¿Por qué?

**PROCESO DE ENSEÑANZA Y APRENDIZAJE**

- Desde tu punto de vista, ¿qué es lo más importante para que el alumnado pueda aprender adecuadamente?
- ¿Crees que todo el alumnado aprende de la misma forma? ¿Crees que existen diferencias entre cómo aprende el alumnado con discapacidad y sin discapacidad?, ¿por qué?
- ¿Crees que en el caso de implementar prácticas inclusivas se beneficiarían todos los estudiantes o solo los estudiantes con discapacidad?, ¿por qué?

**ADAPTACIONES CURRICULARES**

- ¿Qué opinas sobre la necesidad de realizar adaptaciones en su asignatura para que los estudiantes con discapacidad puedan aprender?
- ¿Qué te supone como docente adaptar una asignatura para hacerla inclusiva (exigencia de la materia, trato de favor, tiempo y esfuerzo docente, dificultad de adaptación de materiales…)?

**UNIVERSIDAD**

- ¿Crees que la universidad favorece la educación inclusiva, dando oportunidades para que todo el alumnado pueda aprender y participar en condiciones de igualdad?, ¿por qué?, ¿cómo lo hace?
- ¿Crees que la propia universidad en un momento dado, puede suponer una barrera para que las personas con discapacidad puedan estudiar una carrera y finalizar sus estudios con éxito? ¿Por qué
- ¿Qué crees que supone para una persona con discapacidad tener la oportunidad de aprender en la universidad?
- ¿Qué crees que puede aportar el alumnado con discapacidad a la universidad?
- ¿Qué recomendaciones harías a la universidad para que fuera más inclusiva, dando una respuesta de calidad a todo el alumnado?

**CONOCIMIENTOS**

**CONOCIMIENTO GENERAL SOBRE DISCAPACIDAD Y EDUCACIÓN INCLUSIVA**

- ¿Qué sabes sobre la discapacidad?
- ¿Qué barreras crees que los estudiantes con discapacidad se encuentran cuando estudian en universidad? ¿Y qué ayudas?
- ¿Qué conoces sobre la educación inclusiva?

**CONOCIMIENTOS ESPECÍFICOS DISCAPACIDAD**

- ¿Sabes si existe normativa universitaria sobre discapacidad? ¿Qué conoces de la normativa universitaria en materia de discapacidad y los derechos de estos estudiantes en la universidad?
- ¿Qué servicios y recursos conoces que pone a disposición su universidad para ayudar al alumnado con discapacidad?
- ¿Qué tipos de discapacidades crees que podemos encontrar en un aula universitaria?
- ¿Qué conoces sobre las necesidades y apoyos que pueden requerir estos estudiantes?
- estudiantes?
- ¿Qué conoces del concepto de diseño universal de aprendizaje?

**CONOCIMIENTOS ESPECÍFICOS PROCESOS DE ENSEÑANZA Y APRENDIZAJE**

- Como docente, ¿qué estrategias de enseñanza conoces que motivan y favorecen más el aprendizaje de los estudiantes?, ¿podrías describir algunas de ellas?

**FORMACIÓN**

- ¿Sueles participar en cursos/programas de formación continua?, ¿por qué?
- ¿Qué formación tienes como docente? (valoración)
- ¿Qué recomendaciones harías para mejorar la formación del profesorado en materia de atención al alumnado con discapacidad?

¿Deseas añadir alguna cuestión o información que no haya sido incluido en esta entrevista?

**GUIÓN ENTREVISTA. DISEÑOS Y ACCIONES**

Proyecto: Pedagogía inclusiva en la universidad: narrativas del profesorado
(MINECO, ref. [EDU2016-76587-R](https://investigacion.us.es/sisius/sis_proyecto.php?idproy=27540))

**OBJETIVO:** Describir y explicar los diseños y acciones del profesorado universitario para desarrollar una pedagogía inclusiva en la universidad.

**Diseño:** consideraciones tenidas en cuenta en la planificación de un proyecto docente accesible.

**Acciones:** estrategias afectivas, emocionales y de enseñanza y aprendizaje, puestas en práctica para el desarrollo de una pedagogía inclusiva.

**DISEÑO**

- A la hora de diseñar el proyecto docente de tu asignatura ¿qué consideraciones de partida tienes? ¿Cuáles son los aspectos fundamentales a los que prestas más atención?
- ¿El proyecto docente se hace en colaboración con otros compañeros del departamento? En caso afirmativo preguntar por qué.
- ¿Cuál o cuáles consideras que son los elementos más importantes a la hora de elaborar un proyecto docente en relación al alumnado con discapacidad?
- ¿En tu proyecto docente incluyes alguna información específica dirigida al alumnado con discapacidad? Si es así, ¿qué tipo de información?
- ¿Crees que cualquier alumno con discapacidad podría cursar tu asignatura tal y como está diseñada? ¿Por qué?
- ¿Requeriría algún tipo de modificación? En caso afirmativo, ¿cuáles serían? ¿Con qué dificultades te podrías encontrar para realizar estas modificaciones?
- ¿Sueles tener en cuenta la opinión del alumnado para rediseñar el proyecto docente durante el desarrollo de la asignatura? ¿y para el próximo curso académico? En caso afirmativo, ¿cómo recoges estas opiniones?
- Si algún profesor/a te pidiese opinión para que su proyecto docente fuera inclusivo y tuviera en cuenta a todo el alumnado, incluido el alumnado con discapacidad, ¿Qué le recomendarías?

**ACCIONES**

**PROCESO DE ENSEÑANZA Y APRENDIZAJE EN GENERAL**

- Ya hemos hablado sobre la planificación de un proyecto docente, en este bloque de preguntas estamos interesados en cómo es tu puesta en práctica: ¿qué aspectos consideras que son los más importantes (organización y disposición del aula, buen funcionamiento de dispositivos electrónicos, buena iluminación y acústica en el aula, metodología, etc.) para desarrollar prácticas de educación inclusiva, es decir, para que pueda aprender y participar todo el alumnado?
- Nos gustaría ponernos en situación y que nos contaras cómo es una clase tuya, desde que se inicia hasta que finaliza. (la clase que quiera, teórica, práctica…)
- ¿Qué haces para conocer cuáles son las necesidades o dificultades concretas que un estudiante tiene para seguir con éxito tu asignatura? ¿Qué haces para ayudarle a superarlas?
- Si le pudiéramos preguntar a un alumno qué mejoraría de tus clases, ¿qué crees que diría?
- Desde tu papel como docente: ¿cuáles consideras que son las principales dificultades a las que te enfrentas al ayudar a aprender al alumnado y en especial al alumnado con discapacidad?

**OBJETIVOS PROYECTO DOCENTE**

¿crees que todos tus estudiantes pueden alcanzar los objetivos de aprendizaje? ¿Por qué?

- En caso de tener algún estudiante con discapacidad, ¿planteas para ellos objetivos de aprendizaje diferentes? ¿Por qué?

**MATERIALES/RECURSOS**

- ¿Haces uso de diferentes recursos para presentar los contenidos (textos impresos, medios tecnológicos, presentaciones, vídeos…)? ¿Por qué?
- ¿Cómo pones a disposición de los estudiantes los recursos a utilizar en clase (copistería, plataforma virtual, entrega en clase, por correo electrónico, búsqueda por parte del alumno…)? ¿Por qué utiliza esa/s vía/s?
- ¿En qué momento facilitas el acceso a los materiales (¿antes de que empiece la asignatura, el mismo día…?)

**PLATAFORMA Y RECURSOS TECNOLÓGICOS**

- ¿En el desarrollo de tu asignatura utilizas la enseñanza virtual y/o recursos tecnológicos?, ¿por qué? ¿para qué?
- ¿Qué influencia tiene estos recursos en el aprendizaje de los estudiantes y en especial, de los estudiantes con discapacidad?

**ESTRATEGIAS METODOLÓGICAS**

- ¿Cuál es la metodología docente que utilizas para enseñar, es decir, cómo das clases? ¿Por qué?
- De las metodologías docentes que utilizas, ¿cuáles consideras que son más efectivas para que todo el alumnado aprenda y por qué? ¿y para los estudiantes con discapacidad?

**RELACIÓN PROFESORADO-ALUMNADO**

¿Para ti es importante la relación que se establece entre alumnado y profesorado? ¿Por qué?

- ¿Qué prácticas/ estrategias llevas a cabo para fomentar la relación con tu alumnado?
- ¿Cómo crees que la relación con los alumnos puede influir en el aprendizaje del alumnado?

**MOTIVACIÓN ESTUDIANTES**

- Desde tu punto de vista, ¿cuáles consideras que son piezas clave para conseguir un alumnado implicado y motivado durante todo el proceso de enseñanza-aprendizaje?
- ¿Cuál(es) piensas que son las causas más frecuentes de la desmotivación de tu alumnado? ¿por qué?
- ¿A qué recursos sueles acudir cuando ve que se está produciendo un descenso en la motivación e implicación de tu alumnado?
- En cuanto al alumnado con discapacidad, ¿haces cosas distintas para motivarlos? ¿Por qué? En caso afirmativo, ¿cómo lo haces?

**EVALUACIÓN**

¿Cómo evalúas? ¿La evaluación es la misma para todos tus estudiantes o haces algún tipo de cambio o adaptación para los estudiantes con discapacidad? ¿Por qué?

Si has adaptado alguna vez la evaluación ¿en qué ha consistido dicha adaptación? Y si no lo has hecho, ¿te has planteado alguna vez algún cambio en la evaluación? ¿Por qué?

**ACCIÓN TUTORIAL**

- ¿Cómo organizas las tutorías?
- ¿Cómo es la participación del alumnado en las tutorías? ¿Encuentra diferencias en la participación del alumnado con discapacidad?, ¿a qué crees que se debe?

**ADAPTACIONES CURRICULARES**

- ¿Realizas alguna modificación de la asignatura cuando hay un estudiante con discapacidad?, ¿por qué? En caso afirmativo: ¿qué tipo de modificaciones ha realizado? ¿por qué?
- Si alguna vez has necesitado ayuda en relación a algún tipo de modificación para estudiantes con discapacidad ¿a quién has acudido? ¿Cómo valoras la ayuda que te dieron?
- ¿Alguna vez has tenido que colaborar con algún compañero o compañera de tu departamento para realizar alguna modificación a un estudiante con discapacidad en una asignatura compartida? Describir lo que se ha hecho

**Para finalizar esta entrevista, nos gustaría saber ¿cómo te ha influido, a nivel personal y profesional, haber tenido estudiantes con discapacidad en el aula?**

¿Deseas añadir alguna cuestión o información que no haya sido incluido en esta entrevista?
