# Supplementary material for: Is pedagogical training an essential requirement for inclusive education? The case of faculty members in the area of Social and Legal Sciences in Spain
Source: PLoS One. 2021 Jul 2;16(7):e0254250. doi: 10.1371/journal.pone.0254250 (PMC8253417; doi:10.1371/journal.pone.0254250)
Supplement: S1 File — (ZIP) [file pone.0254250.s001.zip › 1.1 DISCAPACIDAD (1).rtf]

Documento:		4. Ciencias Sociales y Jurídicas\P1 CCSS Creencias
Peso:	0
Posición:	22 - 23
Código:	1. Creencias\Concepciones discapacidad\1.1. Discapacidad
E: Bien. Y entrando en el apartado sobre las creencias, cuando hablamos de discapacidad, ¿a ti qué ideas se te vienen a la cabeza?
P1: Bueno, al principio…ya te digo, que como he hecho muchos cursos, pues ya tengo alguna información acumulada, pero al principio, uno cuando piensa en discapacidad, pues se le viene a la cabeza la típica discapacidad física, ¿vale? Pero luego yo sé que hay otro tipo de discapacidades que no se ven, entonces, por eso es muy importante que el primer día de clase todo el alumnado sepa que tú estás dispuesto a escuchar y a ayudar. Y que el alumno pueda expresarse en público si lo desea o en privado sobre su circunstancia y ver en qué medida el profesor y la asignatura se pueden adaptar a esas circunstancias. Eso es fundamental para mí.


Documento:		4. Ciencias Sociales y Jurídicas\P2 CCSS Creencias
Peso:	0
Posición:	26 - 27
Código:	1. Creencias\Concepciones discapacidad\1.1. Discapacidad
E: Totalmente. Y luego P2, cuando hablamos de discapacidad, a ti qué ideas se te vienen a la cabeza.
P2: A mí es que no me gusta la palabra discapacidad, de hecho, es una de las cuestiones que te iba a comentar al respecto y se lo comenté también a la psicóloga del SACU, y es que hablar de discapacidad, desde mi punto de vista es algo completamente desfasado y, además, estigmatizador. De hecho, hay un tema que yo he trabajado mucho que es el estatuto de la víctima del delito, y en esta directiva se establece algo que es fundamental, y es que habla de necesidades específicas de protección. Eso es lo correcto, hay personas con necesidades específicas, el legislador de España, de todas formas, no es muy claro porque habla de personas con necesidades especiales, y otras con necesidades específicas. Tanto hablar de personas discapacitadas como de personas con necesidades especiales, es una forma de estigmatizarla. La gran conquista o la gran ventaja es personas con necesidades específicas, porque es que lo puede ser cualquier persona. Tenemos que tener en cuenta que cualquier persona puede tener en un momento dado necesidades específicas tenga o no tenga algún tipo de patología. Y en segundo lugar, que son características de las personas, luego, son necesidades específicas. No me gusta hablar de personas discapacitadas porque es estigmatizador. De hecho, cuando hablamos nosotros en términos técnicos y más avanzados, no hay que hablar tampoco de personas discapacitadas, sino de personas con la capacidad judicialmente limitada, eso sería una cuestión diferente. No sé si te ha parecido bien este planteamiento…cualquier persona puede tener necesidades específicas de protección, cualquier persona.


Documento:		4. Ciencias Sociales y Jurídicas\P3 CSS Creencias
Peso:	0
Posición:	20 - 21
Código:	1. Creencias\Concepciones discapacidad\1.1. Discapacidad
E: En relación ahora sobre las concepciones sobre discapacidad. Cuando escuchas la palabra discapacidad, ¿qué ideas se te vienen a la cabeza? 
P3: Inicialmente, se me viene una discapacidad física o psicológica y creo que hay otras discapacidades, pero yo creo que la discapacidad no tiene que entenderse como algo negativo, sino que es un problema, entre comillas que, si no tiene solución, tiene cierta adaptación. Yo no entiendo a los discapacitados como excluidos de la sociedad. Yo no lo veo así. Veo que tienen unas capacidades diferentes, pero que es solucionable.


Documento:		4. Ciencias Sociales y Jurídicas\P4 CCSS Creencias
Peso:	0
Posición:	24 - 25
Código:	1. Creencias\Concepciones discapacidad\1.1. Discapacidad
E: Y ahora pasamos a la parte de creencias sobre discapacidad. Cuando hablamos de “discapacidad”, qué ideas se te vienen a la cabeza.
P4: Se me vienen, pues personas que tienen que afrontar en su día a día muchos obstáculos.


Documento:		4. Ciencias Sociales y Jurídicas\P5 CSS Creencias
Peso:	0
Posición:	28 - 29
Código:	1. Creencias\Concepciones discapacidad\1.1. Discapacidad
E: Estupendo. Bien. Vamos a pasar ahora a una serie de preguntas del bloque que llamamos nosotros de creencias, para saber qué crees tú o cuál es tu idea sobre ciertos conceptos. En primer lugar, tu idea sobre la discapacidad. Cuando hablamos sobre discapacidad, qué ideas se te vienen a la cabeza.
P5: Es que me pongo muy políticamente correcto, pero discapacidad, a lo bruto…alguien tiene una discapacidad cuando tiene alguna característica que le impide hacer ciertas cosas que la mayoría en sus condiciones, no sé si de edad o de formación podría hacer, ¿no? Discapacidad es impedimento, ¿no? Es dificultad, es obstáculos, necesidad de atención…


Documento:		4. Ciencias Sociales y Jurídicas\P6 CCSS Creencias
Peso:	0
Posición:	12 - 13
Código:	1. Creencias\Concepciones discapacidad\1.1. Discapacidad
E: Vale. Entonces, en el caso de no haber tenido o de no ser consciente de haber tenido alumnado con discapacidad en su aula, vamos a pasar directamente al bloque de creencias y, en primer lugar, te voy a hacer una serie de preguntas sobre lo que tú crees que es la discapacidad. Cuando hablamos de discapacidad, ¿qué ideas se te vienen a la cabeza? 
P6: Pues cuando hablo de discapacidad se me vienen a la cabeza personas que pueden requerir algún tipo de esfuerzo añadido a la hora de enfrentarse a un proceso de aprendizaje y, se me viene un concepto muy amplio sobre discapacidad. A lo mejor, lo primero que se me viene es darnos cuenta de que alguien tiene una discapacidad física, pero no es solo el concepto sobre discapacidad física que, seguramente, sería lo más fácil de percibir, sino que hay un montón de discapacidades que no tienen nada que ver con tener alguna discapacidad física y que pueden limitar ese proceso de aprendizaje o hacerlo más lento que los alumnos que no la tienen. Ese es mi concepto.


Documento:		4. Ciencias Sociales y Jurídicas\P7 CCSS Creencias
Peso:	0
Posición:	28 - 29
Código:	1. Creencias\Concepciones discapacidad\1.1. Discapacidad
E: Cuando, hablamos un poco ahora sobre las concepciones que tenemos ahora sobre la discapacidad. Cuando yo le comento o le hablo sobre discapacidad, ¿qué idea tiene por discapacidad?
P7: Pues que, en alguno de los sentidos, pues que tiene alguna dificultad. Discapacidad a lo mejor para poder atender o discapacidad sensorial, discapacidad en cuanto a que puedan tener problemas para estudiar, para concentrarse, motora también. Un alumno que le costaba mucho escribir, yo entiendo la discapacidad como eso, poseer una cierta dificultad con respecto a lo que es el estándar. Eso para mí es eso, pero no tiene por qué tener una dificultad para seguir una carrera.


Documento:		4. Ciencias Sociales y Jurídicas\P8 CCSS Creencias
Peso:	0
Posición:	76 - 85
Código:	1. Creencias\Concepciones discapacidad\1.1. Discapacidad
E: Y tú, ¿qué ideas tienes sobre la discapacidad? Cuando yo te digo discapacidad, ¿en qué piensas?
P8: Pues mira, hasta que conocí a este chico, entendía discapacidad o lo asociaba a discapacidades más físicas que psíquicas. Me planteaba cómo actuaría con una persona que no pudiese leer, con un sordomudo con una minusvalía en las piernas, yo que sé. Tengo una amiga que además está licenciada aquí, se licenció en Derecho. Es amiga muy personal y tenía poliomielitis de la época nuestra de los sesenta, que te quedas en una silla de ruedas. Entonces, cuando yo tenía en la mente discapacidad era eso, más física que psíquica, no me había planteado discapacidades en sentido psicológico o psiquiátrico, o sea, no sé si, me imagino que, por ejemplo, un nivel de Síndrome de Down pequeño te permite llegar a graduarte, que podría ser ¿no? Por ejemplo, mi cercanía con personas que tienen Síndrome de Down, que la tengo a nivel familiar, pues son personas que tienen un Síndrome de Down muy elevado, ¿no? Entonces, es muy difícil la comunicación con ellas. Hay días que te adoran y te dan besos o no sé qué y hay días que están encerradas en su mundo y no quieren saber nada de nadie. Yo intento aproximarme o entablar una conversación o interesarme y me doy cuenta de que actúo mal porque hago como teatro, ¿sabes? No sé cómo decirte, es una persona que tiene cuarenta y muchos años. Tiene una longitud de vida muy amplia frente a lo que es normal y yo la trato como un bebé, como si fuese un niño. Mi hijo el mayor es muy sensible a estos temas, me dice “pero es que le hablas a la prima Laura como si estuvieses hablando con un bebé y Laura no es un bebé”, “ya, pero es que no sé, me sale así”. Y ahí, por ejemplo, me encuentro muchas veces que es…
E: Como una barrera.
P8: Como un muro, porque ella no quiere. Y otros días que me ve, me abraza y me lleva y me enseña “te voy a enseñar mi nuevo cuarto, lo que he pintado” y otros días que no quiere saber nada. Pero no sé cómo, porque es un Síndrome de Down con un alto grado, ¿no? Pero me imagino que en menor grado se puede llegar…y entonces no sé cómo me enfrentaría a una clase, ni sé si ese tipo de perfil crea más…situación diferencial en el aula, porque al fin y al cabo este chico…
E: Nadie supuso nada nunca.
P8: No, pasó desapercibido.
E: Y algo más manifiesto quieres decir tú, con más evidencia externa.
P8: Claro, con más evidencia externa, lo localizas físicamente.
E: Y sabes quién es la persona con discapacidad en el aula.
P8: Eso es. Entonces, esto es, digamos se vivió internamente conmigo como profesora, me imagino que también con el resto de profesores del grupo, pero no lo sé, lo desconozco, pero no trascendía en la clase y otras minusvalías sí pueden trascender.


Documento:		4. Ciencias Sociales y Jurídicas\P9 CCSS Creencias
Peso:	0
Posición:	28 - 29
Código:	1. Creencias\Concepciones discapacidad\1.1. Discapacidad
E: Cuándo hablamos de discapacidad, ¿qué ideas se te vienen a la cabeza? 
P9: Pues, una limitación y puede ser de muchos tipos. Y por eso digo que todos somos discapacitados porque, por ejemplo, a uno le puede costar trabajo hablar en público.


Documento:		4. Ciencias Sociales y Jurídicas\P10 CCSS Creencias
Peso:	0
Posición:	23 - 24
Código:	1. Creencias\Concepciones discapacidad\1.1. Discapacidad
E: Y, si empezamos a hablar un poquito de las creencias y de…en cuanto, por ejemplo, a la discapacidad. Cuando yo te digo discapacidad, a ti, ¿qué ideas se te vienen a la cabeza?
P10: Pues, no sé, pues entiendo yo que, probablemente, respecto a los estándares de normalidad, entre muchas comillas, pues, que hay un desajuste en algún aspecto que puede ser muy variado, entiendo yo. Eso es lo que entiendo.


Documento:		4. Ciencias Sociales y Jurídicas\P11 CCSS Creencias
Peso:	0
Posición:	27 - 28
Código:	1. Creencias\Concepciones discapacidad\1.1. Discapacidad
E: Claro. Y pasando a las concepciones que tienes acerca de la discapacidad. Cuando hablamos de discapacidad, qué ideas se te vienen a la cabeza.
P11: Pues mira, yo tengo digamos una concepción social, digamos, solidaria. Yo colaboraba con ONG del desarrollo y creo mucho en la igualdad de oportunidades. Y como cada uno dependiendo de donde ha nacido tiene más o menos oportunidades sin tener la culpa de nada, pues lo mismo opino con las personas que tienen una discapacidad, no tienen la culpa de tenerla. Entonces, la sociedad ha de proteger esto. Entonces, yo esto trato de verlo de la siguiente manera…claro esto también lo hace mucho la madurez personal, de gente que has ido conociendo por el camino, ¿no? Te das cuenta de que en realidad todos somos imperfectos y todos tenemos discapacidades, lo que pasa es que unas se notan más y otras menos. Yo prefiero verlo así, porque claro, eso de que “esto es una persona normal y esto una persona discapacitada”, eso no va conmigo, eso me repele a horrores, no puedo con eso. Entonces, mi actitud es de que esa persona tiene una tara como yo tengo otra. Y esa que tiene esa persona, le podemos ayudar a reducirla o a que no le afecte tanto como si nadie lo atiende. 


Documento:		4. Ciencias Sociales y Jurídicas\P12 CCSS Creencias
Peso:	0
Posición:	26 - 27
Código:	1. Creencias\Concepciones discapacidad\1.1. Discapacidad
E: Cuando hablamos de discapacidad, ¿qué ideas se te vienen a la cabeza?
P12: Pues, no lo sé la verdad no te sabría decir. Es alguien que tiene una serie de dificultades para realizar ciertas cosas. Esto es lo que se me ocurre a mí ahora.


Documento:		4. Ciencias Sociales y Jurídicas\P13 CCSS Creencias
Peso:	0
Posición:	25 - 26
Código:	1. Creencias\Concepciones discapacidad\1.1. Discapacidad
E: Y cuando hablamos de discapacidad, qué ideas se te vienen a la cabeza.
P13: Cuando yo hablo de discapacidad, supongo que por la educación, se me viene fundamentalmente la discapacidad física, pero a lo largo del tiempo he podido ver que hay otros tipos, que yo no sé si le llamaría discapacidad, le llamaría diversidad, son estudiantes o personas con otro tipo de necesidades especiales…porque a lo mejor tienen carencias psíquicas, pero luego te puedo asegurar que tienen otro tipo de habilidades que suplen completamente eso.


Documento:		4. Ciencias Sociales y Jurídicas\P15 CCSS Creencias
Peso:	0
Posición:	28 - 29
Código:	1. Creencias\Concepciones discapacidad\1.1. Discapacidad
E: Pasamos a la parte de las creencias. Cuando hablamos de discapacidad, ¿qué ideas se le vienen a la cabeza?
P15: Pues todo tipo de discapacidades, o bien físicas o bien psicológicas.


Documento:		4. Ciencias Sociales y Jurídicas\P17 CCSS Creencias
Peso:	0
Posición:	40 - 43
Código:	1. Creencias\Concepciones discapacidad\1.1. Discapacidad
E: Vamos a entrar un poquito en concepciones de discapacidad. Cuando hablamos de discapacidad, ¿qué ideas se te vienen a la cabeza?
P17: A mí se me viene más a la mente el discapacitado psíquico que el físico. Siempre.
E: También por tu propia experiencia.
P17: También por mi propia experiencia. A mí se me vienen a la mente los gravemente afectados, que están en residencias, por ejemplo. Esos son los que se me vienen a la mente cuando pienso en discapacidad. Cuando yo era bastante más joven, trabajé en una asociación de prominusválidos físicos y teníamos hasta empresas en las que trabajaban los chicos. Entonces, cuando hablamos de discapacidad, se me viene a la mente eso, los que estaban gravemente afectados que, a lo mejor, estaban haciendo fregonas en un centro o estaban rellenando los estuches de colores, o ensobrando cosas.


Documento:		4. Ciencias Sociales y Jurídicas\P18 CCSS Creencias
Peso:	0
Posición:	36 - 37
Código:	1. Creencias\Concepciones discapacidad\1.1. Discapacidad
E: Bueno, vamos a pasar a otro bloque de concepciones, ¿no? Cuando hablamos de discapacidad, ¿qué ideas se te vienen a la mente?
P18: Bueno, a mí se me viene cualquier tipo de discapacidad física o psíquica, cualquiera, porque es que yo estoy dando este año ordenación en el empleo, y hablamos de la cuota de los discapacitados en el empleo y, entonces, obviamente, es cualquier tipo de discapacidad, no se me viene nadie en concreto, sino cualquier tipo de discapacidad, que, a lo mejor, no le impide ni siquiera para la realización de las clases, porque el hecho, por ejemplo, de venir a clase en silla de ruedas, pues no le impide, se puede adaptar.


Documento:		4. Ciencias Sociales y Jurídicas\P19 CCSS Creencias
Peso:	0
Posición:	22 - 23
Código:	1. Creencias\Concepciones discapacidad\1.1. Discapacidad
E: Vale, ya hemos acabado con la introducción. Vamos a pasar a conocer tus creencias. Cuando hablamos de discapacidad, ¿qué ideas te vienen a la cabeza?
P19: Pues, una persona que tiene unas dificultades, dependiendo de la discapacidad, para realizar determinadas cosas, pero nada más.


Documento:		4. Ciencias Sociales y Jurídicas\P21 CCSS Creencias
Peso:	0
Posición:	40 - 41
Código:	1. Creencias\Concepciones discapacidad\1.1. Discapacidad
E: Estupendo. Nos pasamos ya al primer bloque de creencias. Cuando hablamos de discapacidad, ¿qué es lo primero que se le viene a la cabeza?
P21: Yo, un poco lo que te decía antes, para mí, la discapacidad, es una carencia de, y, entonces, a partir de ahí, pues, evidentemente, pues ya sea de un sentido, de la vista o del oído, que hemos tenido casos de alumnos con sordera o carencia de movilidad en un grado u otro por problemas físicos. Y ya está, como te digo es algo que viene por carencia de. Hay otros que tienen carencias que no son físicas y que las considero, incluso, más importantes, pero, también por la experiencia que tengo, ya no solo en el aula, sino en el entorno, incluido en el familiar, que tengo familia con discapacidad, que…de discapacidad, me viene, por un lado, carencia de, y por otro, la superación, precisamente por esa carencia tan clara y evidente, el concepto de superación, de ser personas que están batallando continuamente. Porque vivimos en una sociedad, que por regla general, la desgracia no es la discapacidad, la desgracia es cómo se atiende la discapacidad, porque, en un momento determinado, no digo que se hable, pero muy bien, si eres ciego, eres ciego, como si eres sordo o si tienes un problema de parálisis, pero una vez que lo tiene el problema, no creo que sea tanto eso como que si tienes problemas físicos no puedas moverte por la ciudad y ese tipo de cosas, ¿no? Y ahí sí que…yo tengo una amiga en silla de ruedas, y que tú en un momento determinado te plantees ir a un espectáculo al que ella sabe ya de entrada que no puede ir porque la sala no está acondicionada, a mí esas cosas me parecen terribles, ¿no?


Documento:		4. Ciencias Sociales y Jurídicas\P22 CCSS Creencias
Peso:	0
Posición:	22 - 23
Código:	1. Creencias\Concepciones discapacidad\1.1. Discapacidad
E: Vale, pues continuamos con la parte de creencias. Cuando hablamos de discapacidad, ¿qué ideas se le vienen a la cabeza?
P22: Un alumno que tenga limitaciones de diferente tipo, físico…psíquico, normalmente, aquí a la universidad no llegan, pero sí que físico, evidentemente…como, normalmente, he tenido ciegos o con problemas de visión, una visión escasa, es, normalmente, el tipo de personas en las que yo pienso, ¿no?


Documento:		4. Ciencias Sociales y Jurídicas\P22 CCSS Creencias
Peso:	0
Posición:	47 - 47
Código:	1. Creencias\Concepciones discapacidad\1.1. Discapacidad
 Entonces, desde ese punto de vista, el alumno que tiene discapacidad, puede tener limitaciones en determinadas cuestiones, pero puede haber desarrollado otras. Entonces, en ese sentido, yo creo que sí, que el trabajo en equipo es bastante bueno. 


Documento:		4. Ciencias Sociales y Jurídicas\P23 CCSS Creencias
Peso:	0
Posición:	28 - 31
Código:	1. Creencias\Concepciones discapacidad\1.1. Discapacidad
E: Vale. Empezando con el primer bloque de creencias y concepciones sobre discapacidad, cuando hablamos de discapacidad, ¿qué ideas le vienen a la cabeza, P23?
P23: ¿De discapacidad?
E: Sí. ¿Qué es lo primero que piensa?
P23: Dificultad, reto, complejidad... Sobre todo, alguien que necesita ayuda, alguien con mucha voluntad. Me resultan personas muy valiosas porque podrían estar derrotadas en casa, sin embargo, están aquí. Entonces, me parece alguien... Por un lado, un reto, una dificultad y por otro lado alguien con mucho valor para estar haciendo las cosas y estar teniendo un tesón y una fuerza que a lo mejor no tenemos los que tenemos todas nuestras capacidades físicas porque no lo hemos valorado.


Documento:		4. Ciencias Sociales y Jurídicas\P24 CCSS Creencias
Peso:	0
Posición:	20 - 21
Código:	1. Creencias\Concepciones discapacidad\1.1. Discapacidad
E: Y ahora pasamos a hablar un poco acerca de las creencias sobre discapacidad. Cuando hablamos de discapacidad, qué ideas se te vienen a la cabeza.
P24: Pues conceptualmente, como tú has ido escuchando, he ido utilizando los dos términos, discapacidad y diversidad funcional, ¿por qué? Pues porque ahora mismo están conviviendo. Principalmente, bueno, sí o sí, en la universidad complutense hemos apostado por diversidad funcional. Por qué diversidad funcional, pues porque el lenguaje crea realidades, vamos a partir de ahí. Entonces, si el lenguaje crea realidades, qué está sucediendo. Dis es un prefijo que disminuye las capacidades, y no es verdad. Todos y cada uno de los ciudadanos…quién tiene el 100% en todo, vamos, me encantaría conocer a esa persona. Vamos, no es verdad. Lo que pasa, que o bien están ocultas o bien están normalizadas, y te hablo por mí. Yo tengo miopía, y tengo ocho, y es como yo le digo a mis estudiantes “yo tengo la gran suerte de que ha habido profesionales técnicos científicos que me han facilitado la vida y ya no soy considerada una persona con diversidad funcional visual. Sin embargo, si no existieran las lentillas, yo no estaría aquí ahora mismo. Por lo tanto, ustedes para ser profesionales tenéis que llevar en las mochilas estos conocimientos, porque vais a ser lo que vais a estar en recursos humanos, los que vais a trabajar en ciertas áreas y vais a tener como compañeros a personas con diversidad o un usuario. Y le tenéis que tratar con la máxima normalidad”. Entonces, sí que hemos tenido cartas…a ver, no quiero utilizar la palabra amenazante, porque no quiero que llegue ahí, pero cuando ya la universidad tomó esta decisión, organismos representantes de la ciudadanía, nos escribieron una carta diciendo que ese término no teníamos que utilizarlo. Alegando, muy bien alegado, a nivel estatal, autonómico, todos los temas, ¿no? Y su argumento tiene parte de razón, no se le puede discutir, pero hay un grupo de ciudadanos, aunque no están constituidos como asociación o como tal, que han decidido utilizar este nombre, ¿no? El nombre de diversidad funcional. Y nosotros como trabajadores sociales, en mi caso, consideramos que tenemos que escuchar, como hemos hablado al principio, las historias de vida, hay que darles voz y escucharles a ellos y saber qué es lo que ellos quieren. Porque el nombre de discapacidad, ¿quién lo ha puesto?


Documento:		4. Ciencias Sociales y Jurídicas\P24 CCSS Creencias
Peso:	0
Posición:	22 - 23
Código:	1. Creencias\Concepciones discapacidad\1.1. Discapacidad
E: El resto, nosotros.
P24: Las personas sin diversidad y específicamente, el área de conocimiento de la salud. Entonces, yo digo varias cuestiones: hay una construcción social y otra médica. Bueno, hay más, pero vamos a centrarnos en las dos grandes áreas. Que estamos en la médica, vale, vamos a utilizar ese término porque ahí se usan esos términos en salud, qué es lo que le falta, qué es lo que no tiene, pero igual que cuando vas al dentista y te dice que tienes una caries o te falta un diente. Siempre se utilizan términos de faltas, de lo que hay que reparar, rehabilitar, diagnosticar, tratamientos… Pero en el plano social, no es lo que falta, no estamos hablando del organismo, estamos hablando de competencias que puede desarrollar la persona a nivel intelectual, físico o lo que sea.


Documento:		4. Ciencias Sociales y Jurídicas\P24 CCSS Creencias
Peso:	0
Posición:	23 - 23
Código:	1. Creencias\Concepciones discapacidad\1.1. Discapacidad
Como institución que me debo a mi institución y a las leyes que hay, pero como bien sabemos, las leyes siempre van por detrás de los acontecimientos o los avances, y cuesta lo que cuesta. Pero ya están en ello, de hecho, ya no es un grado de minusvalía, ese término ya ha desaparecido, es un grado de discapacidad cuando se les reconoce. Es decir, se están haciendo avances, lo que pasa que en ciertas áreas es muy lento. 


Documento:		4. Ciencias Sociales y Jurídicas\P24 CCSS Creencias
Peso:	0
Posición:	39 - 39
Código:	1. Creencias\Concepciones discapacidad\1.1. Discapacidad
Son personas como una más, pero con unas capacidades diferentes de superación, en positivo, no en negativo como normalmente las vemos.


Documento:		4. Ciencias Sociales y Jurídicas\P25 CCSS Creencias
Peso:	0
Posición:	38 - 41
Código:	1. Creencias\Concepciones discapacidad\1.1. Discapacidad
E: Muy bien. Vale, pues vamos a entrar en el bloque de creencias, ¿vale? Principalmente, vamos a entrar en las concepciones que tienes sobre la discapacidad. De manera muy general, cuando a ti te hablan de discapacidad, del concepto de discapacidad, ¿qué ideas se te vienen a ti a la cabeza? 
P25: Espontáneamente ¿no?, pues me vienen a la cabeza, aquellas personas que tienen algún tipo de dificultad para ser autónomos, para poder ser independientes en la vida.
E: Muy bien. ¿Los que  necesitan algún tipo de ayuda o de apoyo?
P25: Sí, los que tienen algún tipo de freno, o algún tipo de obstáculos, ya sea físico, sensorial, o alguna dificultad mental, todo lo que te impida desarrollarte. 


Documento:		4. Ciencias Sociales y Jurídicas\P25 CCSS Creencias
Peso:	0
Posición:	130 - 139
Código:	1. Creencias\Concepciones discapacidad\1.1. Discapacidad
E: ¿Alguna otra cuestión o información que quieras añadir?
P25: No tengo ni idea ahora mismo. ¿Sabes a quién tengo continuamente en mi cabeza, ahora mismo? pues, este chico que es Síndrome de Down que ha hecho magisterio, que ha sido actor…pues el otro día salió en la televisión…ha hecho otra carrera también, que es simpatiquísimo. Su hermano tiene una carrera y él dijo “pues si tú tienes, yo también tengo”. Y yo me planteo, este chico y con esta mente y sinceramente, he pensado a veces, ¿a este chico se le ha regalado? No vamos a negar, que una siempre tiene prejuicios. Mira, otro elemento, trabajar los prejuicios.
E: No creo que le hayan regalado nada. Hay muchos Síndrome de Down que han llegado a la universidad. Hay muchos profesores y maestros con Síndrome de Down.
P25: Fíjate, pues yo no lo he visto en la vida. A lo mejor, estoy muy limitada, ¿eh?
E: De hecho, hace poco hubo una polémica sobre una chica con Síndrome de Down, que sacó su carrera, sacó su magisterio y estaba dando clases en una escuela de profesora. Y claro, ya surgió la polémica de los padres que si no estaba capacitada para dar clases y era una profesora excelente.
P25: La verdad, es que lo diferente y lo distinto es necesario.
E: Y, profesoras y profesores universitarios doctores con parálisis cerebral.
P25: Ahí, veo yo otra cosa diferente. Yo conozco a muchos chicos y chicas con parálisis cerebral, hijos de amigos míos, y los veo tan inteligentes...claro, pero con lo del Síndrome de Down, lo que yo tengo es pura ignorancia. Y sigo pensado, que me tira mucho mi experiencia.
E: También,  el Síndrome de Down, varía mucho según el grado.
P25: Claro, es que nuestra chica tiene un grado muy profundo, y ya tiene 24 años y habla muy poquito. Ella está en un estadio básico, muy básico.
