# Supplementary material for: Is pedagogical training an essential requirement for inclusive education? The case of faculty members in the area of Social and Legal Sciences in Spain
Source: PLoS One. 2021 Jul 2;16(7):e0254250. doi: 10.1371/journal.pone.0254250 (PMC8253417; doi:10.1371/journal.pone.0254250)
Supplement: S1 File — (ZIP) [file pone.0254250.s001.zip › 1.2. MOTIVACIONES.rtf]

Documento:		4. Ciencias Sociales y Jurídicas\P1 CCSS Creencias
Peso:	0
Posición:	24 - 25
Código:	1. Creencias\Concepciones discapacidad\1.2. Motivaciones
E: Y a ti qué es lo que te ha llevado a interesarte y trabajar por la inclusión del alumnado con discapacidad.
P1: Pues, sobre todo, la primera vez que yo lo tuve en clase y sobre todo en un examen que me sentí con carencias importantes, una alumna con discapacidad visual, en este caso, yo vi que me faltaban conocimientos y que me faltaban herramientas. Y ahí empezó todo.


Documento:		4. Ciencias Sociales y Jurídicas\P2 CCSS Creencias
Peso:	0
Posición:	28 - 29
Código:	1. Creencias\Concepciones discapacidad\1.2. Motivaciones
E: A ti, por otro lado, P2, qué es lo que te ha llevado a interesarte o trabajar por la inclusión de los estudiantes con discapacidad. 
P2: Pues porque es mi trabajo. Mi trabajo es intentar llegar al máximo número de estudiantes. No hay mayor satisfacción que tú ver cómo consiguen mejorarle a una persona la vida entre cien. Y teniendo en cuenta lo ingrata que es esta profesión y los que somos profesores vocacionales todos los inconvenientes que nos encontramos. Y yo lo digo siempre, prefiero infinitamente una persona que trabaje y estudie o una persona con necesidades especiales que puede ser hasta una persona que cuida a su madre, que es mayor o lo que sea, o una persona que tenga alguna característica física o de otra índole que le pueda limitar, a la cantidad de vagos que tengo aquí que no saben guardar las formas. Eso lo tengo clarísimo. Prefiero tratar con personas de otro tipo. Entonces, siempre me ha interesado porque es que es mi trabajo, y la experiencia me ha demostrado que suelen ser personas con un buen rendimiento.


Documento:		4. Ciencias Sociales y Jurídicas\P3 CSS Creencias
Peso:	0
Posición:	22 - 23
Código:	1. Creencias\Concepciones discapacidad\1.2. Motivaciones
E: Vale. ¿Qué crees que es lo que te ha llevado a ti a interesarte por la inclusión de estos estudiantes?
P3: Yo creo que forma parte de la persona de cada uno. Lo fácil es decir “tengo un alumno con discapacidad y lo intento evadir para que no me suponga ningún problema”. Y por otro lado, está la implicación para ayudar a estas personas. Así que todo depende de la implicación personal, salvo que hay unos protocolos que te diga cómo tienes que actuar.


Documento:		4. Ciencias Sociales y Jurídicas\P4 CCSS Creencias
Peso:	0
Posición:	26 - 27
Código:	1. Creencias\Concepciones discapacidad\1.2. Motivaciones
E: Y qué crees que te ha llevado a ti a interesarte por este tipo de alumnado, si es que hay algo que te ha motivado especialmente.
P4: Pues, el sentido de la justicia, que todo el mundo tiene el derecho a tener las mismas oportunidades, que no porque puedas tener algún tipo de necesidad especial, tengas que estar toda tu vida haciendo el doble de esfuerzo con respecto a otra persona para conseguir lo mismo, ¿no?


Documento:		4. Ciencias Sociales y Jurídicas\P5 CSS Creencias
Peso:	0
Posición:	30 - 31
Código:	1. Creencias\Concepciones discapacidad\1.2. Motivaciones
E: Estupendo. Y qué crees tú que es lo que te ha llevado a interesarte por los estudiantes con discapacidad.
P5: Yo es que creo que eso va en el cargo o en el puesto. Yo es que eso es una cosa que aprendí de un profesor y lo he adoptado desde el principio. Yo trato de memorizar el nombre de mis alumnos y alumnas, yo les llamo por su nombre y cuando veo que alguien, pues viene poco a clase o da mucho por culo en clase y perdón la expresión, o veo que tiene alguna rareza… Yo tenía un alumno que pensaba que me iba a pegar, yo decía “este un día en la clase…”, hablé con él y lo que le pasaba es que se ponía muy nervioso en esta asignatura, la tenía enquistada y le costaba mucho trabajo seguir las clases sin ponerse violento…pero, qué te estaba diciendo.


Documento:		4. Ciencias Sociales y Jurídicas\P5 CSS Creencias
Peso:	0
Posición:	32 - 33
Código:	1. Creencias\Concepciones discapacidad\1.2. Motivaciones
E: Sí, te preguntaba por el tema de qué crees tú que te llevaría en un momento dado a interesarte por la discapacidad y tú me decías que eso va en el cargo.
P5: Eso, que o bien le puede hacer falta una atención distinta o te lo demanda, yo entiendo que es que eso hay que hacerlo, vamos.


Documento:		4. Ciencias Sociales y Jurídicas\P5 CSS Creencias
Peso:	0
Posición:	35 - 36
Código:	1. Creencias\Concepciones discapacidad\1.2. Motivaciones
Yo sí noto, hablando con otros compañeros, lo que te decía antes, no es que sea especialmente sensible, creo, con el alumnado, pero sí me parece que tengo en cuenta al alumno más de lo que lo tienen en cuenta el resto de mis compañeros, tampoco todos, pero yo muchas veces hablando con mis compañeros “los niños de ahora, no sé qué…”. Nos estamos haciendo mayores me imagino y hacemos discursos a veces muy…
E: Eso de cualquier tiempo pasado fue mejor, ¿no?


Documento:		4. Ciencias Sociales y Jurídicas\P6 CCSS Creencias
Peso:	0
Posición:	14 - 15
Código:	1. Creencias\Concepciones discapacidad\1.2. Motivaciones
E: Y qué crees que es lo que te llevaría a ti a interesarte por los alumnos con discapacidad.
P6: Yo es que me gusta interesarme por las personas independientemente de que tengan discapacidad o no la tengan y procuro estar muy cercana a mis alumnos, siempre manteniendo una distancia prudencial entre ellos y yo porque creo que es necesaria para ambas partes, pero me lleva a interesarme el hecho de poder ayudarles en lo que esté en mi mano, ese proceso de aprendizaje que, como hemos dicho antes, puede ser más difícil en esos casos.


Documento:		4. Ciencias Sociales y Jurídicas\P7 CCSS Creencias
Peso:	0
Posición:	89 - 91
Código:	1. Creencias\Concepciones discapacidad\1.2. Motivaciones
P7: Pues yo es que estoy trabajando en temas de servicios y estrategias sociales, de consultoría, hice varios planes estratégicos, de igualdad de oportunidades, de hombre y mujer, para la Junta de Castilla y León, para el Cabildo de Tenerife. Y entonces, de ahí tengo alguna cosa. Luego formación también en cuanto a que la vida misma me ha hecho pasar por…yo pertenezco a una familia numerosa, pues eso también te hace ser más sensible a diferentes personalidades. Luego, mi hermano hizo la tesis, que mi hermano es profesor de música, e hizo la tesis sobre la inclusión de inmigrantes y cómo la música…
E: Como elemento integrador.
P7: Eso. Las diferentes culturas y cómo integrarlo en la música. Ahí también, yo me acuerdo que, hasta que defendió la tesis, estuvimos discutiendo cosas, viendo…le ayudé yo también un poco con metodología y ahí aprendí también sobre el tema de inclusión, que no veía. Y sobre todo la experiencia, el ver casos.


Documento:		4. Ciencias Sociales y Jurídicas\P8 CSS Diseños
Peso:	0
Posición:	10 - 11
Código:	1. Creencias\Concepciones discapacidad\1.2. Motivaciones
E: Bueno, comenzamos por la parte de las creencias acerca de la discapacidad, ¿qué crees que es lo que te ha llevado a interesarte por este tipo de alumnado P8?
P8: Pues yo creo que, a ver, si te soy sincera, yo no me lo plantee como tal, lo que pasa es que una vez que los tenía, pensé que teníamos que dar lo más que tuviéramos a nuestro alcance para ellos. O sea, yo no me lo plantee como un objetivo, me llegaron como alumnos, yo no lo solicité, por decirlo de alguna manera. Y ahora, por qué después actué de una manera que yo creo que es la razonable, podría haber sido mejor seguramente, pero razonable, porque creo que hay darles todas las oportunidades que estén en nuestra mano para que se integren totalmente. 


Documento:		4. Ciencias Sociales y Jurídicas\P10 CCSS Creencias
Peso:	0
Posición:	25 - 27
Código:	1. Creencias\Concepciones discapacidad\1.2. Motivaciones
E: Y, a ti como docente, que te han seleccionado como un profesor que se han sentido incluidos en tu clase, cosas que, en otras no pasa, ¿qué crees que es lo que te ha llevado a tener un cierto interés por estas personas, que otras no lo tienen?
P10: No sé, digamos, que no tengo una…no soy consciente de ninguna cuestión especial. No sé, entiendo que hay gente muy diversa, hay gente que ves que no…que le cuesta mucho, y a lo mejor no está incluido en ningún tipo de…y entonces, pues bueno, “pues oye, ven a tutorías, así no tal…”, pero que no, no tengo conciencia de haber hecho nada especial. Aparte, que me parecería inadecuado dar un trato especial…ni yo me sentiría cómodo, ni, a veces, pienso que se sentiría cómoda la otra persona, que le trates de una forma muy especial. No sé, al menos, yo lo veo así.
E: Ya.


Documento:		4. Ciencias Sociales y Jurídicas\P11 CCSS Creencias
Peso:	0
Posición:	29 - 30
Código:	1. Creencias\Concepciones discapacidad\1.2. Motivaciones
E: Vale, muy bien. Y qué crees que es lo que te ha llevado a interesarte por los estudiantes con diversidad.
P11: Sí, mi amor por la docencia.


Documento:		4. Ciencias Sociales y Jurídicas\P12 CCSS Creencias
Peso:	0
Posición:	28 - 29
Código:	1. Creencias\Concepciones discapacidad\1.2. Motivaciones
E: ¿Qué crees que es lo que te ha llevado a ti a interesarte por los estudiantes con discapacidad?
P12: Yo tengo que decirte que yo no me he interesado por los estudiantes con discapacidad, sino que han venido a mi aula como un estudiante más. La universidad no tiene la política de asignar estudiantes a un profesor, sino que aleatoriamente me ha tocado a mí.


Documento:		4. Ciencias Sociales y Jurídicas\P13 CCSS Creencias
Peso:	0
Posición:	27 - 28
Código:	1. Creencias\Concepciones discapacidad\1.2. Motivaciones
E: ¿Y a ti qué crees que te ha llevado a interesarte por la inclusión de los…?
P13: A ver, a mí me ha venido dado porque son parte del grupo que se te asigna en el POD, o sea, son asignados, tú no los eliges. Estos alumnos no son separados del resto a la hora de la matrícula.


Documento:		4. Ciencias Sociales y Jurídicas\P14 CCSS Creencias
Peso:	0
Posición:	22 - 23
Código:	1. Creencias\Concepciones discapacidad\1.2. Motivaciones
E: Y, claro, esta consideración de la que tú hablas no es la que por desgracia tienen todos los profesores, entonces, me gustaría preguntarte qué es lo que te ha llevado a ti a prestar esa atención a personas que tienen una dificultad añadida.
P14: No sé. Primero, no sé si el resto tienen esa concepción o no, pero al final es reflexionar sobre la experiencia que uno ha tenido. Yo comprendía que cierta persona no podía escribir a cierta velocidad como otra con cinco dedos en la mano derecha, es algo bastante comprensible, creo.


Documento:		4. Ciencias Sociales y Jurídicas\P14 CCSS Creencias
Peso:	0
Posición:	24 - 25
Código:	1. Creencias\Concepciones discapacidad\1.2. Motivaciones
E: Y otro tipo de discapacidades que, a lo mejor pueden ser un poquito…como el tema de la ansiedad o de…no sé, otro tipo de discapacidades que a lo mejor afectan de manera más directa a lo que ocurre en el aula.
P14: Sí. Me parece bastante sorprendente porque parece ser que no es un caso aislado, y hay muchos estudiantes con un problema de ansiedad, y eso me lo comentan, pues que la USE le comunica que hay algunos que tienen problemas de ansiedad y claro, yo me pongo en la piel de cuando era estudiante y también lo pasaba mal, pero no considero que tuviera un problema de ansiedad, y ya lo pasaba mal. Entonces, si eso es un problema incontrolable, pues yo entiendo que tengan ciertas dificultades para superar los cursos. Y, entonces, bueno, por una parte, está bien que alguien tenga que trabajar en preocuparse de cómo hacer que estas personas, ¿no? con problemas de ansiedad, puedan tener las mismas oportunidades que los demás. O facilitarles el control, ¿no? Por ejemplo, dándoles un aula para el examen que permite que puedan pensar con más tranquilidad y calma y no sobresaltarse. Entonces, por la experiencia, por empatía… Intentas ponerte en su lugar. 


Documento:		4. Ciencias Sociales y Jurídicas\P15 CCSS Creencias
Peso:	0
Posición:	30 - 31
Código:	1. Creencias\Concepciones discapacidad\1.2. Motivaciones
E: Y qué crees que es lo que te ha llevado a interesarte por los estudiantes con discapacidad.
P15: Yo no me he interesado, lo que pasa es que dentro de mi trabajo está atender las recomendaciones que me dicen desde aquí de la universidad.


Documento:		4. Ciencias Sociales y Jurídicas\P16 CCSS Creencias
Peso:	0
Posición:	20 - 21
Código:	1. Creencias\Concepciones discapacidad\1.2. Motivaciones
E: ¿Qué crees que es lo que te ha llevado a interesarte por la inclusión, es decir a trabajar por la inclusión en la universidad? 
P16: Pues, yo no tengo ninguna motivación en especial, yo parto del principio de igualdad. A mí la discapacidad no me parece que sea una generadora de una movilización especial de los demás. Sino que hay que darle un tratamiento igualitario. Evidentemente el tratamiento igualitario significa una discriminación positiva. 


Documento:		4. Ciencias Sociales y Jurídicas\P17 CCSS Creencias
Peso:	0
Posición:	44 - 47
Código:	1. Creencias\Concepciones discapacidad\1.2. Motivaciones
E: A mí me da la sensación de que eres una persona que te preocupas por tu alumnado, por estas dos personas que has tenido, por tu experiencia, ¿tú qué crees que te ha llevado a ser así, a hacer eso?
P17: Bueno, yo creo que el ánimo de servir. Yo vengo aquí para divertirme. Yo vengo a la universidad, yo soy profesor asociado nada más, yo no soy profesor titular, vengo a la universidad para divertirme. Tengo mi trabajo en el que gano mi sueldo, y aquí vengo para echar un ratito.
E: ¿Te gusta?
P17: Sí, sí. Vengo porque me gusta dar clase, sino no vendría. Vengo sin echarle cuenta, porque como le eche cuenta seguro que no me sale rentable. Y, vengo para divertirme, entonces, vengo por vocación, me gusta dar clase, y como me gusta dar clase, me gusta que la gente aprenda. Y, en este caso, si tengo un alumno con discapacidad, tengo que estar más pendiente porque quiero que aprendan. En el caso del año pasado tenía alumnos que eran mayores, de edad, de los cuales, bueno, dos, porque el otro se tuvo que dar de baja, los dos que terminaron el curso aprobaron mi asignatura, y yo insistía mucho con ellos, y ellos me lo decían “es que somos muy mayores”, “bueno, no es que seáis mayores, es que lo que tenéis es que comprender lo que se explica, comprenderlo, más que aprenderte la teoría de memoria, es comprender las cosas, porque se os pregunta comprensión, no estudiar de memoria”, y entonces, yo insistía bastante con ellos, mucho. Quizá, por eso, en este caso, Miguel, que coincidía que tenía una minusvalía y que era bastante mayor, pues a lo mejor por eso ha visto que insisto mucho, pero la verdad es que yo insisto mucho con todos los alumnos, y, sobre todo, con los que muestran interés, claro.


Documento:		4. Ciencias Sociales y Jurídicas\P19 CCSS Creencias
Peso:	0
Posición:	24 - 25
Código:	1. Creencias\Concepciones discapacidad\1.2. Motivaciones
E: ¿Qué crees que es lo que te ha llevado a interesarse y a trabajar por la inclusión de los estudiantes con discapacidad?
P19: Pues realmente, nada, simplemente me encanta la docencia, y si veo que en mi clase está Alberto, pues tengo que trabajar con él y llegar a él igual que a los demás. No, no hay nada especial que me lleve a…en mi vida personal no tengo ningún pariente con discapacidad ni nada. La cuestión es llegar a todos.


Documento:		4. Ciencias Sociales y Jurídicas\P20 CCSS Creencias
Peso:	0
Posición:	28 - 29
Código:	1. Creencias\Concepciones discapacidad\1.2. Motivaciones
E: Y qué crees que es lo que a ti te ha llevado a inP20rte por estos alumnos, si es que señalarías algo que crees que te ha motivado a tener esa mayor sensibilidad.
P20: Pues mira, yo soy sorda de un oído. Nunca me he preocupado de si tengo algún tipo de…pero siempre me he preocupado más de esos temas porque creo que te puede entorpecer el día a día en tu labor, no sé, si tienes una discapacidad auditiva, visual o demás, pero que no te tiene que entorpecer para el resto de las cosas. Entonces, yo creo que como no te tiene que entorpecer en el resto de las cosas, hay que poner medios para lograr su inclusión en todos los aspectos.


Documento:		4. Ciencias Sociales y Jurídicas\P21 CCSS Creencias
Peso:	0
Posición:	42 - 43
Código:	1. Creencias\Concepciones discapacidad\1.2. Motivaciones
E: Lamentables casi, se diría. Y, ¿qué cree que es lo que le ha llevado a interesarse por los estudiantes con discapacidad?
P21: Vuelvo a partir siempre desde el principio de que mi función es atender al estudiante. Entonces, quizá aquí, lo que sí es evidente es, a primera vista, el hecho de que ese estudiante sí va a necesitar de ti de una manera especial, ¿no? Aunque sigo pensando que hay otros alumnos sin discapacidad a los que podría haber ayudado pero que están ahí perdidos en esa masa y al final no…como digo, no es un problema de discapacidad, es, simplemente, unas circunstancias especiales, que es lo que hablábamos, alumnos que no…yo llegué a tener un alumno que, cuando vino en septiembre a la revisión y tal, y hablo ya de septiembre porque en junio suspendió, y me enteré en septiembre de las circunstancias. Y era un alumno que había tenido una enfermedad a lo largo del cuatrimestre, que es verdad que yo lo veía al principio asistir con regularidad a clase y después dejó de asistir, pero eso ocurre a menudo, alumnos que empiezan constantes a la asignatura y, por las circunstancias que sea, dejan de venir a clase y tal, que, como yo le dije, “si hubieras hablado conmigo en un primer momento, yo hubiera atendido tus circunstancias”, ya sean circunstancias…que lo tuvieron que operar, que estuvo un mes y pico entre ingresos y rehabilitación y tal, y eso hizo completamente perder el ritmo y le dije “si me lo hubieras dicho, hubiéramos hecho algo”. Unos años atrás tuve un alumno que sí me lo planteo desde el primer momento, que lo iban a operar y digo, pues mira, ya vamos viendo por correo electrónico y tal. Y, desde el hospital, fue siguiendo la asignatura, y no le supuso a tener que renunciar en un momento determinado a examinarse en junio, ¿no? Entonces, por eso digo, que simplemente es atender las necesidades y las demandas. Pero, insisto, como un alumno más, yo siempre parto desde el principio de que me parece que hay que militar de no ver a la persona como…de entender la discapacidad como inferioridad, ¿vale? Porque esa actitud paternalista, esa actitud de pobrecito…no, no se le hace ningún favor. Tiene una, entre comillas, discapacidad, que lo hemos calificado así, pero insisto, otros tenemos otro tipo de discapacidades, no sé si, realmente lo analizamos. No es una cuestión de favor, es como cuando tú dices, es que es muy grande la discapacidad, qué putada, vale sí, es una putada, pero y qué, lo único que se trata es de que, atendiendo tus circunstancias, pues tirar para adelante, ¿no? Y el nivel de exigencia es el mismo en cuanto a resultados, que, en el proceso, insisto, no te voy a decir pobrecito y no te voy a aprobar si haces una mamarrachada de trabajo. Ahora, si necesitas más tiempo para realizar el trabajo o me lo tienes que entregar en estas circunstancias o me lo tienes que dar en este formato, o que te tengo que hacer el examen en un momento determinado y oral… Pues sí, pues todo eso se hace, ¿no? Pero partiendo de que tú te estás esforzando y de que yo voy a valorar tu esfuerzo y tus resultados, ¿no? Pero como con cualquier otro alumno, insisto, que yo puntúo el esfuerzo de los alumnos. Hay alumnos que…digo con esto que la evaluación tampoco es ir más con el sistema de examen tan horroroso que tenemos, esto no es lo que me has puesto en el examen, porque es verdad, que la universidad no facilita, digamos, evaluación continua, sobre todo, con las masas de estudiantes, pero con algunos si lo podemos hacer. Entonces, yo tengo alumnos que no tienen ningún tipo de discapacidad, pero que han ido mostrando un esfuerzo a lo largo del cuatrimestre y, el día del examen, por las circunstancias que sea, como yo digo, yo recuerdo un examen que hice en mis tiempos de estudiante, con un dolor de muelas de la noche anterior, harto de calmantes, y me presenté al examen y me salió bastante regular, con lo cual, entiendo perfectamente que el examen puede salir…o, al contrario, salirte estupendamente porque tienes detrás al que te sopla mejor que nadie. Con lo cual, yo hay alumnos que he visto que en un momento determinado en el examen es muy flojo, y me ha llamado la atención que sea tan flojo en vista del esfuerzo que yo he visto que han hecho a lo largo del cuatrimestre, y, no es que les esté haciendo un favor, es que lo que estoy es poniendo ese esfuerzo sobre los resultados del examen, y, lo evalúo por encima de lo que realmente está teniendo el papel. Y, en este caso, con discapacidad o no, se trata de hacerlo así, ¿no?


Documento:		4. Ciencias Sociales y Jurídicas\P21 CCSS Creencias
Peso:	0
Posición:	143 - 143
Código:	1. Creencias\Concepciones discapacidad\1.2. Motivaciones
P21: Claro, y es que no sabría decirte qué sé en el sentido de...hombre, es un tema que me interesa y sé lo que puede saber cualquier persona. Que me gusta pensar que soy una persona informada, ¿no? Y me interesa el tema. Es un tema al que presto atención como en líneas generales a…yo siempre digo, por hablar de las secciones de un periódico, ¿no?  Yo nunca leo las páginas de deporte, pero siempre leo las páginas de sociedad. No de sociedad rosa, sino de los temas... Porque me interesan los temas sociales y ahí suele estar incluido...


Documento:		4. Ciencias Sociales y Jurídicas\P22 CCSS Creencias
Peso:	0
Posición:	24 - 25
Código:	1. Creencias\Concepciones discapacidad\1.2. Motivaciones
E: Y, ¿qué cree que es lo que le ha llevado a interesarse por el alumnado con discapacidad?
P22: Que son muy sensibles, que veo que les cuesta integrarse en la clase, y eso, la verdad es que me pasa…me da mucha pena, ¿no? Entonces, siempre intento preguntarles, ponerle el micro delante… Este curso, por ejemplo, he tenido a un chaval en silla de ruedas, entonces, le pregunté lo mismo “¿tú tienes grupo?”, y lo integré con un grupo, pero, al final ese grupo me dijo que si no había trabajado, que si tal y que si cual, y no quisieron incluirle su nombre en el trabajo. El chaval vino a hablar conmigo, me dijo que se le había estropeado el ordenador, que necesitaba un tipo de programa específico…bueno, en fin, sus diferentes cosas y tal, pero se ha tenido que quedar para septiembre, porque yo hasta ese punto tampoco puedo ayudarle. 


Documento:		4. Ciencias Sociales y Jurídicas\P23 CCSS Creencias
Peso:	0
Posición:	32 - 35
Código:	1. Creencias\Concepciones discapacidad\1.2. Motivaciones
E: Estupendo. Y, ¿qué cree que es lo que le ha llevado a interesarse por estudiantes con discapacidad?
P23: Te confieso, Almudena, que yo no me he interesado por estudiantes con discapacidad, no he buscado nada que... Simplemente que me ha tocado en algunas asignaturas. No sé si los alumnos me han elegido, porque tú sabes que ellos ven qué profesor da cada grupo y ellos eligen a qué grupo irse. No lo sé. Tampoco se lo pregunté a los alumnos que he tenido. Pero a mí ha sido como algo que me ha tocado y que tiraba para adelanta con eso.
E: Bueno, quizá le ha tocado por algo positivo, seguramente.
P23: No, ni positivo, ni negativo, que me ha tocado simplemente.


Documento:		4. Ciencias Sociales y Jurídicas\P24 CCSS Creencias
Peso:	0
Posición:	19 - 19
Código:	1. Creencias\Concepciones discapacidad\1.2. Motivaciones
Entonces, me viene, no sé si en mis genes o…yo recuerdo que cuando era pequeña e iba por mi pueblo, en Constantina, como te he dicho, y es muy propio en Andalucía ir con los pasos y mi madre me llevaba y vimos a una niña detrás de una ventana en una casa. Tú sabes que hay unos ventanales grandes a pie de calle y le decía “mamá y esos niños, ¿por qué no están aquí con nosotros?”, “no pueden estar porque están malitos”, decía mi madre. Claro, te hablo de aquella época, de hace ya más de 40 años y a mí eso me marcó. Creo que fue el detonante, ¿no? Y después, en mi pueblo he dado clases cuando era pequeña a personas en riesgo de exclusión social. Y posteriormente, siempre que puedo, colaboro, en este caso con la asociación de discapacidad intelectual, ¿no? Después, a nivel familiar, mi tío tenía una amputación de un brazo, que eso también…no sé, te digo conexiones que puede haber con estas cuestiones. 


Documento:		4. Ciencias Sociales y Jurídicas\P24 CCSS Creencias
Peso:	0
Posición:	19 - 19
Código:	1. Creencias\Concepciones discapacidad\1.2. Motivaciones
Y claro, mi formación me ha llevado a que cuando he llegado a la universidad, pues a que tenga otra mirada. No ha sido como a lo mejor la persona que no lo tiene. Pero te vuelvo a repetir, a lo mejor no tiene la formación, pero tiene ese familiar, que es una formación informal, ¿no? Y hay otros que, por carrera, por ideología, por religión… Por muchos factores, están bloqueados. Y yo te aseguro que el mayor desconocimiento es la ignorancia y hay mucha ignorancia en este tema y miedo, miedo al qué dirán. Pero cuando conocen realmente el potencial y las capacidades, se implican muchísimo el profesorado.


Documento:		4. Ciencias Sociales y Jurídicas\P25 CCSS Creencias
Peso:	0
Posición:	42 - 43
Código:	1. Creencias\Concepciones discapacidad\1.2. Motivaciones
E: Muy bien. Y, ¿crees que haya algo que te haya hecho a ti interesarte de alguna manera por los estudiantes con discapacidad?
P25: Sí, yo creo que sí, que a mí me lleva el tema de la vulnerabilidad. Yo creo que es propio del trabajo social. Yo creo que es un poco la sensibilidad hacia las personas con desventajas. 
