# Supplementary material for: Is pedagogical training an essential requirement for inclusive education? The case of faculty members in the area of Social and Legal Sciences in Spain
Source: PLoS One. 2021 Jul 2;16(7):e0254250. doi: 10.1371/journal.pone.0254250 (PMC8253417; doi:10.1371/journal.pone.0254250)
Supplement: S1 File — (ZIP) [file pone.0254250.s001.zip › 1.3. CARACTER╓STICAS (1).rtf]

Documento:		4. Ciencias Sociales y Jurídicas\P1 CCSS Creencias
Peso:	0
Posición:	26 - 27
Código:	1. Creencias\Rol docente y actitudes\1.3. Características
E: Vale. Y en relación con tu papel y rol como docente y actitudes, ¿podrías decirme algunas características que te definen a ti como docente?
P1: Responsable…no sé, se me vienen a la cabeza muchas cosas, pero quizá no sea yo el que lo tendría que decir, sino el alumno en cuestión. No sé, me preocupo por los alumnos, pero con discapacidad o sin discapacidad.


Documento:		4. Ciencias Sociales y Jurídicas\P2 CCSS Creencias
Peso:	0
Posición:	30 - 31
Código:	1. Creencias\Rol docente y actitudes\1.3. Características
E: En cuanto a ti como docente P2, dime brevemente las principales características que te definirían como profesor.
P2: Pues yo procuro, aparte de que soy profesor vocacional y eso, pues yo procuro hacer las cosas lo mejor posible, procuro implicarme, hasta el punto que me tengo que implicar, estar totalmente actualizado, y puedo decir que en los 20 años que llevo dando clase, siempre he impartido el temario en su totalidad, nunca he faltado a una clase y nunca he dejado de tratar a un alumno en tutoría. He cumplido mi trabajo. 


Documento:		4. Ciencias Sociales y Jurídicas\P3 CSS Creencias
Peso:	0
Posición:	24 - 25
Código:	1. Creencias\Rol docente y actitudes\1.3. Características
E: Vale. Refiriéndome a tu rol como docente, ¿qué características te definen a ti como profesor?
P3: Yo creo que la implicación hacia los alumnos. Mi característica es el nivel de orientación que siempre he tenido hacia los alumnos, hacia los estudiantes. Nunca los he considerado como personas que pasan por aquí y ya está, sino que he tenido una relación cercana y buena con ellos y yo creo que en eso se ponme de manifiesto mi preocupación por las personas tanto con discapacidad como sin discapacidad. 


Documento:		4. Ciencias Sociales y Jurídicas\P4 CCSS Creencias
Peso:	0
Posición:	28 - 29
Código:	1. Creencias\Rol docente y actitudes\1.3. Características
E: Y ahora, con respecto a tus actitudes y tu rol docente, ¿podrías decirnos algunas características que creas que te definen como docente?
P4: Bueno, pues soy bastante empática e intento centrarme tanto en el contenido, como en la materia, como en la forma de transmitirla, preocuparme mucho de la forma de transmitirla a los estudiantes. Te estoy hablando desde el punto de vista general, no desde la discapacidad.


Documento:		4. Ciencias Sociales y Jurídicas\P5 CSS Creencias
Peso:	0
Posición:	34 - 37
Código:	1. Creencias\Rol docente y actitudes\1.3. Características
E: Te voy a preguntar ahora por tu papel y tu actitud hacia la docencia, ¿podrías decirme algunas características que te definan como docente?
P5: Puf, yo qué sé. Yo soy honesto con mis clases, o intento ser honesto con mis clases, yo me las preparo lo mejor que puedo y eso no significa que…tú lo sabes, además, como profesor, hay clases que te preparas y salen muy mal, muy mal. Yo he salido un montón de veces pensando “por qué haces esto, deja de dar clase”, pero yo trato de prepararme mis clases. Yo sí noto, hablando con otros compañeros, lo que te decía antes, no es que sea especialmente sensible, creo, con el alumnado, pero sí me parece que tengo en cuenta al alumno más de lo que lo tienen en cuenta el resto de mis compañeros, tampoco todos, pero yo muchas veces hablando con mis compañeros “los niños de ahora, no sé qué…”. Nos estamos haciendo mayores me imagino y hacemos discursos a veces muy…
E: Eso de cualquier tiempo pasado fue mejor, ¿no?
P5: Eso no significa que…que yo a veces también participo un poco de eso, ¿no? Porque es verdad que hay algunos alumnos, depende también de la disciplina, porque eso también es verdad, depende de dónde viene cada uno, cómo se comporta en clase y tal, ¿no? Pero yo trato de ser cercano con los alumnos, ¿no? En ese equilibrio que ti también sabrás que hay que mantener entre “no soy tu profesor que está en el altar, pero tampoco soy tu colega”, ¿no? Porque es que esa línea es muy delgada, de cómo te acercas tú, pero manteniendo la posición de profesor-alumno. Pero bueno, yo no me meto tanto en esos berenjenalitos, doy un pasito atrás creo, a lo que hacen otros compañeros míos, ¿no?


Documento:		4. Ciencias Sociales y Jurídicas\P6 CCSS Creencias
Peso:	0
Posición:	16 - 19
Código:	1. Creencias\Rol docente y actitudes\1.3. Características
E: Estupendo. Y ahora te voy a hacer unas preguntas sobre tu perfil como docente ¿Me podrías decir algunas características que pienses que te definan a ti como docente?
P6: Es complicado.
E: Sí, siempre pienso que es una de las preguntas más complicadas de la entrevista.
P6: Yo me considero muy responsable con mi trabajo, entonces, para mí no es llegar a la clase y soltar la parte de la asignatura que me corresponda e irme a casa y que no haya habido un intercambio con el alumnado. Entonces, para mí es muy importante, por ejemplo, conocer sus nombres, dirigirme a ellos directamente, que ellos tengan totalmente libertad para dirigirse a mí cuando quieran… Entonces, creo que el proceso de aprendizaje es un proceso de doble dirección, ¿no?, que no es que tú llegas y yo te cuento la parte de la asignatura y nos vamos, sino que yo creo que, procuro hacer mis clases lo más amenas posibles, doy clases de economía que es mi especialidad y, por tanto, también procuro que siempre encuentren ejemplos en la realidad para poder aplicar los conceptos que estamos haciendo en clase, que creo que es importantísimo llevarlos para que lo entiendan y para que lo vivan en su día a día. Y, ya está, no creo que haga mucho más aparte de eso.


Documento:		4. Ciencias Sociales y Jurídicas\P7 CCSS Creencias
Peso:	0
Posición:	30 - 31
Código:	1. Creencias\Rol docente y actitudes\1.3. Características
E: ¿Cómo podrías definirte o cuáles son algunas de las características que te definen como docente en general? No solamente como docente de alumnado con discapacidad. 
P7: Pues yo creo que sobre todo es que me gusta, que lo he elegido yo la docencia, lo he elegido muy mayor ya y me gusta. Y para mí no es un trabajo, sino que es un disfrute y veo personas y yo creo que se le puede marcar mucho a un chico a esta edad. Se le puede marcar mucho para bien o para mal, y tengo mucha conciencia de que he tenido profesores muy malos, también muy buenos, y eso me ha marcado mucho de que quería ser profesor e intentar cambiar. No estoy aquí por dinero, sino que estoy porque me gusta mi profesión y, sobre todo, que veo un trato humano. Y ese es el difícil equilibrio que intento buscar en clase. Tienes que buscar una distancia como profesor, pero también no puedes mostrarte nunca…y eso es lo que hace que los alumnos te vean como una persona accesible, humana, aun cuando no te pones al mismo nivel de ellos, pues yo creo que es algo que también les hace acudir a ti más que a otro profesor porque les crea una barrera.


Documento:		4. Ciencias Sociales y Jurídicas\P8 CCSS Creencias
Peso:	0
Posición:	26 - 29
Código:	1. Creencias\Rol docente y actitudes\1.3. Características
E: P8, entonces tú, ¿qué características crees que te definen como docente?
P8: Yo soy absolutamente vocacional, no te lo puedes ni imaginar. En contra, soy bastante crítica con los sistemas actuales porque a mí no me da la vida. Si le dedico a los alumnos el tiempo que les dedico, no me da la vida para más y eso que bueno, pues si hablas con mis alumnos te pueden decir que soy capaz de contestarles un correo a las tres de la mañana, cosas de ese tipo porque me pongo a avanzar cosas que no he podido avanzar aquí y bueno. Tengo que hacer el cuadrante de no sé qué, o terminar de escribir un informe o no sé qué y de repente me llega un correo de “que tengo una duda, que me he atascado” pues a ver, la duda, le puedo contestar a las…
E: Me suena, me suena a mí eso.
P8: Claro, es muy vocacional.


Documento:		4. Ciencias Sociales y Jurídicas\P8 CCSS Creencias
Peso:	0
Posición:	66 - 67
Código:	1. Creencias\Rol docente y actitudes\1.3. Características
E: P8, ¿qué actitudes personales crees tú que ayudaron? Tuyas, personales tuyas.
P8: Sí. Pues yo creo que una actitud de cercanía. Yo creo que eso le ayudó, o sea, me sentía como una persona dispuesta a apoyarle en lo que hiciera falta siempre y cuando tuviese como compensación ver que respondía positivamente. Eso también se lo dije “mira, si yo estoy aquí dejándome la piel para que las cosas te vayan sobre ruedas, que no tengas ningún problema con la asignatura, si empiezas en plan pasota, paso yo más que tú, que yo pasar también sé”. Y entonces, yo creo que se creó esa especie de magia de “tú estás apostando por mí y no voy a defraudarte y aquí estoy al cien por cien y vamos a terminar teniendo una relación más allá casi de profesor-alumno, más casi de amistad”.


Documento:		4. Ciencias Sociales y Jurídicas\P8 CSS Diseños
Peso:	0
Posición:	12 - 13
Código:	1. Creencias\Rol docente y actitudes\1.3. Características
E: Y con respecto al rol docente y las actitudes, cómo crees que influyen las características que tú tienes como docente en el aprendizaje del alumnado.
P8: Yo creo que muy positiva, porque estoy a su total disposición. Soy una profesora a la antigua usanza, donde la docencia es para mí muy importante. No solo la parte investigadora, que es la que se valora de una manera más prioritaria.


Documento:		4. Ciencias Sociales y Jurídicas\P9 CCSS Creencias
Peso:	0
Posición:	30 - 31
Código:	1. Creencias\Rol docente y actitudes\1.3. Características
E: ¿Puedes decirme algunas características que te definen como docente?
P9: Pues no sé, yo creo que intento ponerme mucho en el lugar de ellos, o sea, el entender que, aunque para mí hay cosas que puedan ser muy sencillas porque tengo una determinada experiencia, para ellos hay cosas que no son tan fáciles de ver. Entonces, ya no solamente por eso, sino porque es verdad que todos les mandamos muchas cosas, que ellos no tienen tanto tiempo como nos creemos, que son jóvenes y tienen que vivir además de estudiar… Entonces, esa parte, yo creo que intento un poco que ellos puedan hacer en clase todo lo máximo, que se les quede poco para hacer después. Van a tener que estudiar, que hacer un trabajo, porque todo no lo van a poder hacer en clase. Pero yo creo que eso es una parte, y el hecho de que ellos piensen y se den cuenta de que yo estoy disponible para ellos cuando ellos lo necesiten. Ellos saben que yo soy asociada y no estoy en el despacho, pero ellos saben que si me escriben tardo poquísimo o intento tardar poco en contestarles e intento dar respuesta a todo lo que me pregunten. O sea, intento estar lo más cerca de ellos porque creo que es la única forma en la que realmente les puedo ayudar a que aprendan y a que aprendan no sé, no solamente los contenidos, que al fin pues es el objetivo, sino que, no sé, que vean que el mundo laboral es complicado pero que al final son todos personas. Las empresas son personas, la sociedad son personas. Entonces, yo creo que es lo que trato, el ser lo más próximo a ellos.


Documento:		4. Ciencias Sociales y Jurídicas\P10 CCSS Creencias
Peso:	0
Posición:	29 - 32
Código:	1. Creencias\Rol docente y actitudes\1.3. Características
E: Y, si hablamos un poco de tu rol docente, ¿vale? Dentro del aula, sé que sería complicado, pero, ¿podrías definirme o identificar algunas características tuyas que tú creas que tienes?
P10: Pues, no lo sé, no sabría decirte, nunca me he observado, la verdad. Así como dicen que hay gente que cuando habla se escucha, yo tengo ese problema de que no suelo escucharme, entonces…
E: De todo lo contrario.
P10: No sé qué decirte. Hombre, la docencia siempre me ha parecido algo importante., Me dedico a la investigación muchísimo, no tengo malos resultados en el ámbito investigador, y, la docencia, pues, es otra cosa, …pero entiendo, que me lo tomo muy en serio y procuro preparar material. Por ejemplo, la mayoría de las asignaturas en las que doy clase, el material que gasta todo el mundo lo he hecho yo… No sabría decirte nada especial.


Documento:		4. Ciencias Sociales y Jurídicas\P11 CCSS Creencias
Peso:	0
Posición:	31 - 32
Código:	1. Creencias\Rol docente y actitudes\1.3. Características
E: Y con respecto al rol docente y las actitudes, ¿podrías decirme algunas características que creas que te definen como docente?
P11: Sí, a ver, yo me considero un profesor exigente, les trato de trasladar a los alumnos una actitud profesional, y mis objetivos principalmente es que, si cuando empiezo el curso los alumnos tienen un nivel personal y profesional X, que cuando haya acabado la clase, hayan crecido tanto personal como profesionalmente. La actitud, autoestima, disciplina, el amor por el trabajo bien hecho… Es decir, poner retos continuamente y que al final ellos se sientan orgullosos de lo que han conseguido. Entonces, claro, para eso hay que ser muy exigente. Yo tengo fama de ser…de hecho, cuando vienen las evaluaciones, todos los años que nos evalúan, yo les pido que me pongan comentarios y siempre me dicen “este profesor piensa que solo está su asignatura”, porque les hago trabajar mucho. A cambio les pongo buenas notas y la gran mayoría valoran mi pasión por dar la asignatura y mi cercanía, es decir, hay profesores que no les gustan los alumnos, yo todo lo contrario, me gusta relacionarme, los llamo a mi despacho… A mí me rejuvenece, me gusta el trato personal, esa es otra de mis características.


Documento:		4. Ciencias Sociales y Jurídicas\P11 CCSS Creencias
Peso:	0
Posición:	34 - 34
Código:	1. Creencias\Rol docente y actitudes\1.3. Características
P11: Y el elemento motivador, esa es otra característica que tengo, yo soy un motivante nato. Entonces…


Documento:		4. Ciencias Sociales y Jurídicas\P12 CCSS Creencias
Peso:	0
Posición:	30 - 33
Código:	1. Creencias\Rol docente y actitudes\1.3. Características
E: ¿Podrías decirme algunas características que te definan como docente? 
P12: Vamos a ver, yo estoy en un área de empresa, entonces la única característica diferencial que tengo del resto de docentes, es que yo vengo del ámbito profesional. Aunque digamos llevo 25 años de experiencia docente, pero mis orígenes son profesionales. Yo no solo he trabajado en la facultad, así que ésta puede ser la única característica diferencial que puedo tener con el resto de compañeros.
E: Vale, entonces unas de las características que te definen es el poder trabajar en otro ámbito, ¿no?
P12: Exacto y creo que eso aporta un valor añadido a la docencia que impartes. Pero vamos, también ha sido una circunstancia. Yo creo que es un mérito, pero que ha sido una circunstancia.


Documento:		4. Ciencias Sociales y Jurídicas\P13 CCSS Creencias
Peso:	0
Posición:	20 - 20
Código:	1. Creencias\Rol docente y actitudes\1.3. Características
En general me considero una persona muy empática y cuando veo a los estudiantes, enseguida contacto con ellos y sus circunstancias, y si tienen necesidades educativas especiales, pues entiendes rápidamente que deben contar con apoyo, pero acotado a la necesidad especial.


Documento:		4. Ciencias Sociales y Jurídicas\P13 CCSS Creencias
Peso:	0
Posición:	29 - 30
Código:	1. Creencias\Rol docente y actitudes\1.3. Características
E: Y a ti como docente, ¿podrías decirme algunas características que te definan?
P13: Vale. Soy muy accesible, eso los alumnos me lo comentan. Tengo mucha empatía, es vocacional la docencia, y me gusta mucho eso, el aula y la relación con los estudiantes. Yo digo que soy una especie de vampiro, que necesito la energía de los estudiantes para seguir adelante.


Documento:		4. Ciencias Sociales y Jurídicas\P14 CCSS Creencias
Peso:	0
Posición:	26 - 27
Código:	1. Creencias\Rol docente y actitudes\1.3. Características
E: Vale. Y, si hablamos ahora un poco sobre tu rol docente y tus actitudes como docente, ¿podrías describirme algunas de las características que crees que te definen como docente?
P14: Pues, accesible, en primer lugar, es decir, siempre invito a que vengan a tutorías si no entienden algo, intento ser cercano porque todavía me siento muy cerca de su posición y cuando explico algo, intento que me confirmen ellos si les ha llegado bien el mensaje, y si no, pues les invito a darle más vueltas… Yo creo que eso, accesibilidad y cercanía.


Documento:		4. Ciencias Sociales y Jurídicas\P15 CCSS Creencias
Peso:	0
Posición:	32 - 35
Código:	1. Creencias\Rol docente y actitudes\1.3. Características
E: Claro. Y con respecto a tu rol docente y actitudes, ¿podrías decirme algunas características que creas que te definen como docente?
P15: Ay, no sé qué contestar a eso.
E: ¿No se te ocurre ninguna? Alguna característica que creas que tengas…
P15: No sé, intento hacer las cosas bien y ya está.


Documento:		4. Ciencias Sociales y Jurídicas\P16 CCSS Creencias
Peso:	0
Posición:	22 - 23
Código:	1. Creencias\Rol docente y actitudes\1.3. Características
E: Con respecto tu papel como docente, ¿qué característica te definen?
P16: Quien tiene que decir esto es el público.


Documento:		4. Ciencias Sociales y Jurídicas\P17 CCSS Creencias
Peso:	0
Posición:	48 - 51
Código:	1. Creencias\Rol docente y actitudes\1.3. Características
E: Y, ¿nos podrías decir tres…bueno, tres no, algunas características que te definan a ti como profesor, como docente?
P17: Como docente…bueno, pues una característica es que a mí me gusta hacerlo sencillo, no pretendo como hacían otros catedráticos conmigo, yo no doy clases de matemáticas, yo doy clases de economía. Entonces, no me voy a centrar tanto en todas las teorías matemáticas aplicadas a la economía, eso tendrán que verlo en matemáticas. Yo quiero que los alumnos entiendan las cosas, quiero que tengan capacidad crítica, y me gusta que lean, me gusta, los impulso a leer, a ver películas, “pues mira, he visto una película que me gustaría que vierais”, les pongo…utilizo mucho los medios visuales, les pongo trocitos de películas, les pongo presentaciones…
E: ¿Pero relacionado con la asignatura?
P17: Sí, sí, sí. Todo relacionado con la asignatura. Si pones la teoría de juegos, qué menos que explicar lo que era una mente maravillosa, pues cuando vean a Nash que, realmente fue el padre de la teoría de juegos, y cómo presenta la teoría de juegos, eso es más explícito y más fácil de enseñarlo así que dar una clase y explicar otra vez el dilema del prisionero, pues yo le pongo a Nash, cuando veía a la chica y decía con cuál iba a ligar y con cuál no, y decía “si colaboramos todos, ligamos, si no colaboramos, no liga ninguna”, y se les queda. Entonces, a mí me gusta que entiendan los conceptos de economía, porque luego los tienen que aplicar, luego, en la empresa, no se aplica de memoria nada, no sirve de nada de memoria, sirve tener los conceptos cuando te enfrentas a algo. No sirve para nada, incluso a los de derecho, les podría decir que aprenderse los códigos de memoria es para nada, eso es terrible. Entonces, yo soy muy práctico, voy a las cosas prácticas, voy a las cosas sencillas, y bueno, hablo demasiado, hablo mucho, cuento muchas cosas, les insisto mucho en que tienen que pensar mal, yo pienso mal y acierto, y les insisto mucho en que tienen que tener…tienen que ser críticos y pensar que el que está enfrente, normalmente, no suele ser generoso, suele ser bastante egoísta, y tiene sus objetivos y tiene sus cosas que cumplir, tiene que enfrentarse a sus cosas. Se lo digo cuando hablen en un banco, con abogados, con economistas, cuando hablen con políticos, mucho más. Y bueno, esa es mi forma de dar clase, que aprendan ideas, que aprendan ideas y que sepan después implementarlas.


Documento:		4. Ciencias Sociales y Jurídicas\P17 CCSS Creencias
Peso:	0
Posición:	75 - 75
Código:	1. Creencias\Rol docente y actitudes\1.3. Características
P17: Sí. Yo creo que sí influye. Yo soy activo. Yo, además, trabajo en Endesa, en un área muy rara que se llama Regulación, y nosotros nos encargamos de batallar con la administración, principalmente y después con algunos clientes que tenemos, que, bueno, no porque sean clientes, sino porque son casos muy específicos de gente que les gusta luchar, mucho.


Documento:		4. Ciencias Sociales y Jurídicas\P17 CCSS Diseños
Peso:	0
Posición:	83 - 87
Código:	1. Creencias\Rol docente y actitudes\1.3. Características
P17: Sí, el cómo los trates influye muchísimo en el aprendizaje.
E: ¿Tú cómo te definirías?
P17: Yo con cercanía.
E: Te consideras una persona cercana, ¿no?
P17: Cercanía. De vez en cuando me estudio alguna ficha, las tengo aquí, y voy viendo quienes son y ese día, pues le toca al que me haya estudiado la ficha, y les sorprende mucho, porque cuando tú les digas “Menganito”, eso les sorprende mucho, porque dicen “ostras, que sabe mi nombre”, entonces, en cada clase me tocan tres o cuatro alumnos para preguntarles directamente.


Documento:		4. Ciencias Sociales y Jurídicas\P18 CCSS Creencias
Peso:	0
Posición:	38 - 41
Código:	1. Creencias\Rol docente y actitudes\1.3. Características
E: Vamos a pasar a tu rol docente, ¿vale?, ¿me podrías decir algunas características que, desde tu punto de vista, te definan como docente?
P18: Es que no sé en qué sentido, ¿que me defina en cómo doy las clases?
E: Cómo tú eres como docente…pues, “me considero una persona muy seria en mi trabajo”, no sé, alguna característica que te defina.
P18: Sí. Hombre, yo soy, en principio, intento cumplir un programa, intento cumplirlo. Sí es cierto que soy seria, pero no totalmente, porque bueno, de vez en cuando, pongo algún ejemplo que esté más relacionado con la realidad, que estamos en materia de empleo ahora, algún tipo de cuestiones que pueden estar más cercanas a la realidad y, entonces, hago algún tipo de comentario. Yo creo que mi relación con los estudiantes, que si ellos me dicen “¿P18 puedo salir un momento ahora?”, que ellos me lo dicen antes de clase y sin ningún problema, pueden salir, entonces, mi relación con ellos…hombre, sí soy seria en el trabajo y soy exigente, soy exigente y exijo que el que esté en clase esté porque quiera estar, no porque simplemente le obligue a pasar una lista, que, de vez en cuando, obviamente paso lista porque tengo también que saberme el nombre, sobre todo, de los alumnos, pero, realmente sí, soy una persona seria, pero tampoco muy seria. Doy pie a que ellos den sus opiniones, porque obviamente si yo me mantengo en mi posición, a lo mejor ellos no dan su opinión personal o fundamentada en derecho, pero una opinión sobre una cuestión en concreto. Y, después, en el tema práctico, yo intento explicar en clase de básica o en la misma clase de la práctica cómo quiero que se haga el supuesto práctico, que se hace en clase, y lo hago para ver si realmente han captado el concepto, si saben afrontar el caso práctico y si se han enterado de lo que he explicado en clase. Es que yo también he dado muchas horas de clase muchos años y no he tenido mucho problema.


Documento:		4. Ciencias Sociales y Jurídicas\P18 CCSS Creencias
Peso:	0
Posición:	44 - 47
Código:	1. Creencias\Rol docente y actitudes\1.3. Características
E: Y cómo crees que tus características, tanto personales como profesionales, influyen en el aprendizaje de tu alumnado.
P18: Hombre, yo creo que, aunque yo sea una persona seria, también son una persona cercana, y yo creo que ellos no tienen miedo de exponer sus opiniones porque yo, además, no soy de las personas que les digan que es un error, que qué está usted diciendo… No, simplemente, les digo “bueno, esto es así, pero, ¿estás seguro?”, entonces, intento que razonen esa respuesta que me ha dado, entonces, yo creo que soy seria, pero también un poco cercana y esa cercanía les permite a ellos, incluso si dan una respuesta negativa, contraria o que no sea la respuesta que yo espero, al menos, que sí puedan reflexionar un poquito, pero es quizá eso, porque soy…aunque sea una persona, ya te digo, seria, pero también cercana. Ellos pueden venir a las tutorías a la hora que quieran, me mandan un correo “mira P18”, y es una tutoría que no esté, pues intento darte una hora de tutorías o buscarte un horario asequible. Yo creo que eso es lo que me define. Tampoco soy la profesora de “yo estoy aquí y tú estás ahí”, no, es una relación…
E: Un poco horizontal.
P18: Sí.


Documento:		4. Ciencias Sociales y Jurídicas\P19 CCSS Creencias
Peso:	0
Posición:	26 - 27
Código:	1. Creencias\Rol docente y actitudes\1.3. Características
E: ¿Podrías decirme algunas características que te definan como docente?
P19: Pues, creo que me comunico muy bien con mis alumnos, al final la clave está ahí, no importa tanto el conocimiento que tengamos sino el cómo lo transmitimos, entonces, creo que lo que hace al docente ser un buen docente es que llegue a los alumnos. Yo creo que lo que más feliz me hace es encontrarme hoy en día con una jueza, por ejemplo, que ha sido alumna mía, y que me diga, “oye, pues a mí derecho procesal me gustó mucho”, porque mi asignatura puede ser un rollo, con trámites, plazos… y que te digan que les gustó, pues me hace pensar que llegué a comunicar bien lo que quería transmitir.


Documento:		4. Ciencias Sociales y Jurídicas\P20 CCSS Creencias
Peso:	0
Posición:	30 - 31
Código:	1. Creencias\Rol docente y actitudes\1.3. Características
E: Claro. Y hablando del rol docente y las actitudes, ¿podrías decirnos algunas características que creas que te definen como docente?
P20: Eso no te lo sé decir, pero vamos, yo estoy muy contenta con la labor docente. De hecho, he sido nombrada bastantes años como madrina de promoción y demás, entonces, son esas pequeñas alegrías que te llevas a casa, que no se te reconocen en ningún sitio ni se ponen en ningún currículum, pero que te las llevas, que son internas, o sea, que bien, que yo estoy contenta.


Documento:		4. Ciencias Sociales y Jurídicas\P21 CCSS Creencias
Peso:	0
Posición:	44 - 47
Código:	1. Creencias\Rol docente y actitudes\1.3. Características
E: Estupendo. Y, ¿podría decirme algunas características que lo definan como docente?
P21: ¿Que me definan como docente? Pues bueno, yo siempre he dicho, cuando me vengo abajo, que lo que me define es la vocación, en el sentido de que para ser docente hay que tener vocación, porque como no la tengas…yo creo que, en muchos ámbitos, pero siempre he tenido muy clara mi vocación docente, igual que reconozco que no tengo vocación investigadora, en el sentido de que sea lo que más me motive y tal, pero sí, sí. Y, de hecho, yo soy periodista de profesión, pero siempre estuve entre hacer magisterio o periodismo porque yo pensaba que la docencia era una maravilla, y acabé compatibilizando ambas profesiones y nunca he abandonado la docencia. No solo en la facultad, puse clases en el instituto, cuando todavía no había facultad, ya daba yo clases en el centro privado de periodismo porque me encantaba dar clases, y, si hay algo que me caracteriza es la vocación docente, y aparte ya, según las épocas, hay veces que está uno…
E: En esta es seguro que sé cómo está usted.
P21: Sí, sí.


Documento:		4. Ciencias Sociales y Jurídicas\P22 CCSS Creencias
Peso:	0
Posición:	26 - 29
Código:	1. Creencias\Rol docente y actitudes\1.3. Características
E: Claro. Y, ¿podría decirme algunas características que crea usted que tiene como docente?
P22: ¿Con respecto a los alumnos discapacitados?
E: No, en general, como docente.
P22: Bueno, yo creo que es lo tendrían que decir los alumnos. Yo me considero un poco recta, un poco dura, exigente, y sí, más o menos eso. Yo creo que luego, cuando conozco a la gente soy muy cariñosa, pero, en principio soy seca, siempre soy seca.


Documento:		4. Ciencias Sociales y Jurídicas\P23 CCSS Creencias
Peso:	0
Posición:	36 - 37
Código:	1. Creencias\Rol docente y actitudes\1.3. Características
E: Respecto al rol docente y a actitudes, ¿podría decirme algunas características que la definan como docente?
P23: Yo creo que soy cercana con los alumnos porque, pues igual que con un alumno con discapacidades, pues me fijo si uno se cortó el pelo, si uno se lo pintó, le pregunto, a alguno lo veo triste y le digo “¿qué te pasa? ¿tienes problemas en tu casa?” O sea, que tanto a los que tienen cierta discapacidad como a los que no tienen, pues soy muy cercana con ellos. Les pregunto muchas cosas, me intereso mucho por su vida personal porque me parece que está muy vinculada al desempeño en la asignatura.


Documento:		4. Ciencias Sociales y Jurídicas\P24 CCSS Creencias
Peso:	0
Posición:	24 - 25
Código:	1. Creencias\Rol docente y actitudes\1.3. Características
E: Claro. La siguiente pregunta va en relación al rol docente y las actitudes, ¿podrías decirnos algunas características que creas que te definen como docente?
P24: Es lo que más nos cuesta a los españoles porque nuestro sistema educativo no nos ha enseñado a valorarlo. Yo cuando llego a clase lo primero que les digo es que cojan una cartulina y pongan el nombre en grande porque yo tengo miopía y no solamente por eso, sino porque normalmente tendemos al individualismo, a la frialdad, a no saber el nombre, como si fueran un número… Entonces, ponen su nombre en grande y creo que fortalece hasta…yo siempre digo que para mí es primordial que sean grandes técnicos, pero con un máster de humanidad. Entonces, esa humanidad es la que intento transmitir. Como profesora también es fundamental todo lo teórico que tengan que aprender, pero yo veo al estudiante en su conjunto, no solamente es meter información ahí como una máquina, no en sentido despectivo, sino una máquina que va a elaborar conocimientos. No, no, es algo más. Intento que también se enamoren de la asignatura, es primordial que disfruten aprendiendo y la asignatura la construimos entre todos. Hombre, yo la llevo al 100%, pero otras veces ellos el 50 o el 70, así vamos negociando. Pero yo lo llevo todo preparado, pero no lo saco todo a la palestra porque si no son agentes pasivos, como el modelo antiguo que todo el mundo está ahí…que el estudiante está desconectado a los 15 min. Entonces, estoy muy satisfecha, hay uno de mis trabajos, por lo visto estrella, que es muy alabado por los estudiantes y son las historias de vida, que siempre, siempre me dicen que la siguiente promoción que entre tiene que conocer la actividad, que se trabaja muchísimo, pero vale la pena. Mi gran objetivo es tocar emociones, cuando lo haces, se produce un cambio magnífico porque deja entrar la información en la persona, se transforma, hablan del tema con normalidad con la familia, los amigos… Es muy gratificante esa parte e intento ser cercana. Cuando llego a clase les digo cómo me llamo y que me hablen de tú. Yo creo que todos y cada uno de nosotros sabemos dónde están nuestros límites y el tú o el usted no nos va a diferenciar de nada. Está bastante bien valorada hasta ahora la asignatura, de hecho, la han cogido este año como experimento piloto en la complutense, entonces digo “este año que lo han cogido, siempre tenemos que darlo, pero este año especialmente”. Creo que es la cercanía…también estoy muy criticada por los compañeros porque hay veces que dice que extralimito…yo creo que son miradas diferentes de la pedagogía, porque la distancia, el hacer esperar, el no contestar correos… No es mi modelo. Me siento muy identificada cuando encuentro profesores que son de la misma línea. 


Documento:		4. Ciencias Sociales y Jurídicas\P24 CCSS Creencias
Peso:	0
Posición:	27 - 27
Código:	1. Creencias\Rol docente y actitudes\1.3. Características
Para mí es que prácticamente es como estar de vacaciones, porque me encanta lo que hago, aunque me lleve muchas horas en el ordenador y cuando trabajo, pero disfruto


Documento:		4. Ciencias Sociales y Jurídicas\P24 CCSS Creencias
Peso:	0
Posición:	35 - 35
Código:	1. Creencias\Rol docente y actitudes\1.3. Características
Eso es interesante. Entonces, intento que les llegue, ser empática, adaptarme…


Documento:		4. Ciencias Sociales y Jurídicas\P25 CCSS Creencias
Peso:	0
Posición:	44 - 45
Código:	1. Creencias\Rol docente y actitudes\1.3. Características
E: Hablando ahora como profesora, ¿podrías decirme alguna característica que te defina a ti como docente?
P25: Yo creo que soy una profesora que preparo bastante bien lo que quiero transmitir al alumno, por lo tanto, trabajo bastante la materia, soy muy ordenada en la exposición de los contenidos, identifico muy bien qué quiero decir en cada clase, y por dónde no quiero entrar, y luego, creo que transmito muy bien. Creo que tengo sensibilidad para captar al alumnado, para verlo, para percibirlo, intuición para ver por dónde van, para saber meter una broma o para ponerme seria, y…yo creo que es eso.
