# Supplementary material for: Is pedagogical training an essential requirement for inclusive education? The case of faculty members in the area of Social and Legal Sciences in Spain
Source: PLoS One. 2021 Jul 2;16(7):e0254250. doi: 10.1371/journal.pone.0254250 (PMC8253417; doi:10.1371/journal.pone.0254250)
Supplement: Attachment — Submitted filename: Response to Reviewers.docx [file pone.0254250.s003.docx]

Thank you for the review. We appreciate the opportunity to review this manuscript and improve its quality. Foremost, we would like to thank the editor and reviewers for their constructive feedback. As you can see, we have made significant changes in our work. We apologize for the time it took to send in the revision of the paper, but we wanted to analyze and review in depth each of the comments.

We have completed a revision of this manuscript based on the comments and suggestions given, which we believe has improved the paper. Please see below our responses to specific comments. We have marked all changes to the original manuscript with *track changes*.

| **COMMENTS** | **CHANGES** |
| --- | --- |
| Thank you for allowing me to review this valuable work. I appreciate the research questions you aimed to answer. You provide some excellent insight into the perceptions and practices of university faculty regarding students with disabilities. Your work contributes to the current literature and scholarship in this area and helps the field of inclusive education move forward. Below are my recommendations to prepare this manuscript for publication. | We appreciate these comments, which encourage us to improve our study. We think that this paper can be very useful for academics and help universities to move towards inclusive education. |
| Line 51: extra words (to the)  Line 53: “turning into an inclusive faculty member”- maybe relace ‘turning into’ with ‘becoming’  Line 73: should say ‘students with disabilities” | We have made these changes. Following this comment, we have asked a translator to revise the paper. |
| Line 247-248- I think you should end your section with something other than a quotation. Could you include your quotations within the paragraphs rather than always at the end? | We have changed the end this section according to the reviewer's indications. |
| In various sentences in the results section you indicate that ‘the faculty members’ indicated something. It would strengthen your results if you could provide some clearer indication of how a result was selected. This could be discussed in the data analysis, perhaps when you talk about the methods of analysing this data. Out of 25 participants, how many had to mention the term in your analysis software in order to be included as one of the codes? Using terminology such as “most, over half, few, etc. would be helpful. Which you actually do later on. | We have changed this section according to the reviewer's indications. |
| In the discussion section, making explicit reference to how this research connects to prior research would better support the claims you make, rather than simple listing the references at the end of a statement about your findings. | We have made these changes throughout the discussion as indicated by the reviewer. |
| 448-450- I don’t think you can make this claim that these universities do not align with former research reporting a medical model since you have 25 faculty over 7 Universities, who were nominated for being inclusive educators. It would be important to reframe this point so that it better represents how your research fits within the research you are citing that indicates a medical model is still evident in many Universities. I believe that your work plays an important part in moving away from this but I do not believe the findings to be contradictory given your sample of participants.  You make a similar claim again in 464-466 (same comments as above regarding framing this within the limitations of the current study) | We have changed this confusing expression. We have now included this aspect differently and in a more detailed way. Indeed, there are studies that state that the medical model is still present in higher education institutions, which is why our study can contribute to moving towards the social model of disability. |
| 473- The first shortcoming was related to the support provided by the disability services to make the reasonable adjustments an adequate manner- this sentence needs revising, perhaps there are a few words missing.  Although you mention a few recommendations for moving forward throughout your discussion and conclusion, this should be a section on its own to highlight your recommendations based on your findings and where you would see research extending on these findings. Then follow with your conclusion of the overall findings as they relate to the bigger picture within the research. | We have revised this to make the sentence more meaningful. We have also included a section on recommendations and future research. |
| 554- who is Frank et al? are you missing some information? | This is a mistake. We have now included the full reference. Taking this comment into account, we have checked and verified again all references in the entire paper. |
| 453-455- If you make this claim, you should support it more with a connection to your study. It is a bold statement that training would fix the challenges faced by faculty members, but there is an entire body of research that supports the influence of ones (educators) attitudes and beliefs about disability and opportunities for positive experience with ppl with disabilities that contribute to their inclusive practice. | We have modified this claim. We believe that the link to our research can now be perceived in a better way. |
