# Supplementary material for: Is pedagogical training an essential requirement for inclusive education? The case of faculty members in the area of Social and Legal Sciences in Spain
Source: PLoS One. 2021 Jul 2;16(7):e0254250. doi: 10.1371/journal.pone.0254250 (PMC8253417; doi:10.1371/journal.pone.0254250)
Supplement: Attachment — Submitted filename: Response to Reviewers second revision.docx [file pone.0254250.s004.docx]

Thank you for the second review. We have completed a revision of this manuscript based on your comments and suggestions, which we believe has improved the paper for further consideration for publication.

Please see below our response to your specific comments. We have included any changes to the original manuscript using the *track changes* mode.

| **COMMENTS** | **CHANGES** |
| --- | --- |
| 298 This assertion leads us to question whether training is not part of the foundations of inclusive practices.- this sentence is confusing and should consider rewriting for clarity- “This assertion leads us to question if disability training is part of the foundations of inclusive education” | We have rewritten this sentence. |
| 353 Should read “More than half” not Most than half | We have made this change. |
| 361 One of these actions "consisted in" giving the contents to the students in advance.- should be “consisted of” | We have made this change. |
| 411 change to: This would "promote" awareness of how difficulties encountered by students were actually imposed by the institution and not an intrinsic characteristic of people with disabilities. | We have made this change. |
| 509 replaced "to" with "in" improvement, reveal clues to prevent the violation of the right of people with disabilities "in" HE. | We have replaced this expression. |
