# Supplementary material for: Is pedagogical training an essential requirement for inclusive education? The case of faculty members in the area of Social and Legal Sciences in Spain
Source: PLoS One. 2021 Jul 2;16(7):e0254250. doi: 10.1371/journal.pone.0254250 (PMC8253417; doi:10.1371/journal.pone.0254250)
Supplement: Attachment — Submitted filename: PONE-D-21-01964 Reviewer Comments.docx [file pone.0254250.s002.docx]

PONE-D-21-01964 Reviewer Comments

Thank you for allowing me to review this valuable work. I appreciate the research questions you aimed to answer. You provide some excellent insight into the perceptions and practices of university faculty regarding students with disabilities. Your work contributes to the current literature and

Line 51: extra words (to the)

Line 53: “turning into an inclusive faculty member”- maybe relace ‘turning into’ with ‘becoming’

Line 73: should say ‘students with disabilities”

Line 247-248- I think you should end your section with something other than a quotation. Could you include your quotations within the paragraphs rather than always at the end?

In various sentences in the results section you indicate that ‘the faculty members’ indicated something. It would strengthen your results if you could provide some clearer indication of how a result was selected. This could be discussed in the data analysis, perhaps when you talk about the methods of analysing this data. Out of 25 participants, how many had to mention the term in your analysis software in order to be included as one of the codes? Using terminology such as “most, over half, few, etc. would be helpful. Which you actually do later on.

In the discussion section, making explicit reference to how this research connects to prior research would better support the claims you make, rather than simple listing the references at the end of a statement about your findings.

448-450- I don’t think you can make this claim that these universities do not align with former research reporting a medical model since you have 25 faculty over 7 Universities, who were nominated for being inclusive educators. It would be important to reframe this point so that it better represents how your research fits within the research you are citing that indicates a medical model is still evident in many Universities. I believe that your work plays an important part in moving away from this but I do not believe the findings to be contradictory given your sample of participants.

You make a similar claim again in 464-466 (same comments as above regarding framing this within the limitations of the current study)

473- The first shortcoming was related to the support provided by the disability services to make the reasonable adjustments an adequate manner- this sentence needs revising, perhaps there are a few words missing.

Although you mention a few recommendations for moving forward throughout your discussion and conclusion, this should be a section on its own to highlight your recommendations based on your findings and where you would see research extending on these findings. Then follow with your conclusion of the overall findings as they relate to the bigger picture within the research.

554- who is Frank et al? are you missing some information?

453-455- If you make this claim, you should support it more with a connection to your study. It is a bold statement that training would fix the challenges faced by faculty members, but there is an entire body of research that supports the influence of ones (educators) attitudes and beliefs about disability and opportunities for positive experience with ppl with disabilities that contribute to their inclusive practice.
